# IFN-α and IFN-β inhibit NLRP1-driven PANoptosis

**DOI:** 10.1038/s44319-026-00888-0

**Published:** 2026-07-29

**Authors:** Bhesh Raj Sharma, Harisankeerth Mummareddy, Sangappa B Chadchan, Roman Sarkar, Chadi A EI Farran, Thirumala-Devi Kanneganti

**Affiliations:** Department of Immunology, St. Jude Children’s Research Hospital, Memphis, TN 38105 USA

**Keywords:** Autophagy & Cell Death, Immunology, Signal Transduction

## Abstract

Pathogens, tissue damage, and cellular stress are detected by innate immune sensor molecules to drive inflammatory signaling and cell death. Mutations in the sensor NLRP1 are associated with inflammatory disease, but the regulation of this sensor is not well understood. Here, we find that LPS, a TLR4 ligand and canonical activator of innate immunity, inhibits NLRP1-mediated caspase activation, IL-18 release, and inflammatory cell death, PANoptosis. This inhibition requires TRIF but not MyD88, implicating TRIF-dependent TLR signaling. IRF3 is also required, suggesting an essential role for type I IFN signaling. Indeed, IFN-β production or treatment with exogenous IFN-α or IFN-β inhibits NLRP1-dependent PANoptosis in mouse bone marrow-derived macrophages and human macrophages and monocytes. Mechanistically, *Nlrp1b*/*NLRP1* expression is significantly reduced in LPS- or type I IFN-treated cells. Overall, our study identifies a TLR4–TRIF–IRF3 signaling axis that induces type I IFNs to negatively regulate *NLRP1* transcription, thereby blocking NLRP1-driven, caspase-1/caspase-8/RIPK3–dependent PANoptosis. These findings suggest type I IFNs as a potential therapeutic strategy for NLRP1-driven inflammatory diseases.

## Synopsis

In contrast to its canonical inflammatory effects, lipopolysaccharide (LPS) drives type I IFN production to inhibit NLRP1-mediated caspase-1/caspase-8/RIPK3-dependent PANoptosis. IFN negatively regulates NLRP1 transcription, thereby inhibiting PANoptosis.

- NLRP1-mediated PANoptosis is blocked by LPS-driven type I IFN production that occurs via TLR4–TRIF–IRF3 signaling.
- Type I IFNs inhibit NLRP1 transcription, thereby blocking NLRP1-mediated PANoptosis in macrophages.
- Exogenous treatment with type I IFNs is a potential therapeutic strategy in NLRP1-driven inflammatory diseases.

## Abstract (web summary)

Contrasting its canonical inflammatory effects, lipopolysaccharide (LPS) drives type I IFN production to inhibit NLRP1-mediated caspase-1/caspase-8/RIPK3-dependent PANoptosis. IFN negatively regulates NLRP1 transcription, thereby inhibiting PANoptosis.

## Graphical abstract

**Figure:**
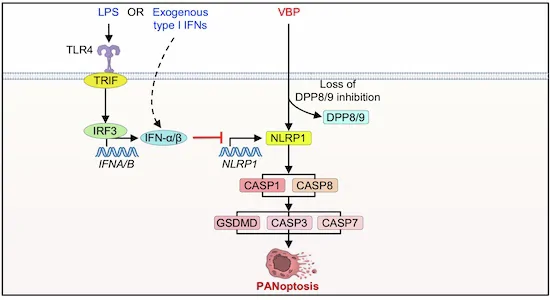

**Figure:**
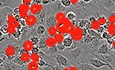

## Introduction

The innate immune system is the body’s first line of defense against infection and homeostatic alterations (Beutler, 2004; Janeway, 1989; Kanneganti, 2020). Pathogens, pathogen- and damage-associated molecular patterns (PAMPs and DAMPs), and homeostatic disruptions are sensed by nucleotide-binding oligomerization domain-like receptors (NLRs), which are cytosolic germline-encoded pattern recognition receptors (PRRs) that promote inflammation and lytic cell death (Kanneganti, 2020; Takeuchi and Akira, 2010). Several NLRs form cell death-inducing multiprotein complexes such as inflammasomes or larger PANoptosome complexes, which can have inflammasomes as integral components (Sharma et al, 2025; Sundaram et al, 2024; Sundaram et al, 2023). The inflammasome machinery is responsible for activating caspase-1 to drive the maturation and release of the proinflammatory cytokines IL-1β and IL-18 (Kanneganti, 2020; Kanneganti et al, 2006; Martinon et al, 2002; Martinon et al, 2001); caspase-1 can also act with other PANoptosome components, such as caspase-8 and/or RIPKs, to drive PANoptotic cell death (Christgen et al, 2020; Hou et al, 2024; Karki et al, 2022; Karki et al, 2020; Karki et al, 2021a; Karki et al, 2021b; Kuriakose et al, 2016; Lee et al, 2021; Sundaram et al, 2024). Lytic cell death through PANoptosis can clear pathogens and other threats, but it can also promote inflammation and pathology, making the tight regulation of innate immune sensors and their downstream cell death signaling critical.

One of the first inflammasome sensors to be discovered was NLRP1. NLRP1 has a unique domain structure among the NLRs in that it contains a function-to-find domain (FIIND) and a C-terminal caspase activation and recruitment domain (CARD); this domain structure contributes to its complex regulatory mechanisms (Finger et al, 2012; Zhang et al, 2023). Autoproteolysis in the FIIND generates two fragments that remain associated, causing NLRP1 autoinhibition (Frew et al, 2012). The inactive form is stabilized by dipeptidyl peptidases 8 and 9 (DPP8/9) (Hollingsworth et al, 2021); hence, NLRP1 signaling can be activated by inhibiting DPP8/9, such as by using the small molecule Val-boroPro (VBP) (Okondo et al, 2017; Okondo et al, 2018; Zhong et al, 2018). This leads to inflammasome assembly, caspase-1 activation, and lytic cell death (Chavarría-Smith et al, 2016; Chavarría-Smith and Vance, 2013; Chui et al, 2019; Levinsohn et al, 2012; Sandstrom et al, 2019; Xu et al, 2019). Accordingly, mutations in *NLRP1* can lead to aberrant activation and are associated with several clinically intractable autoimmune and autoinflammatory diseases and cancers, including multiple sclerosis, systemic sclerosis, vitiligo, rheumatoid arthritis, Crohn’s disease, and melanoma (Dieude et al, 2011; Fenini et al, 2022; Jin et al, 2010; Maver et al, 2017; Sui et al, 2012; Verma et al, 2012; Zhong et al, 2016; Zurawek et al, 2010). However, the full regulatory mechanisms controlling NLRP1 activation physiologically remain unclear. Furthermore, blocking aberrant NLRP1 activity is a therapeutically attractive strategy to mitigate chronic inflammation, but no treatment that specifically blocks NLRP1 activation has yet been clinically evaluated.

Therefore, we sought to understand the molecular mechanisms of NLRP1 activation and identify potential therapeutic targets in this pathway. We found that, in contrast to its canonical role as a PAMP and activator of innate immunity, lipopolysaccharide (LPS) inhibited NLRP1-driven inflammatory cell death, PANoptosis, in response to VBP. Mechanistically, LPS signaled through toll-like receptor 4 (TLR4), TIR-domain-containing adapter-inducing interferon-β (TRIF), and interferon regulatory factor 3 (IRF3) to block NLRP1-mediated PANoptosis. We further demonstrated that LPS triggered the type I IFN production, which was necessary and sufficient to inhibit *NLRP1* transcription and block NLRP1-mediated, caspase-1/caspase-8/RIPK3–dependent PANoptosis. These findings highlight the therapeutic potential of exogenous type I IFNs to treat inflammatory diseases driven by aberrant activation of NLRP1.

## Results and discussion

### LPS inhibits NLRP1-mediated PANoptosis

**Figure 1:**
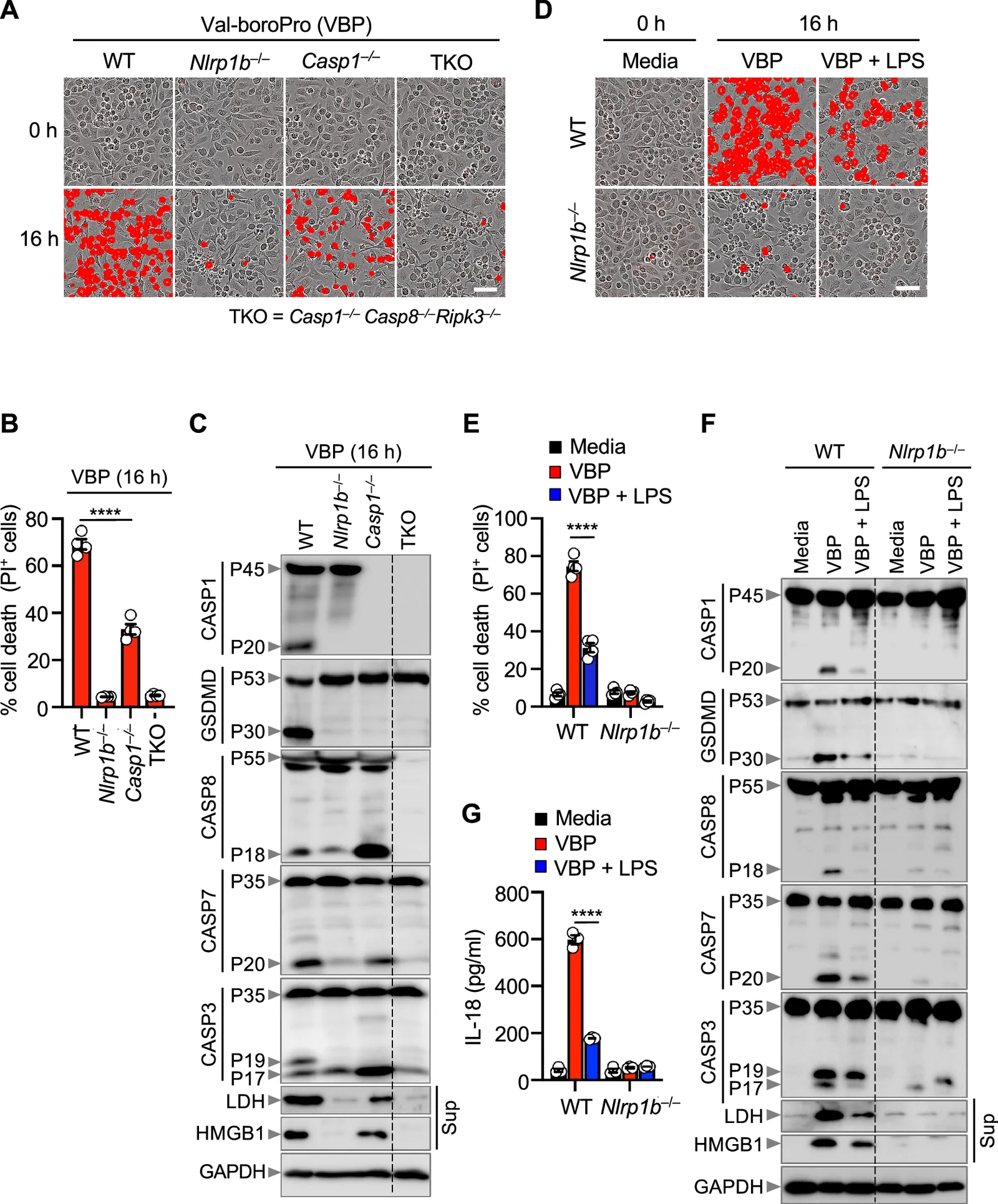
LPS inhibits NLRP1-mediated PANoptosis. (**A**,** B**) Representative images (**A**) and quantification (**B**) of cell death in wild type (WT), *Nlrp1b*^*–/–*^, *Casp1*^*–/–*^, and *Casp1*^*–/–*^*Casp8*^*–/–*^*Ripk3*^*–/–*^ (TKO) mouse bone marrow-derived macrophages (BMDMs) at the indicated timepoints after treatment with Val-boroPro (VBP). (**C**) Immunoblot analysis of pro- (P45) and cleaved (P20) caspase-1 (CASP1), pro- (P53) and activated (P30) gasdermin D (GSDMD), pro- (P55) and cleaved (P18) caspase-8 (CASP8), pro- (P35) and cleaved (P20) caspase-7 (CASP7), and pro- (P35) and cleaved (P19 and P17) caspase-3 (CASP3) in WT, *Nlrp1b*^*–/–*^, *Casp1*^*–/–*^, and TKO BMDMs 16 h after treatment with VBP. LDH and HMGB1 were assessed from the supernatant (Sup). GAPDH was used as a loading control; pro- and cleaved forms of caspases and GSDMD were analyzed together to serve as an internal control. Immunoblot samples were derived from the same experiment and gels/blots were processed in parallel. (**D**,** E**) Representative images (**D**) and quantification (**E**) of cell death in WT and *Nlrp1b*^*–/–*^ mouse BMDMs at the indicated timepoints (**D**) or at 16 h (**E**) after treatment with media, VBP, or VBP plus lipopolysaccharide (LPS). (**F**) Immunoblot analysis of CASP1, GSDMD, CASP8, CASP7, CASP3, LDH, and HMGB1 in WT and *Nlrp1b*^*–/–*^ BMDMs at 16 h after treatment with media, VBP, or VBP plus LPS. Loading controls used as described for (**C**). (**G**) IL-18 concentration in the supernatant of BMDMs at 16 h after treatment with media, VBP, or VBP plus LPS. (**A**–**G**) Data were representative of five independent biological replicates for (**A**, **B**, **D**, **E**) and three independent biological replicates for (**C**, **F**, **G**), *n* = 4 (**B**, E) or 3 (**G**) technical replicates. Scale bar: 50 μm (**A**,** D**). ****P < 0.0001. One-way ANOVA with Dunnett’s multiple comparison (**B**) and two-way ANOVA with Sidak’s multiple comparison test (**E**,** G**) were used. The data were represented as mean ± SEM in (**B**, **E**, **G**). Source data are available online for this figure.

Innate immune signaling involves the activation of PRRs to protect the host from invading pathogens and sterile insults (Kanneganti, 2020). Aberrant NLRP1 activation can drive excess inflammation and pathology, yet NLRP1 regulation and NLRP1 cell death signaling are not well understood. As a result, no specific inhibitors for NLRP1 have reached clinical trials. Therefore, to understand the regulation of NLRP1-mediated cell death and inform future therapeutic strategies, we used genetic models to investigate NLRP1 signaling. We found that *Casp1*^–/–^ BMDMs were only partially protected from VBP-induced cell death (Fig. 1A,B). By contrast, BMDMs deficient in the sensor NLRP1 or deficient in PANoptosis machinery (*Casp1*^–/–^*Casp8*^–/–^*Ripk3*^–/–^; TKO) were almost completely protected (Fig. 1A,B). We evaluated biochemical markers associated with PANoptosis and found that in WT BMDMs, VBP induced the activation of caspase-1, GSDMD, caspase-8, caspase-7, and caspase-3, along with the release of the DAMPs lactate dehydrogenase (LDH) and high mobility group box 1 (HMGB1); caspase-8, caspase-7, and caspase-3 activation, as well as LDH and HMGB1 release, were also observed in *Casp1*^–/–^ BMDMs (Fig. 1C). These molecules were not activated or released in TKO BMDMs (Fig. 1C). Collectively, this genetic and biochemical evidence indicates that VBP triggers NLRP1-dependent PANoptosis.

**Figure EV1:**
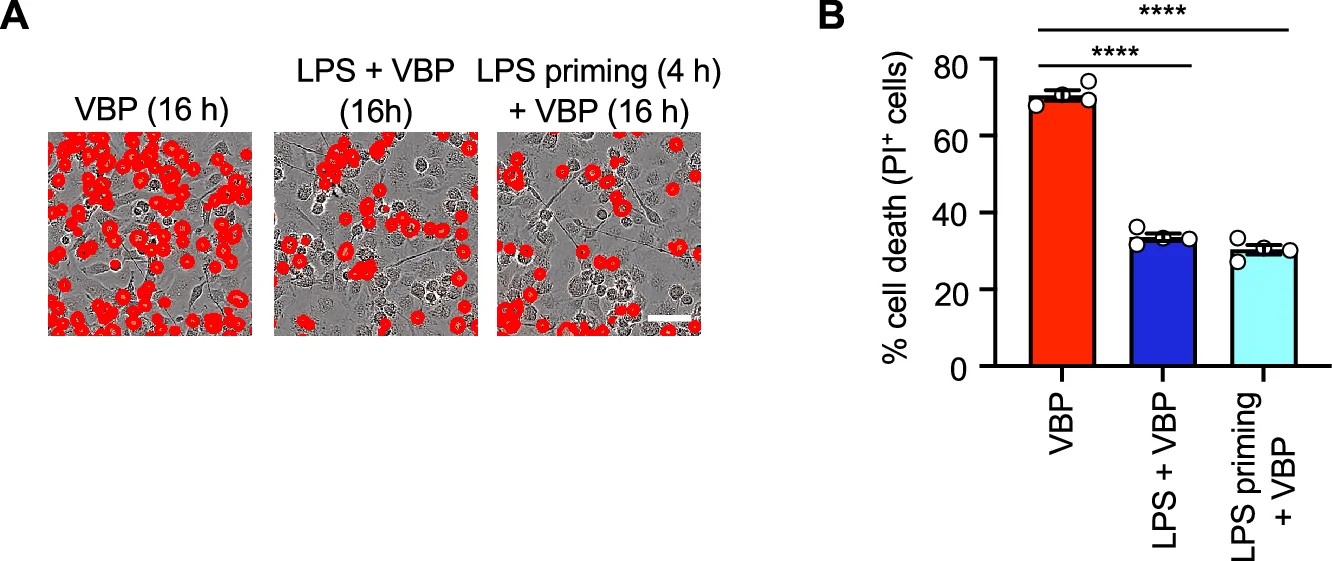
LPS priming inhibits VBP-induced cell death. (**A**,** B**) Representative images (**A**) and quantification (**B**) of cell death in wild-type mouse bone marrow-derived macrophages (BMDMs) at 16 h after treatment with Val-boroPro (VBP), simultaneous lipopolysaccharide (LPS) plus VBP, or LPS (4 h priming) followed by VBP. (**A**,** B**) Data were representative of three independent biological replicates. (**B**) *n* = 4 four technical replicates. Scale bar: 50 μm (**A**). ****P < 0.0001. One-way ANOVA with Dunnett’s multiple comparison test was used. The data were represented as mean ± SEM in (**B**). Source data are available online for this figure.

To understand the upstream regulation of NLRP1, we then assessed the role of TLR activation, which can induce the expression of other NLRs through innate immune priming and promote cell death in many contexts (Sharma et al, 2023; Sundaram et al, 2024). In contrast to its canonical role in inducing inflammation, the TLR4 activator LPS inhibited VBP-induced cell death, both when applied together with VBP (Fig. 1D,E) and when given as a priming stimulus 4 h before VBP (Fig. EV1). In line with the reduction in cell death, we found that the addition of LPS reduced the VBP-induced activation of caspase-1, GSDMD, caspase-8, caspase-7, and caspase-3, and release of LDH and HMGB1 (Fig. 1F). LPS also reduced the VBP-mediated release of IL-18 (Fig. 1G). These results suggest that LPS blocked VBP-induced PANoptosis.

**Figure EV2:**
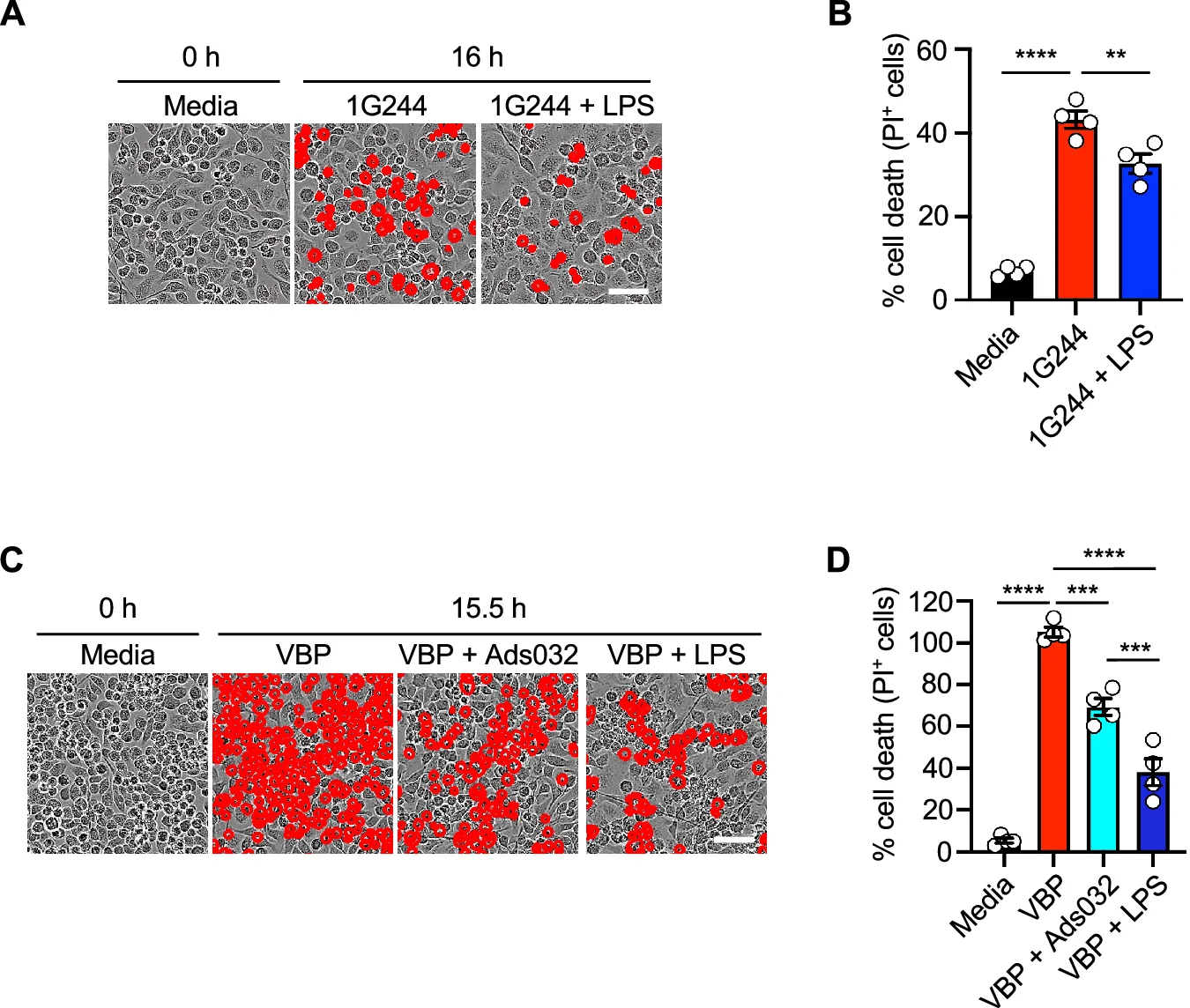
LPS protects against NLRP1-mediated cell death and is more potent than the NLRP1 inhibitor Ads032. Representative images (**A**,** C**) and quantification (**B**,** D**) of cell death in wild-type (WT) mouse bone marrow-derived macrophages (BMDMs) in media (0 h) or after treatment with (**A**,** B**) 1G244 or 1G244 plus lipopolysaccharide (LPS) for 16 h, or (**C**,** D**) Val-boroPro (VBP), VBP plus Ads032, or VBP plus LPS for 15.5 h. (**A**–**D**) Data were representative of three independent biological replicates. (**B**,** D**) *n* = 4 technical replicates. Scale bar: 50 μm (**A**,** C**). (**B**) Media vs 1G244, **** *P* < 0.0001; 1G244 vs 1G244 + LPS, **P = 0.0049. (**D**) Media vs VBP, **** *P* < 0.0001; VBP vs VBP + Ads032, ***P = 0.0002; VBP vs VBP + LPS, ****P < 0.0001; VBP + Ads032 vs VBP + LPS, ***P = 0.0006. One-way ANOVA with (**B**) Dunnett’s and (**D**) Tukey’s multiple comparison test were used. The data were represented as mean ± SEM in (**B**, **D**). Source data are available online for this figure.

To confirm that this effect was NLRP1-specific and not VBP-specific, we first confirmed that NLRP1b-deficient BMDMs were protected from cell death in response to both VBP and VBP plus LPS (Fig. 1D–F). Additionally, we used a different NLRP1-activating ligand, 1G244; cell death induced by 1G244 was also significantly inhibited by LPS (Fig. EV2A,B). Furthermore, we compared the inhibitory effects of LPS on cell death with those of the recently discovered small molecule Ads032, a dual NLRP1 and NLRP3 inhibitor (Docherty et al, 2023). We found that LPS was a more potent inhibitor of VBP-induced cell death than Ads032 (Fig. EV2C,D).

**Figure EV3:**
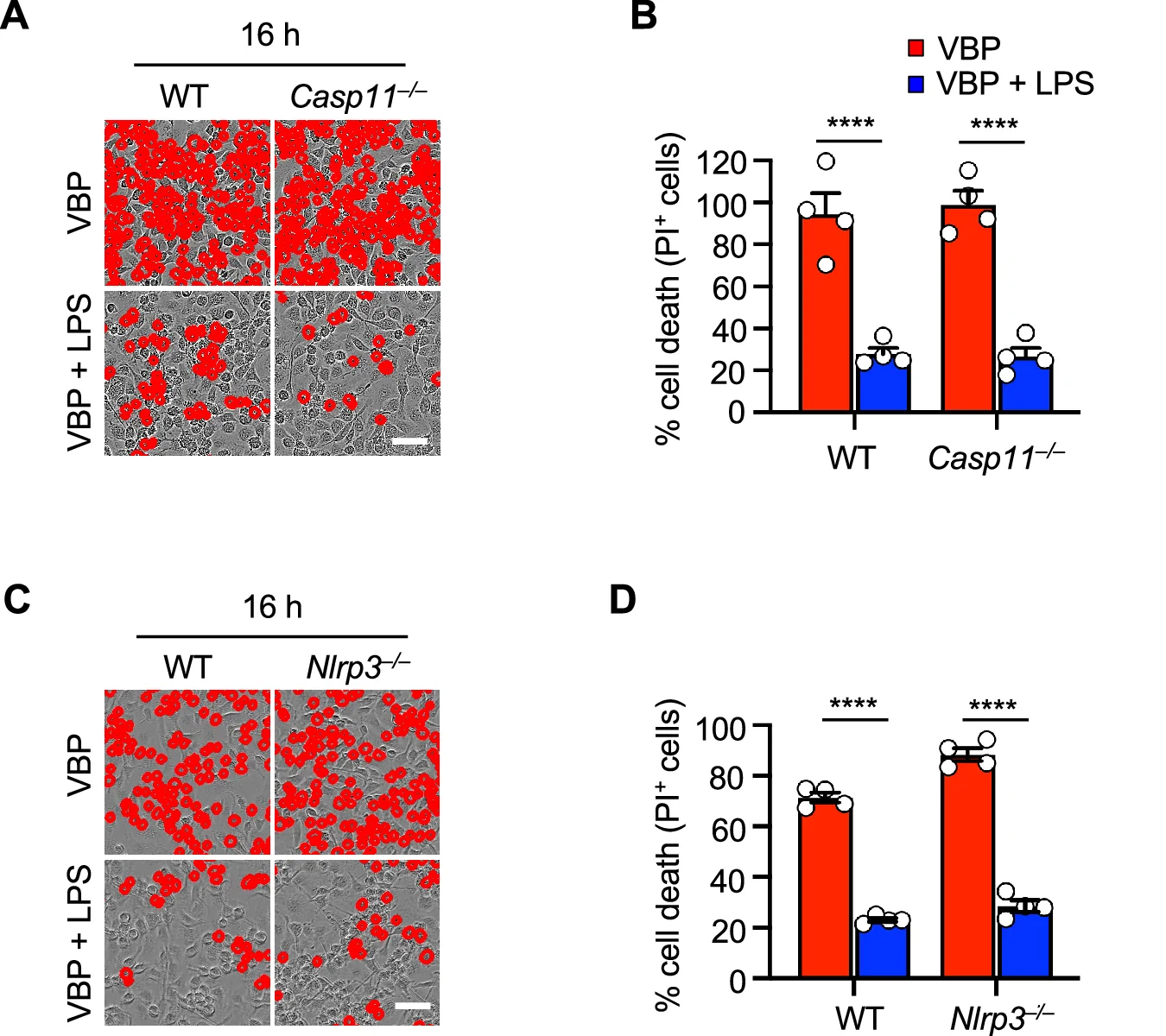
Caspase-11 and NLRP3 are not required for LPS to block VBP-induced cell death. (**A**,** B**) Representative images (**A**) and quantification (**B**) of cell death in wild type (WT) and *Casp11*^*–/–*^ mouse bone marrow-derived macrophages (BMDMs) at 16 h after treatment with Val-boroPro (VBP) or VBP plus lipopolysaccharide (LPS). (**C**,** D**) Representative images (**C**) and quantification (**D**) of cell death in WT and *Nlrp3*^*–/–*^ BMDMs at 16 h after treatment with VBP or VBP plus LPS. (**A**–**D**) Data were representative of three independent biological replicates. (**B**,** D**) *n* = 4 technical replicates. Scale bar: 50 μm (**A**,** C**). ****P < 0.0001 (two-way ANOVA with Sidak’s multiple comparison test). The data were represented as mean ± SEM in (**B**, **D**). Source data are available online for this figure.

It has been reported that LPS upregulates and directly binds caspase-11, triggering pyroptosis and non-canonical NLRP3 inflammasome activation (Hagar et al, 2013; Kayagaki et al, 2011; Kayagaki et al, 2013; Sharma and Kanneganti, 2021). Since both caspase-11 and NLRP1 have CARDs, we hypothesized that the caspase-11 CARD may interact with the NLRP1 CARD to inhibit NLRP1-mediated cell death in response to LPS. However, we found that caspase-11 was not required for LPS to inhibit NLRP1-induced cell death (Fig. EV3A,B). NLRP3 was also dispensable (Fig. EV3C,D). Overall, these data suggest that LPS inhibits NLRP1-mediated PANoptosis through a previously unknown mechanism.

### TLR–TRIF signaling axis inhibits NLRP1-mediated PANoptosis

**Figure 2:**
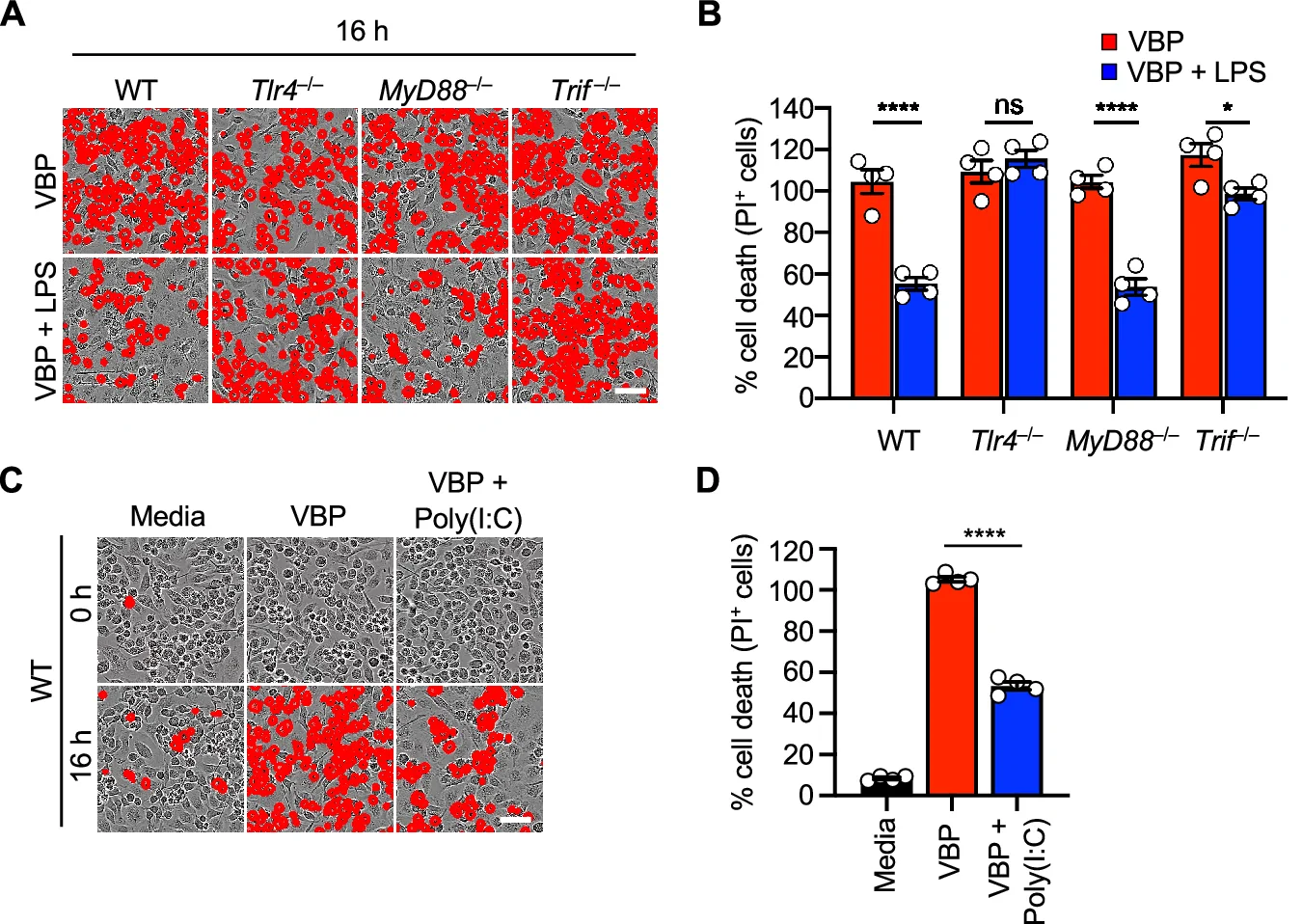
TLR–TRIF signaling axis inhibits NLRP1-mediated PANoptosis. (**A**,** B**) Representative images (**A**) and quantification (**B**) of cell death in wild type (WT), *Tlr4*^*–/–*^*, MyD88*^*–/–*^, and *Trif*^*–/–*^ mouse bone marrow-derived macrophages (BMDMs) at 16 h after treatment with Val-boroPro (VBP) or VBP plus lipopolysaccharide (LPS). (**C**,** D**) Representative images (**C**) and quantification (**D**) of cell death in WT BMDMs in media or at the indicated timepoints (**C**) and at 16 h (**D**) after treatment with VBP or VBP plus poly(I:C). (**A**–**D**) Data were representative of three independent biological replicates. (**B, D**) *n* = 4 technical replicates. Scale bar: 50 μm (**A**,** C**). (**B**) WT VBP vs VBP + LPS, ****P < 0.0001; *Tlr4*^*–/–*^ VBP vs VBP + LPS, *P* = 0.7873 (ns); *MyD88*^*–/–*^ VBP vs VBP + LPS, ****P < 0.0001; *Trif*^*–/–*^ VBP vs VBP + LPS, *P = 0.0228. (**D**) VBP vs VBP + poly(I:C), ****P < 0.0001. Two-way ANOVA with Sidak’s multiple comparison test (**B**) and one-way ANOVA followed by Dunnett’s multiple comparison test (**D**) were used. The data were represented as mean ± SEM in (**B**, **D**). Source data are available online for this figure.

Canonical LPS signaling occurs via TLR4 through the adapters MyD88 and TRIF (Beutler, 2004; Takeuchi and Akira, 2010). We found that robust cell death occurred in WT, *Tlr4*^–/–^, *MyD88*^–/–^, and *Trif*^–/–^ BMDMs in response to VBP (Fig. 2A,B). The addition of LPS inhibited this cell death in WT and *MyD88*^–/–^ BMDMs, but not in *Tlr4*^–/–^ BMDMs. While LPS did induce a statistically significant decrease in cell death in *Trif*^–/–^ cells, this difference was small (Fig. 2A,B). These data suggest that LPS requires TLR4 and TRIF, but not MyD88, to inhibit NLRP1-mediated cell death. To further confirm the involvement of TRIF, we also tested another TLR ligand that signals through TRIF, polyinosinic-polycytidylic acid (poly(I:C), a synthetic analog of double-stranded RNA (Takeuchi and Akira, 2010)). Treatment with poly(I:C) also significantly inhibited NLRP1-induced cell death in VBP-treated BMDMs (Fig. 2C,D). Together, these data suggest that TLR–TRIF signaling regulates NLRP1-mediated PANoptosis.

### IFN-β production driven by IRF3 inhibits NLRP1-mediated PANoptosis

**Figure 3:**
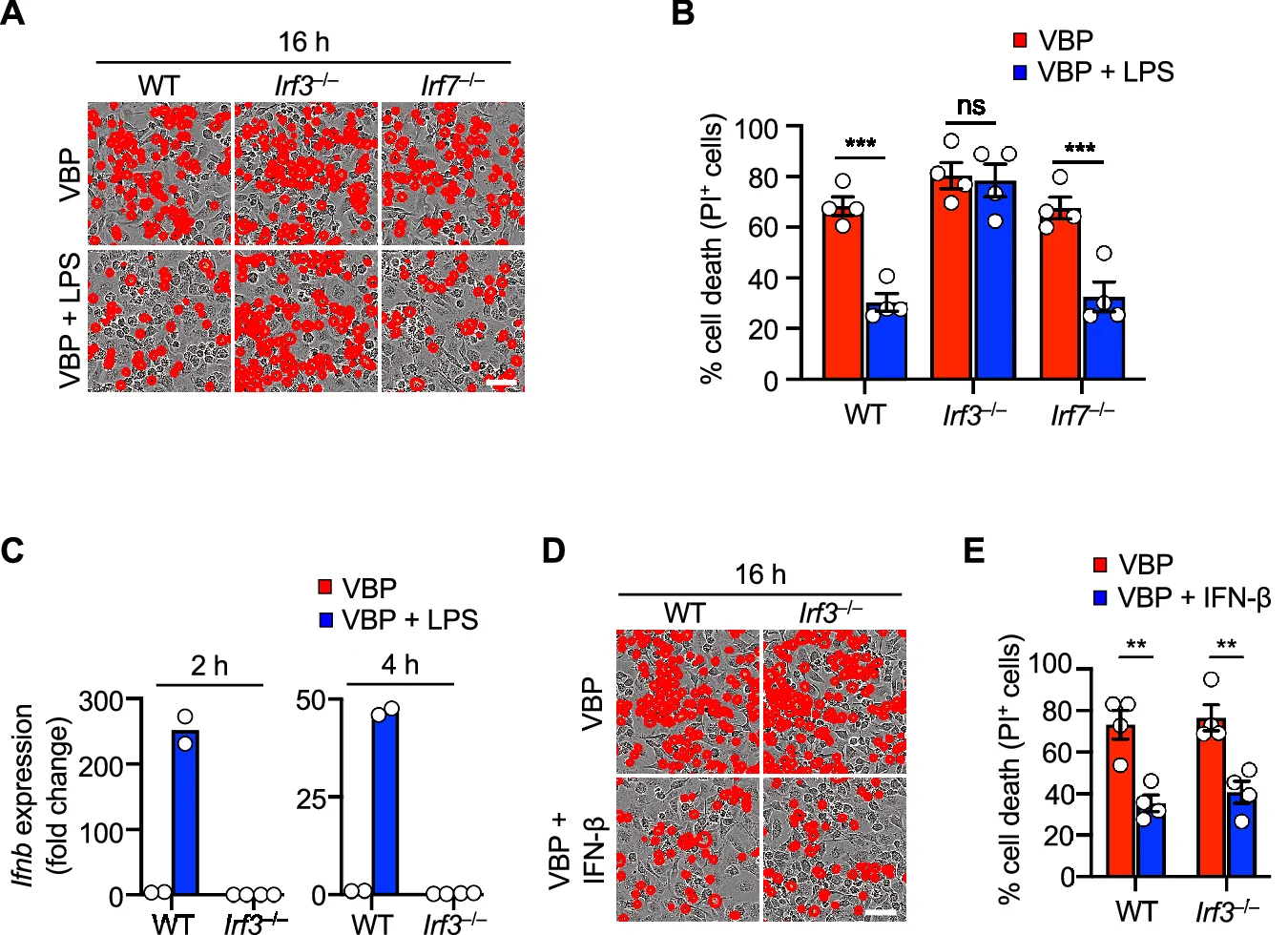
IFN-β production driven by IRF3 inhibits NLRP1-mediated cell death. (**A**,** B**) Representative images (**A**) and quantification (**B**) of cell death in wild type (WT), *Irf3*^*–/–*^, and *Irf7*^*–/–*^ mouse bone marrow-derived macrophages (BMDMs) at 16 h after treatment with Val-boroPro (VBP) or VBP plus lipopolysaccharide (LPS). (**C**) *Ifnb* gene expression in WT and *Irf3*^*–/–*^ BMDMs at 2 h and 4 h after treatment with VBP or VBP plus LPS, expressed in fold change relative to the media control for each genotype, using *Actb* as the housekeeping gene. (**D**,** E**) Representative images (**D**) and quantification (**E**) of cell death in WT and *Irf3*^*–/–*^ BMDMs at 16 h after treatment with VBP or VBP plus IFN-β. (**A**–**E**) Data were representative of three independent biological replicates, *n* = 4 (**B**,** E**) or 2 (**C**) technical replicates. Scale bar: 50 μm (**A**,** D**). (**B**) WT VBP vs VBP + LPS, ***P = 0.0001; *Irf3*^*–/–*^ VBP vs VBP + LPS, *P* = 0.9895 (ns); *Irf7*^*–/–*^ VBP vs VBP + LPS, ***P = 0.0003. (**E**) WT VBP vs VBP + IFN-β, **P = 0.0011; *Irf3*^*–/–*^ VBP vs VBP + IFN-β, **P = 0.0017. Two-way ANOVA with Sidak’s multiple comparison test was used (**B**, **E**). The data were represented as mean ± SEM in (**B**, **E**) and as mean in (**C**). Source data are available online for this figure.

Given the critical role we observed for the TLR–TRIF signaling axis in regulating NLRP1-mediated cell death, we sought to identify additional regulators in this process. TRIF signaling facilitates the activation of multiple interferon regulatory factors (IRFs), including IRF3 and IRF7, to modulate inflammatory responses during pathogenic infections (Au et al, 1995; Barnes et al, 2004; Honda and Taniguchi, 2006; Kawai et al, 2024; Sato et al, 2000; Schafer et al, 1998; Wang et al, 2024). Therefore, we assessed the role of these IRFs in regulating NLRP1-mediated cell death. We found that robust cell death occurred in WT, *Irf3*^–/–^, and *Irf7*^–/–^ BMDMs in response to VBP (Fig. 3A,B). The addition of LPS significantly inhibited VBP-induced cell death in *Irf7*^–/–^ BMDMs, but not in *Irf3*^–/–^ BMDMs (Fig. 3A,B), suggesting that LPS requires IRF3 to inhibit NLRP1-mediated cell death.

**Figure EV4:**
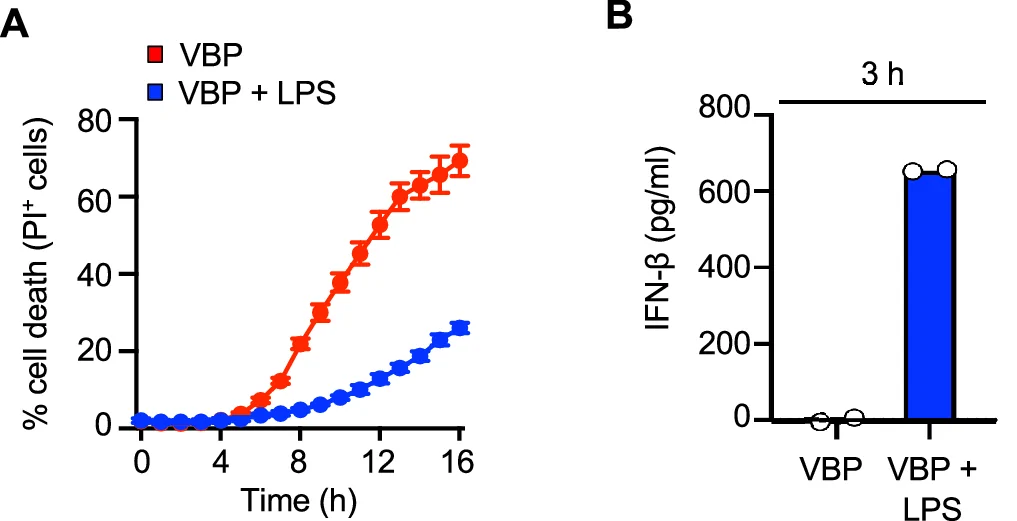
LPS induces IFN-β production prior to the onset of cell death. (**A**) Cell death kinetics in wild-type (WT) mouse bone marrow-derived macrophages (BMDMs) following treatment with Val-boroPro (VBP) or VBP plus lipopolysaccharide (LPS). (**B**) Quantification of IFN-β in the supernatant of WT BMDMs at 3 h after treatment with VBP or VBP + LPS. (**A**,** B**) Data were representative of three independent biological replicates. *n* = 4 (**A**) or 2 (**B**) technical replicates. The data were represented as mean ± SEM in (**A**) and mean in (**B**). Source data are available online for this figure.

IRF3 activation downstream of TRIF can drive the production of IFN-β (Han et al, 2004, Yamamoto et al, 2003). To determine whether IRF3-mediated IFN-β production is required to block VBP-induced cell death, we first assessed the relative timing of IFN-β production and cell death in response to VBP and VBP plus LPS. We observed that VBP-stimulated BMDMs began undergoing cell death around 6 h after stimulation, and the addition of LPS along with VBP provided protection (Fig. EV4A). Meanwhile, BMDMs stimulated with VBP plus LPS (but not VBP alone) produced IFN-β by 3 h after stimulation (Fig. EV4B), suggesting that the IFN-β production occurred before the cell death began. To further investigate the production of IFN-β downstream of the TLR–TRIF–IRF3 axis, we measured *Ifnb* gene expression. In WT BMDMs, treatment with VBP alone had no effect on *Ifnb* expression, whereas treatment with VBP plus LPS significantly upregulated *Ifnb* expression (Fig. 3C). This induction was completely abrogated in *Irf3*^–/–^ BMDMs at both 2 and 4 h after stimulation (Fig. 3C). Together, these data suggest that LPS rapidly induces the production of IFN-β through the TLR–TRIF–IRF3 signaling axis, allowing IFN-β to inhibit VBP-induced cell death.

To further confirm this hypothesis, we treated WT and *Irf3*^–/–^ BMDMs with exogenous IFN-β and found that VBP-mediated cell death was reduced by IFN-β treatment in both genotypes (Fig. 3D,E). Overall, these data suggest that TLR–TRIF-IRF3-mediated IFN-β production inhibits NLRP1-mediated cell death.

### Type I IFN signaling inhibits NLRP1-mediated PANoptosis in human and murine macrophages and monocytes

**Figure 4:**
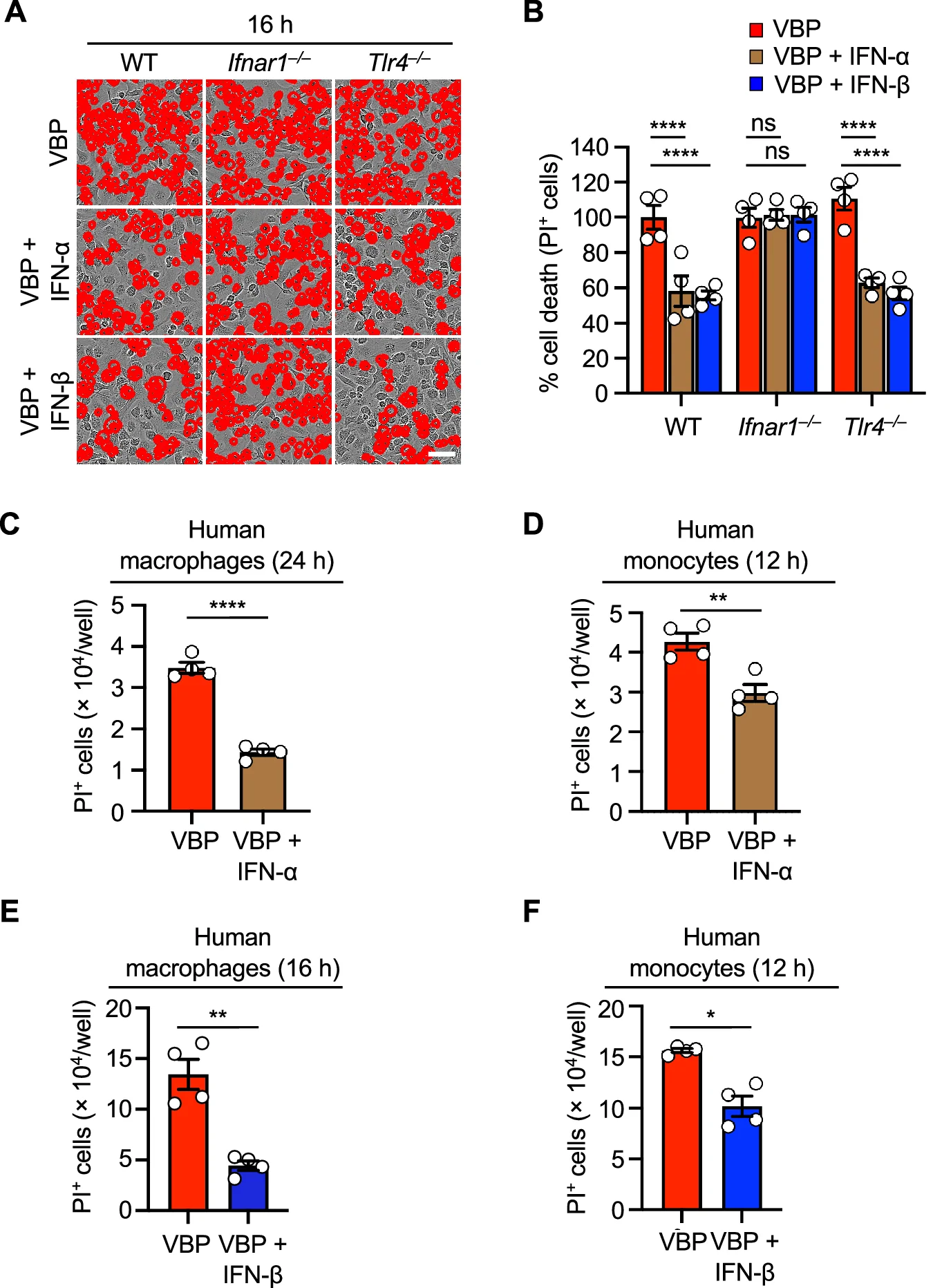
Type I IFN signaling inhibits NLRP1-mediated PANoptosis in human and murine macrophages and human monocytes. (**A**,** B**) Representative images (**A**) and quantification (**B**) of cell death in wild type (WT), *Ifnar1*^*–/–*^, and *Tlr4*^*–/–*^ mouse bone marrow-derived macrophages (BMDMs) at 16 h after treatment with Val-boroPro (VBP), VBP plus IFN-α, or VBP plus IFN-β. (**C**,** D**) Quantification of cell death in human macrophages at 24 h after treatment with VBP or VBP plus IFN-α (**C**), and human monocytes at 12 h after treatment with VBP or VBP plus IFN-α (**D**). (**E**,** F**) Quantification of cell death in human macrophages at 16 h after treatment with VBP or VBP plus IFN-β (**E**), and human monocytes at 12 h after treatment with VBP or VBP plus IFN-β (**F**). Donors in (**E**, **F**) are distinct from donors in (**C**, **D**). (**A**–**F**) Data were representative of three independent biological replicates, (**B**–**F**) *n* = 4 technical replicates. Scale bar: 50 μm (**A**). (**B**) WT VBP vs VBP + IFN-α, ****P < 0.0001; WT VBP vs VBP + IFN-β, ****P < 0.0001; *Ifnar1*^*–/–*^ VBP vs VBP + IFN-α, *P* > 0.9999 (ns); *Ifnar1*^*–/–*^ VBP vs VBP + IFN-β, *P* > 0.9999 (ns); *Tlr4*^*–/–*^ VBP vs VBP + IFN-α, ****P < 0.0001; *Tlr4*^*–/–*^ VBP vs VBP + IFN-β, ****P < 0.0001. (**C**) ****P < 0.0001. (**D**) **P = 0.0050. (**E**) **P = 0.0061. (**F**) *P = 0.0108. Two-way ANOVA with Sidak’s multiple comparison test (**B**) and Welch’s *t*-test (**C**–**F**) were used. The data were represented as mean ± SEM in (**B**–**F**). Source data are available online for this figure.

Our observations suggest that LPS induces IFN-β production to inhibit NLRP1-mediated cell death. To assess the broader potential of type I IFNs to block NLRP1-mediated cell death, we tested the effects of IFN-α and IFN-β on VBP-mediated cell death. The addition of IFN-α or IFN-β inhibited VBP-induced cell death in both WT and *Tlr4*^–/–^ BMDMs (Fig. 4A,B), suggesting that the need for TLR4 to mediate LPS-driven inhibition was bypassed by direct treatment with type I IFNs. Moreover, we found that IFN-α- or IFN-β-mediated inhibition of NLRP1-induced cell death required IFN receptor subunit 1 (IFNAR1) (Fig. 4A,B), the receptor essential for type I IFN-mediated immune responses (McNab et al, 2015; Novick et al, 1994).

**Figure EV5:**
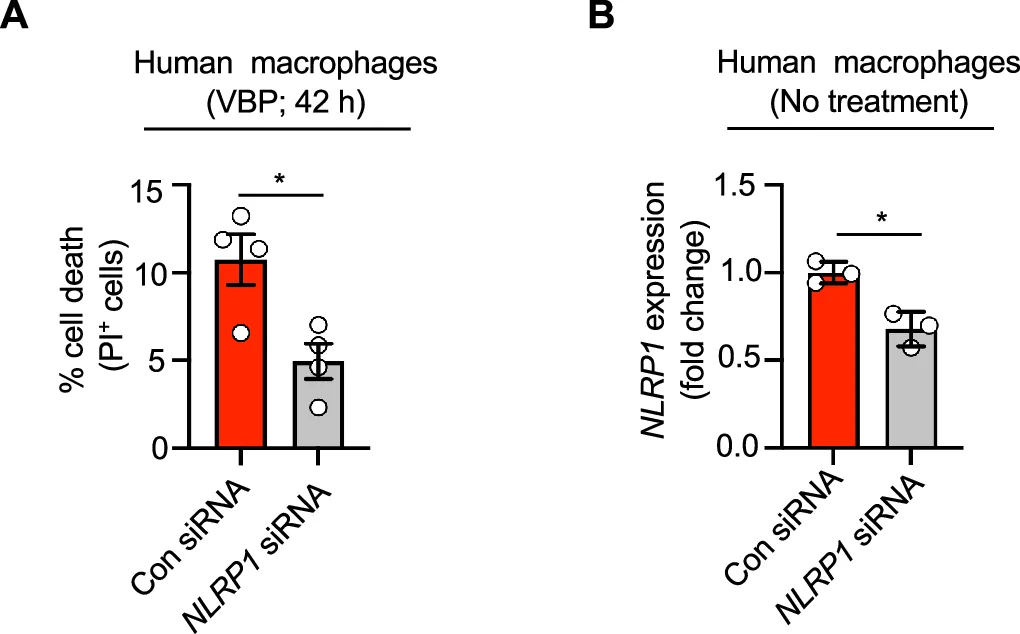
NLRP1 drives VBP-induced cell death in human macrophages. (**A**) Quantification of cell death in the indicated human macrophages at 42 h after treatment with Val-boroPro (VBP). (**B**) *NLRP1* gene expression after silencing with control siRNA (Con siRNA) or *NLRP1* siRNA. qRT-PCR data were expressed as fold change relative to the siRNA control, using *18S* as the housekeeping gene. (**A**,** B**) Data were representative of three independent biological replicates. *n* = 4 (left) or 3 (right) technical replicates. (**A**) Con siRNA vs *NLRP1* siRNA, *P = 0.0197. (**B**) Con siRNA vs *NLRP1* siRNA, *P = 0.0136 (Welch’s *t*-test). The data were represented as mean ± SEM. Source data are available online for this figure.

To complement our findings in mouse BMDMs, we assessed primary human macrophages and monocytes. We observed that VBP-induced cell death was dependent on NLRP1 (Fig. EV5) and was significantly inhibited by treatment with exogenous IFN-α (Fig. 4C,D) or IFN-β (Fig. 4E,F) in primary human macrophages and monocytes. Hence, type I IFNs are key inhibitors of NLRP1-mediated PANoptosis.

### LPS regulates NLRP1 expression via type I IFNs in human and murine macrophages and human monocytes

**Figure 5:**
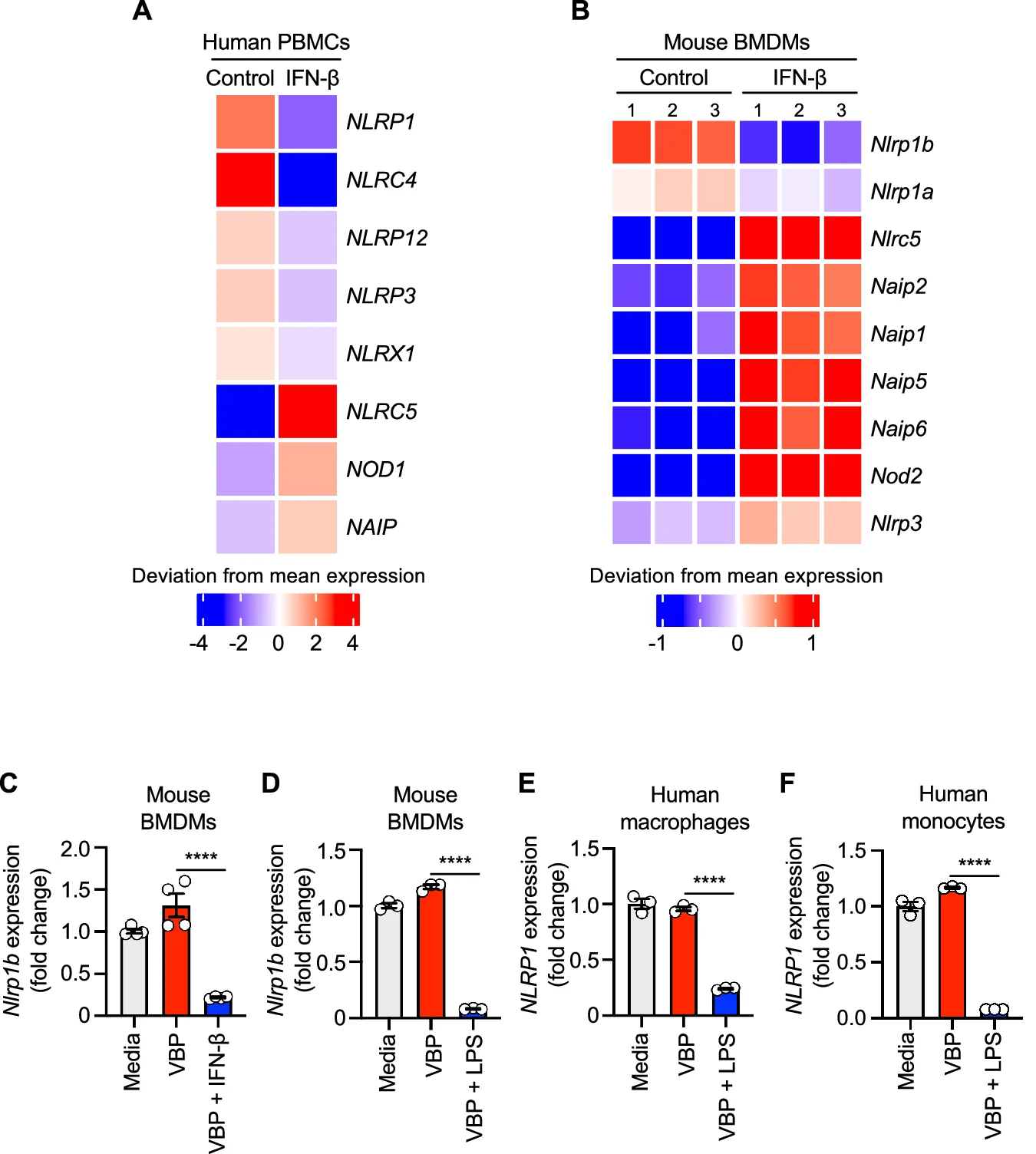
LPS regulates NLRP1 expression via type I IFNs in human and murine macrophages and monocytes. (**A**) Heatmap depicting NLR family gene expression from a single-cell RNA-Seq analysis of peripheral blood mononuclear cells (PBMCs) extracted from patients with systemic lupus erythematosus and treated with IFN-β as compared with untreated control PBMCs from the same patient set (GSE96583). (**B**) Heatmap depicting NLR family gene expression from an RNA-Seq analysis of mouse bone marrow-derived macrophages (BMDMs) treated with IFN-β as compared with untreated controls (GSE232827). (**C**,** D**) Expression of *Nlrp1b* in mouse BMDMs at 6 h after treatment with media, Val-boroPro (VBP), or VBP plus either IFN-β (**C**) or LPS (**D**). (**E**,** F**) Expression of *NLRP1* in primary human macrophages at 6 h (**E**) and primary human monocytes at 4 h (**F**) after treatment with media, VBP, or VBP plus LPS. (**C**–**F**) Data were expressed as fold change relative to media controls, using *18s* or *18S* as the housekeeping gene. Data are representative of three independent biological replicates, *n* = 2 technical replicates combined from two biological replicates (**C**) or *n* = 3 technical replicates from one biological replicate (**D**–**F**). ****P < 0.0001. One-way ANOVA with Dunnett’s multiple comparison test (**C**–**F**) was used. The data were represented as mean ± SEM in (**C**–**F**). Source data are available online for this figure.

Given the crucial role of IFNs in modulating the expression of innate immune receptors, we analyzed publicly available datasets to understand the impact of IFN treatment on NLR gene expression (Kang et al, 2018; Kotov et al, 2023). We found that *NLRP1* was significantly downregulated in response to IFN-β in human PBMCs (Fig. 5A), as were its orthologs in mouse BMDMs (Fig. 5B), suggesting that type I IFN signaling modulates the expression of *NLRP1* to regulate NLRP1-driven PANoptosis. Moreover, among the other NLRs analyzed in the human dataset, only *NLRC4* was markedly downregulated; in the mouse dataset, all NLRs besides *Nlrp1a* and *Nlrp1b* were upregulated. These results suggest that, among NLRs, the IFN-β–mediated regulation is specific to NLRP1. We then validated this finding using qRT-PCR; the expression of *Nlrp1b* was significantly reduced in IFN-β– or LPS-treated mouse BMDMs (Fig. 5C,D), and *NLRP1* expression was also significantly reduced in LPS-treated primary human macrophages and monocytes (Fig. 5E,F).

Altogether, these data show that type I IFN signaling inhibits NLRP1-mediated cell death by blocking *NLRP1* gene expression, suggesting IFNs as potential therapeutics to block NLRP1-mediated PANoptosis and mitigate inflammation and disease.

## Conclusion

The timely activation and resolution of innate immune signaling are critical for mitigating pathogenic infections; however, aberrant activation can be harmful and lead to chronic inflammatory diseases (Zhong et al, 2016). NLRP1 has been implicated in several autoinflammatory and autoimmune disorders, including vitiligo, psoriasis, multiple sclerosis, and rheumatoid arthritis (Dieude et al, 2011; Fenini et al, 2022; Grandemange et al, 2015; Jin et al, 2010; Sui et al, 2012; Verma et al, 2012; Zhong et al, 2016; Zurawek et al, 2010). In particular, mutations that cause aberrant activation of NLRP1 can underlie disease. In patients with multiple sclerosis, *NLRP1* mutations were identified together with elevated levels of proinflammatory IL-1β (Maver et al, 2017). Similarly, variants of *NLRP1* in patients with rheumatoid arthritis are associated with elevated caspase-1 levels and constitutive IL-1β production, suggesting excessive activation of NLRP1 (Grandemange et al, 2015). Furthermore, a non-synonymous mutation in *NLRP1* has been correlated with elevated systemic levels of caspase-1 and IL-18 in patients with autoimmune disorders (Grandemange et al, 2017), further supporting the role of aberrant NLRP1 activation in driving inflammation and disease progression. Given these clinical connections, and the lack of approved treatments to specifically target NLRP1 activation, understanding the regulation of NLRP1 is essential for developing novel therapies.

Here, we found that LPS inhibited NLRP1-mediated PANoptosis and inflammatory cytokine release, which contrasts with the well-established role of LPS as an activator of innate immune signaling. This inhibition occurred through TLR4–TRIF signaling to activate IRF3 and subsequent IFN production. Furthermore, type I IFN treatment replicated the protective effects of LPS in inhibiting NLRP1-mediated PANoptosis, even in cells lacking TLR4 or IRF3, where LPS was not protective.

The role of LPS in suppressing NLRP1-driven innate immune responses differs from its classical function as an activator of innate immunity. Our findings regarding the role of IFN-β explain this paradox. It has long been known that LPS can induce IFN-β in macrophages (Gessani et al, 1989). Our findings suggest that IFN-β increases the expression of the genes encoding several NLRs, but decreases the expression of *NLRP1*. Hence, the similar effects of LPS and IFN-β highlight a non-canonical, indirect function of LPS in suppressing innate immune sensor transcription in NLRP1-mediated contexts. IFN signaling regulates the expression of several innate immune genes required for cell death (Ivashkiv and Donlin, 2014; Man et al, 2015; McNab et al, 2015; Platanias, 2005), and our analyses suggest that IFN signaling likewise suppresses the expression or pre-mRNA processing (Lee and Alper, 2022; Ueda et al, 2024) of *NLRP1*. Future studies will be useful to determine the specific mechanism used by IFNs to inhibit NLRP1.

The regulatory pathway we identified here may provide the mechanistic explanation for the therapeutic promise of IFN-β in several conditions that have been linked to *NLRP1* mutations, such as autoinflammatory and autoimmune diseases, including multiple sclerosis and rheumatoid arthritis (Grandemange et al, 2017; Jiang et al, 2020; Maver et al, 2017; Owens et al, 2014; Sui et al, 2012), as well as CNS diseases such as Alzheimer’s (Pontillo et al, 2012; Yap et al, 2019). Furthermore, our findings suggest that type I IFN might therapeutically modulate inflammation and cell death to mitigate other NLRP1-mediated diseases, reducing disease burden and improving patient outcomes.

## Methods

**Table:** Reagents and tools table

Reagent/resource	Reference or source	Identifier or catalog number
**Experimental models**		
BMDMs (bone marrow-derived macrophages)	Generated in this study	N/A
C57BL/6 mice	Jackson Lab	000664
*Nlrp1b*^*–/–*^ mice	(Kovarova et al, 2012)	N/A
*Casp1*^*–/–*^ mice	(Man et al, 2016)	N/A
*Casp1*^*–/–*^*Casp8*^*–/–*^*Ripk3*^*–/–*^ mice	(Sundaram et al, 2023)	N/A
*Tlr4*^*–/–*^ mice	(Hoshino et al, 1999)	N/A
*MyD88*^*–/–*^ mice	(Adachi et al, 1998)	N/A
*Trif*^*–/–*^ mice	(Yamamoto et al, 2003)	N/A
*Irf3*^–/–^ mice	(Sato et al, 2000)	N/A
*Irf7*^–/–^ mice	(Honda et al, 2005)	N/A
*Nlrp3*^*–/–*^ mice	(Kanneganti et al, 2006)	N/A
*Ifnar1*^*–/–*^ mice	(Muller et al, 1994),	N/A
*Casp11*^*–/–*^ mice	(Kayagaki et al, 2011)	N/A
Human peripheral blood from healthy donors	This paper	N/A
**Recombinant DNA**		
N/A		
**Antibodies**		
Anti-caspase-1	Adipogen	AG-20B-0042
Anti-GSDMD	Abcam	ab209845
Anti-caspase-3	Cell Signaling Technology	9662
Anti-cleaved caspase-3	Cell Signaling Technology	9661
Anti-caspase-7	Cell Signaling Technology	9492
Anti-cleaved caspase-7	Cell Signaling Technology	9491
Anti-caspase-8	Cell Signaling Technology	4927
Anti-cleaved caspase-8	Cell Signaling Technology	8592
Anti-HMGB1	Abcam	18256
Anti-LDHA (LDH)	Proteintech	19987-1-AP
Anti-GAPDH	CST	5174
HRP-conjugated secondary anti-rabbit antibody (H + L)	Jackson ImmunoResearch Laboratories	111-035-047
HRP-conjugated secondary anti-mouse antibody (H + L)	Jackson ImmunoResearch Laboratories	315-035-047
**Oligonucleotides and other sequence-based reagents**		
siGENOME SMARTpool siRNA for human *NLRP1*	Horizon Discovery	M-004423-03-0005
siGENOME SMARTpool non-targeting control siRNA	Horizon Discovery	D-001206-14-20
TaqMan probe for mouse *Nlrp1b*	Applied Biosystems	Assay ID: Mm01241387_m1
TaqMan probe for human *NLRP1*	Applied Biosystems	Assay ID: Hs00248187_m1
Eukaryotic *18S* rRNA Endogenous Control	Applied Biosystems	4319413E
*Ifnb* forward primer: 5’-GCCTTTGCCATCCAAGAGATGC-3’	Integrated DNA Technologies	Custom
*Ifnb* reverse primer: 5’-ACACTGTCTGCTGGTGGAGTTC-3’	Integrated DNA Technologies	Custom
*Actb* forward primer: 5’-GGCTGTATTCCCCTCCATCG-3’	Integrated DNA Technologies	Custom
*Actb* reverse primer: 5’-CCAGTTGGTAACAATGCCATGT-3’	Integrated DNA Technologies	Custom
**Chemicals, enzymes and other reagents**	**Reference or Source**	
IMDM	Thermo Fisher Scientific	12440053
DMEM	Gibco	11995-065
RPMI	Corning	10-040-CV
Heat-inactivated FBS	Biowest	S1620
Non-essential amino acids	Thermo Fisher Scientific	11140-050
Penicillin-streptomycin	Thermo Fisher Scientific	15070-063
Lymphoprep solution	Stemcell Technologies	07801/07811
EasySep Direct Human Monocyte Isolation Kit	Stemcell Technologies	19669
Human MCSF	Peproptech	300-25
Val-boroPro	Millipore Sigma	5.31465
LPS	Millipore Sigma	L2630
Recombinant mouse IFN-β	PBL Assay	12400-1
Recombinant mouse IFN-α	BioLegend	752806
Recombinant human IFN-β	Peprotech	300-02bc
Recombinant human IFN-α 2a	PBL Assay	11100-1
Poly(I:C)	InvivoGen	tlrl-pic
1G244	Focus Biomolecules	847928-32-9
Ads032	MedChemExpress	HY-156798
Neon Transfection System 100 μl Kit	Thermo Fisher Scientific	MPK10025
PureLink RNA Mini Kit	Invitrogen	12183018 A
Propidium iodide	Invitrogen	P3566
TaqMan Gene Expression Assay	Applied Biosystems	4331182
Fast TaqMan 2× Master Mix	Applied Biosystems	4444557
SYBR Green 2× Master Mix	Applied Biosystems	4367659
High-capacity cDNA Reverse Transcription Kit	Thermo Fisher Scientific	4374966
Legend Max Mouse IFN-β ELISA Kit	BioLegend	439407
Mouse IL-18 ELISA Kit	Invitrogen	BMS618-3
Immobilon Forte Western HRP Substrate	Millipore	WBLUF0500
SuperSignal West Femto Maximum Sensitivity Substrate	Thermo Fisher Scientific	34096
**Software**		
Fiji	ImageJ	https://imagej.net/ij/
GraphPad Prism v8.0 and later	GraphPad	N/A
STAR (version 2.7.11b)	N/A	Dobin et al, 2013
DESeq2 (version 1.46.0)	N/A	Love et al, 2014
ComplexHeatmap R package (2.22.0)	N/A	Gu et al, 2016
R (version 4.4.2)	N/A	
Seurat (version 5.2.1)	N/A	Hao et al, 2024
**Other**		
IncuCyte S3	Sartorius	N/A
Amersham Imager	GE Healthcare	N/A
7500 Fast Real-time PCR System	Applied Biosystems	N/A
NanoDrop 2000	Thermo Fisher Scientific	N/A
Neon Transfection System	Thermo Fisher Scientific	MPS100

### Ethical statement

For studies involving human cells, fresh human blood was collected from the apheresis rings of anonymous, healthy blood donors from the blood bank at St. Jude Children’s Research Hospital, through approval of the St. Jude Institutional Review Board (IRB No. Pro00006009), in protocols consistent with the Declaration of Helsinki and the US Department of Health and Human Services Belmont Report. Donors signed a standard informed consent document that explains the apheresis process and important details prior to donating. Animal studies were conducted under protocols approved by the St. Jude Children’s Research Hospital Committee on the Use and Care of Animals (Protocol No. 482).

### Mice

Wild-type (WT) C57/BL6, *Nlrp1b*^*–/–*^ (Kovarova et al, 2012), *Casp1*^*–/–*^ (Man et al, 2016), *Casp1*^*–/–*^*Casp8*^*–/–*^*Ripk3*^*–/–*^ (Sundaram et al, 2023), *Tlr4*^*–/–*^ (Hoshino et al, 1999), *MyD88*^*–/–*^ (Adachi et al, 1998), *Trif*^*–/–*^ (Yamamoto et al, 2003), *Irf3*^–/–^ (Sato et al, 2000), *Irf7*^–/–^ (Honda et al, 2005), *Nlrp3*^*–/–*^ (Kanneganti et al, 2006), *Ifnar1*^*–/–*^ (Muller et al, 1994), and *Casp11*^*–/–*^ (Kayagaki et al, 2011) mice used in this study have been described previously. All mice were generated on or extensively backcrossed to the C57/BL6 background. Mice were bred at the Animal Resources Center at St. Jude Children’s Research Hospital and maintained under specific pathogen-free conditions. Mice were maintained on a 12 h light/dark cycle and were fed standard chow. Age- and sex-matched 6- to 10-week-old mice were used in this study.

### Bone marrow-derived macrophages

Primary mouse bone marrow-derived macrophages (BMDMs) from WT and indicated mutant mice were grown for 6 days in IMDM (12440053, Thermo Fisher Scientific) supplemented with 10% heat-inactivated fetal bovine serum (HI-FBS; S1620, Biowest), 30% L929-conditioned media, 1% non-essential amino acids (11140-050, Thermo Fisher Scientific), and 1% penicillin and streptomycin (15070-063, Thermo Fisher Scientific). BMDMs at a density of 1 × 10^6^ cells/well in 12-well plates, 5 × 10^5^ cells/well in 24-well plates, or 2.5 × 10^5^ cells/well in 48-well plates were seeded into growth media and incubated overnight before use.

### Isolation of monocytes from human blood and differentiation to monocyte-derived macrophages

Fresh human blood was collected from the apheresis rings of anonymous healthy blood donors from the blood bank at St. Jude Children’s Research Hospital. Peripheral blood mononuclear cells (PBMCs) were isolated from the freshly collected blood using Lymphoprep solution (07801/07811, Stemcell Technologies). Untouched naïve monocytes were purified from PBMCs using the EasySep Direct Human Monocyte Isolation Kit (19669, Stemcell Technologies) strictly in accordance with the manufacturer’s protocol. The purified monocytes were either used directly in experiments or were first further differentiated into monocyte-derived macrophages by culturing in RPMI media (10-040-CV, Corning) supplemented with 10% HI-FBS, 1% penicillin-streptomycin, and 20 ng/ml human MCSF (300-25, Peprotech) for 6 days. After 3 days, an additional 10 ml of the same media was added to the cells. On day 6, total cells were harvested and washed three times with PBS. The total cells were resuspended in RPMI supplemented with 10% HI-FBS, 1% penicillin-streptomycin, and 20 ng/ml human MCSF and seeded for experiments at the same densities listed for BMDMs above or at 2.0 × 10^5^ cells/well in 96-well plates.

### Cell stimulation

Primary mouse BMDMs were stimulated in DMEM (11995-065, Gibco), and primary human monocytes and macrophages were stimulated in RPMI, both containing 10% HI-FBS and 1% penicillin and streptomycin. Cells were then stimulated with 10 μM VBP (5.31465, Millipore Sigma), alone or together with 100 ng/ml LPS (L2630, Millipore Sigma), 100 ng/ml IFN-β (mouse: 12400-1, PBL Assay; human: 300-02bc, Peprotech), or 100 ng/ml IFN-α (mouse: 752806, BioLegend; human: 11100-1, PBL Assay), for 16 h unless otherwise indicated. In some experiments, cells were primed with LPS for 4 h prior to VBP stimulation, as indicated in the figure legend. In addition, the following ligands or inhibitors alone or in combinations were added where indicated: 5 μg/ml poly(I:C) (tlrl-pic, InvivoGen), 20 μM 1G244 (847928-32-9, Focus Biomolecules), and 175 μM Ads032 (HY-156798, MedChemExpress). Cells were stimulated with the indicated ligands or inhibitors in 500 μL (12-well plates), 350 μL (24-well), 250 μL (48-well), or 150 μL (96-well) medium per well, respectively. No randomization or blinding was performed; allocation was based on the pre-determined experimental layout.

### siRNA knockdown in human macrophages

For the knockdown reactions in fully differentiated monocyte-derived macrophages, nucleofection with the Neon Transfection System 100 μl Kit (MPK10025, Thermo Fisher Scientific) was used to transfect siRNA in the Neon Transfection System (MPS100, Thermo Fisher Scientific). A total of 1 × 10^6^ cells were transfected with 1 μl of 50 μM siGENOME SMARTpool control siRNA (D-001206-14-20, Horizon Discovery) or human *NLRP1* siRNA (M-004423-03-0005, Horizon Discovery) with 1500 V and one 20 mS pulse. The cells were then transferred to 1 ml of warm complete RPMI and seeded in 12 or 24-well tissue culture plates. At 2 h after transfection, each well was supplemented with an equal volume of warm RPMI medium. The medium was replaced at 24 h after transfection, and the cells were rested for another 24 h before experimental stimulations.

### Real-time imaging for cell death

The kinetics of cell death were determined using the IncuCyte S3 (Sartorius) live-cell imaging system. Cells were treated with respective triggers and stained with propidium iodide (PI; P3566, Invitrogen) following the manufacturer’s protocol. The plate was scanned, and fluorescent and phase-contrast images (4 image fields/well) were acquired in real-time. PI-positive dead cells are marked with a red mask for visualization. The image analysis, masking, and quantification of dead cells were performed using the software package supplied with the IncuCyte imager, as previously described (Tweedell et al, 2024).

### Immunoblot analysis

For caspase blots, BMDMs were lysed along with the supernatant using 50 μL caspase lysis buffer (containing 1× protease inhibitors, 1× phosphatase inhibitors, 10% NP-40, and 25 mM DTT) and 100 μL 4× SDS loading buffer. For signaling blots, supernatants were removed, and cells were washed once with PBS at the indicated timepoints, followed by cell lysis with RIPA buffer. For immunoblot analysis of LDH and HMGB1, cell supernatant was combined with sample loading buffer (containing SDS and 2-mercaptoethanol) at a ratio of 1:1. Proteins were separated by electrophoresis through 10–12% polyacrylamide gels. PVDF membranes were used to transfer the resolved proteins, and the blots were blocked with 5% skim milk for 1 h at room temperature. Blots were incubated with primary antibodies at 4 °C overnight, followed by incubation with secondary horseradish peroxidase (HRP) antibodies for 1 h at room temperature. The GE Amersham Imager 600 was used to image the immunoblots, using Immobilon Forte Western HRP Substrate (WBLUF0500, Millipore) or SuperSignal West Femto Maximum Sensitivity Substrate (34096, Thermo Fisher Scientific). Western blot images were processed using Fiji (ImageJ). The following antibodies were used: anti-caspase-1 (AG-20B-0042, AdipoGen, 1:2000), anti-GSDMD (ab209845, Abcam, 1:1000), anti-caspase-3 (#9662, Cell Signaling Technology [CST], 1:1000), anti-cleaved caspase-3 (#9661, CST, 1:1000), anti–caspase-7 (#9492, CST, 1:1000), anti-cleaved caspase-7 (#9491, CST, 1:1000), anti–caspase-8 (#4927, CST, 1:1000), anti-cleaved caspase-8 (#8592, CST, 1:1000), anti-HMGB1 (Abcam, #18256, 1:1000), anti-LDHA (LDH; #19987-1-AP, Proteintech, 1:1000), anti-GAPDH (#5174, CST, 1:5000), or HRP-conjugated secondary antibodies (111-035-047 (anti-rabbit) and 315-035-047 (anti-mouse), Jackson ImmunoResearch Laboratories, 1:5000).

### Quantitative reverse-transcriptase PCR (qRT-PCR) analysis

Cells were lysed in lysis buffer, and total RNA was isolated with the PureLink RNA Mini Kit (12183018A, Invitrogen) according to the manufacturer’s instructions. RNA was quantified with a NanoDrop 2000 (Thermo Fisher Scientific). Then, 1 µg of total RNA was reverse-transcribed with the High-Capacity cDNA Reverse Transcription Kit (4374966, Thermo Fisher Scientific). The amplified cDNA was diluted to 10 ng/µl.

TaqMan-based qRT-PCR was performed using 40 ng of cDNA and TaqMan probes for mouse *Nlrp1b* (Assay ID: Mm01241387_m1) and human *NLRP1* (Assay ID: Hs00248187_m1) using a TaqMan Gene Expression Assay (4331182, Applied Biosystems) with Fast TaqMan 2× Master Mix (4444557, Applied Biosystems) on a 7500 Fast Real-time PCR System (Applied Biosystems). The delta-delta cycle threshold method was used to normalize expression to the reference gene *18S* (Eukaryotic *18S* rRNA Endogenous Control; 4319413E, Applied Biosystems).

SYBR Green-based qRT-PCR was performed using 40 ng of cDNA on a 7500 Fast Real-time PCR System, using SYBR Green 2× Master mix (4367659, Applied Biosystems) and the appropriate primers (purchased from Integrated DNA Technologies; Reagents and Tools Table). The delta-delta cycle threshold method was used to normalize expression to the reference gene *Actb*.

### Enzyme-linked immunosorbent assay (ELISA)

Supernatant was used to measure IL-18 with a Mouse IL-18 ELISA Kit (BMS618-3, Invitrogen) and IFN-β with a Legend Max Mouse IFN-β ELISA Kit (439407, BioLegend) according to the manufacturers’ instructions.

### RNA-Seq analysis

Raw RNA-Seq files from untreated (controls) and IFN-β–treated BMDMs (Kotov et al, 2023) were downloaded from GEO (GSE232827). Raw files were mapped to GENCODE Mouse Assembly Release M36 (GRCm39) (Mudge et al, 2025) using STAR v2.7.11b (Dobin et al, 2013). The “--twopassMode” was set to Basic, and “--quantMode” was set to GeneCounts for accurate gene counting. The primary assembly comprehensive gene annotation from GENCODE (M36) was used as an input for the “--sjdbGTFfile” option in STAR. The columns corresponding to unstranded libraries in the gene count outputs of STARs were merged. Differential gene expression was performed using DESeq2 (Love et al, 2014). Any gene with a row sum <10 across all the libraries was excluded from the downstream analysis. The DESeqDataSet was created using the DESeqDataSetFromMatrix function, with only a single experimental factor (treatment condition) included in the model option. Genes that demonstrated a log-fold change above 1 or below −1 with an adjusted *P* value below 0.05 in DESeq2 output were considered differentially expressed. The raw counts were normalized using the “rlog” option of DESeq2. The heatmaps were generated using the ComplexHeatmap R package (Gu et al, 2016).

### Single-cell RNA-Seq analysis

Raw count matrices were downloaded from two single-cell RNA-Seq libraries derived from human PBMCs collected from patients with systemic lupus erythematosus (SLE) (Kang et al, 2018) (GSE96583). The chosen entries were “batch 2 control” and “batch 2 stim (IFN-beta)”, which represented PBMCs gathered from eight patients with SLE, separated into two aliquots for each individual. One aliquot was stimulated (“batch 2 stim (IFN-beta)” - pooled), while the other served as a control (“batch 2 control” - pooled). The raw matrices were uploaded to Seurat (Hao et al, 2024) using the “Read10X” function. An object was created for each library, and the two objects were merged using the “Merge” and “JoinLayers” functions. Cells that met any of the following criteria were excluded from subsequent analysis: (1) mitochondrial content above 50%, (2) UMI count below 1000, or (3) number of detected genes below 250 or above 2500. The “subset” function was applied to perform the QC filtering. The merged object was then normalized and scaled using Seurat’s “NormalizeData” and “ScaleData” functions. Differential Gene Expression to compare IFN-β–stimulated cells with control cells was then performed using the “FindMarkers” function of Seurat with the logistic regression method to account for lowly expressed genes. Next, the single cells were pseudo-bulked using the “AggregateExpression” module, and the “group.by” option was set to “orig.ident” to create two pseudo-bulk libraries: one for unstimulated PBMCs and the other for IFN-β–stimulated PBMCs. The “normalization.method” was set to “RC,” and the “scale.factor” was configured to 1,000,000 to ensure that pseudo-bulking and normalization accounted for differences in cell numbers and captured signals from lowly expressed genes. The mean for each gene was calculated, and the expression value of each gene was subtracted from its mean to visualize the results. ComplexHeatmap (Gu et al, 2016) was then employed to illustrate the differences in gene expression for the genes of interest. Genes with raw *P* value <0.05 were included in the “FindMarkers” analysis.

### Statistical analysis

GraphPad Prism version 8 or 10.0 was used for in vitro data analysis. Data were shown as mean ± SEM. The statistical significance was calculated using Student’s *t*-test with Welch’s correction or one-way or two-way ANOVA with multiple comparison tests as needed. *P* < 0.05 was considered statistically significant. Information regarding the number of experimental replicates is provided in the corresponding figure legends.

## Supplementary information

Peer Review File

Source data Fig. 1

Source data Fig. 2

Source data Fig. 3

Source data Fig. 4

Source data Fig. 5

Figure EV1 Source Data

Figure EV2 Source Data

Figure EV3 Source Data

Figure EV4 Source Data

Figure EV5 Source Data

Expanded View Figures

## Data Availability

No primary datasets have been generated and deposited. The source data of this paper are collected in the following database record: biostudies:S-SCDT-10_1038-S44319-026-00888-0.

## References

[CR1] Adachi O, Kawai T, Takeda K, Matsumoto M, Tsutsui H, Sakagami M, Nakanishi K, Akira S (1998) Targeted disruption of the MyD88 gene results in loss of IL-1- and IL-18-mediated function. Immunity 9:143–150 10.1016/s1074-7613(00)80596-8 9697844

[CR2] Au WC, Moore PA, Lowther W, Juang YT, Pitha PM (1995) Identification of a member of the interferon regulatory factor family that binds to the interferon-stimulated response element and activates expression of interferon-induced genes. Proc Natl Acad Sci USA 92:11657–11661 10.1073/pnas.92.25.11657 PMC40461 8524823

[CR3] Barnes BJ, Richards J, Mancl M, Hanash S, Beretta L, Pitha PM (2004) Global and distinct targets of IRF-5 and IRF-7 during innate response to viral infection. J Biol Chem 279:45194–45207 10.1074/jbc.M400726200 15308637

[CR4] Beutler B (2004) Innate immunity: an overview. Mol Immunol 40:845–859 10.1016/j.molimm.2003.10.005 14698223

[CR5] Chavarría-Smith J, Mitchell PS, Ho AM, Daugherty MD, Vance RE (2016) Functional and evolutionary analyses identify proteolysis as a general mechanism for NLRP1 inflammasome activation. PLos Pathog 12:e1006052 10.1371/journal.ppat.1006052 PMC5142783 27926929

[CR6] Chavarría-Smith J, Vance RE (2013) Direct proteolytic cleavage of NLRP1B is necessary and sufficient for inflammasome activation by anthrax lethal factor. PLoS Pathog 9:e1003452 10.1371/journal.ppat.1003452 PMC3688554 23818853

[CR7] Christgen S, Zheng M, Kesavardhana S, Karki R, Malireddi RKS, Banoth B, Place DE, Briard B, Sharma BR, Tuladhar S et al (2020) Identification of the PANoptosome: a molecular platform triggering pyroptosis, apoptosis, and necroptosis (PANoptosis). Front Cell Infect Microbiol 10:237 10.3389/fcimb.2020.00237 PMC7274033 32547960

[CR8] Chui AJ, Okondo MC, Rao SD, Gai K, Griswold AR, Johnson DC, Ball DP, Taabazuing CY, Orth EL, Vittimberga BA et al (2019) N-terminal degradation activates the NLRP1B inflammasome. Science 364:82–85 10.1126/science.aau1208 PMC6610862 30872531

[CR9] Dieude P, Guedj M, Wipff J, Ruiz B, Riemekasten G, Airo P, Melchers I, Hachulla E, Cerinic MM, Diot E et al (2011) NLRP1 influences the systemic sclerosis phenotype: a new clue for the contribution of innate immunity in systemic sclerosis-related fibrosing alveolitis pathogenesis. Ann Rheum Dis 70:668–674 10.1136/ard.2010.131243 21149496

[CR10] Dobin A, Davis CA, Schlesinger F, Drenkow J, Zaleski C, Jha S, Batut P, Chaisson M, Gingeras TR (2013) STAR: ultrafast universal RNA-seq aligner. Bioinformatics 29:15–21 10.1093/bioinformatics/bts635 PMC3530905 23104886

[CR11] Docherty CA, Fernando AJ, Rosli S, Lam M, Dolle RE, Navia MA, Farquhar R, La France D, Tate MD, Murphy CK et al (2023) A novel dual NLRP1 and NLRP3 inflammasome inhibitor for the treatment of inflammatory diseases. Clin Transl Immunol 12:e1455 10.1002/cti2.1455 PMC10288073 37360982

[CR12] Fenini G, Karakaya T, Hennig P, Di Filippo M, Slaufova M, Beer HD (2022) NLRP1 inflammasome activation in keratinocytes: increasing evidence of important roles in inflammatory skin diseases and immunity. J Invest Dermatol 142:2313–2322 10.1016/j.jid.2022.04.004 35550825

[CR13] Finger JN, Lich JD, Dare LC, Cook MN, Brown KK, Duraiswami C, Bertin J, Gough PJ (2012) Autolytic proteolysis within the function to find domain (FIIND) is required for NLRP1 inflammasome activity. J Biol Chem 287:25030–25037 10.1074/jbc.M112.378323 PMC3408201 22665479

[CR14] Frew BC, Joag VR, Mogridge J (2012) Proteolytic processing of Nlrp1b is required for inflammasome activity. PLoS Pathog 8:e1002659 10.1371/journal.ppat.1002659 PMC3334886 22536155

[CR15] Gessani S, Belardelli F, Pecorelli A, Puddu P, Baglioni C (1989) Bacterial lipopolysaccharide and gamma interferon induce transcription of beta interferon mRNA and interferon secretion in murine macrophages. J Virol 63:2785–2789 10.1128/jvi.63.6.2785-2789.1989 PMC250780 2498530

[CR16] Grandemange E, Sanchez E, Louis-Plence P, Rittore C, Reed J, Touitou I, Genevieve D (2015) NLRP1 mutations cause autoinflammatory diseases in human. Pediatr Rheumatol Online J 13:O22

[CR17] Grandemange S, Sanchez E, Louis-Plence P, Tran Mau-Them F, Bessis D, Coubes C, Frouin E, Seyger M, Girard M, Puechberty J et al (2017) A new autoinflammatory and autoimmune syndrome associated with NLRP1 mutations: NAIAD (NLRP1-associated autoinflammation with arthritis and dyskeratosis). Ann Rheum Dis 76:1191–1198 10.1136/annrheumdis-2016-210021 27965258

[CR18] Gu Z, Eils R, Schlesner M (2016) Complex heatmaps reveal patterns and correlations in multidimensional genomic data. Bioinformatics 32:2847–2849 10.1093/bioinformatics/btw313 27207943

[CR19] Hagar JA, Powell DA, Aachoui Y, Ernst RK, Miao EA (2013) Cytoplasmic LPS activates caspase-11: implications in TLR4-independent endotoxic shock. Science 341:1250–1253 10.1126/science.1240988 PMC3931427 24031018

[CR20] Han KJ, Su X, Xu LG, Bin LH, Zhang J, Shu HB (2004) Mechanisms of the TRIF-induced interferon-stimulated response element and NF-kappaB activation and apoptosis pathways. J Biol Chem 279:15652–15661 10.1074/jbc.M311629200 14739303

[CR21] Hao Y, Stuart T, Kowalski MH, Choudhary S, Hoffman P, Hartman A, Srivastava A, Molla G, Madad S, Fernandez-Granda C et al (2024) Dictionary learning for integrative, multimodal and scalable single-cell analysis. Nat Biotechnol 42:293–304 10.1038/s41587-023-01767-y PMC10928517 37231261

[CR22] Hollingsworth LR, Sharif H, Griswold AR, Fontana P, Mintseris J, Dagbay KB, Paulo JA, Gygi SP, Bachovchin DA, Wu H (2021) DPP9 sequesters the C terminus of NLRP1 to repress inflammasome activation. Nature 592:778–783 10.1038/s41586-021-03350-4 PMC8299537 33731932

[CR23] Honda K, Taniguchi T (2006) IRFs: master regulators of signalling by Toll-like receptors and cytosolic pattern-recognition receptors. Nat Rev Immunol 6:644–658 10.1038/nri1900 16932750

[CR24] Honda K, Yanai H, Negishi H, Asagiri M, Sato M, Mizutani T, Shimada N, Ohba Y, Takaoka A, Yoshida N et al (2005) IRF-7 is the master regulator of type-I interferon-dependent immune responses. Nature 434:772–777 10.1038/nature03464 15800576

[CR25] Hoshino K, Takeuchi O, Kawai T, Sanjo H, Ogawa T, Takeda Y, Takeda K, Akira S (1999) Cutting edge: Toll-like receptor 4 (TLR4)-deficient mice are hyporesponsive to lipopolysaccharide: evidence for TLR4 as the Lps gene product. J Immunol 162:3749–3752 10201887

[CR26] Hou G, Chen Y, Lei H, Lu Y, Liu L, Han Z, Sun S, Li J, Cheng L (2024) Bimetallic peroxide nanoparticles induce PANoptosis by disrupting ion homeostasis for enhanced immunotherapy. Sci Adv 10:eadp7160 10.1126/sciadv.adp7160 PMC11546811 39514658

[CR27] Ivashkiv LB, Donlin LT (2014) Regulation of type I interferon responses. Nat Rev Immunol 14:36–49 10.1038/nri3581 PMC4084561 24362405

[CR28] Janeway CA Jr (1989) Approaching the asymptote? Evolution and revolution in immunology. Cold Spring Harb Symp Quant Biol 54:1–13 10.1101/sqb.1989.054.01.003 2700931

[CR29] Jiang J, Zhao M, Chang C, Wu H, Lu Q (2020) Type I Interferons in the pathogenesis and treatment of autoimmune diseases. Clin Rev Allergy Immunol 59:248–272 10.1007/s12016-020-08798-2 32557263

[CR30] Jin Y, Riccardi SL, Gowan K, Fain PR, Spritz RA (2010) Fine-mapping of vitiligo susceptibility loci on chromosomes 7 and 9 and interactions with NLRP1 (NALP1). J Invest Dermatol 130:774–783 10.1038/jid.2009.273 PMC3513759 19727120

[CR31] Kang HM, Subramaniam M, Targ S, Nguyen M, Maliskova L, McCarthy E, Wan E, Wong S, Byrnes L, Lanata CM et al (2018) Multiplexed droplet single-cell RNA-sequencing using natural genetic variation. Nat Biotechnol 36:89–94 10.1038/nbt.4042 PMC5784859 29227470

[CR32] Kanneganti TD (2020) Intracellular innate immune receptors: Life inside the cell. Immunol Rev 297:5–12 10.1111/imr.12912 PMC7592123 32856334

[CR33] Kanneganti TD, Ozoren N, Body-Malapel M, Amer A, Park JH, Franchi L, Whitfield J, Barchet W, Colonna M, Vandenabeele P et al (2006) Bacterial RNA and small antiviral compounds activate caspase-1 through cryopyrin/Nalp3. Nature 440:233–236 10.1038/nature04517 16407888

[CR34] Karki R, Lee S, Mall R, Pandian N, Wang Y, Sharma BR, Malireddi RS, Yang D, Trifkovic S, Steele JA et al (2022) ZBP1-dependent inflammatory cell death, PANoptosis, and cytokine storm disrupt IFN therapeutic efficacy during coronavirus infection. Sci Immunol 7:eabo6294 10.1126/sciimmunol.abo6294 PMC9161373 35587515

[CR35] Karki R, Sharma BR, Lee E, Banoth B, Malireddi RKS, Samir P, Tuladhar S, Mummareddy H, Burton AR, Vogel P et al (2020) Interferon regulatory factor 1 regulates PANoptosis to prevent colorectal cancer. JCI insight 5:e136720 10.1172/jci.insight.136720 PMC7406299 32554929

[CR36] Karki R, Sharma BR, Tuladhar S, Williams EP, Zalduondo L, Samir P, Zheng M, Sundaram B, Banoth B, Malireddi RKS et al (2021a) Synergism of TNF-alpha and IFN-gamma triggers inflammatory cell death, tissue damage, and mortality in SARS-CoV-2 infection and cytokine shock syndromes. Cell 184:149–168.e17 10.1016/j.cell.2020.11.025 PMC7674074 33278357

[CR37] Karki R, Sundaram B, Sharma BR, Lee S, Malireddi RKS, Nguyen LN, Christgen S, Zheng M, Wang Y, Samir P et al (2021b) ADAR1 restricts ZBP1-mediated immune response and PANoptosis to promote tumorigenesis. Cell Rep 37:109858 10.1016/j.celrep.2021.109858 PMC8853634 34686350

[CR38] Kawai T, Ikegawa M, Ori D, Akira S (2024) Decoding Toll-like receptors: Recent insights and perspectives in innate immunity. Immunity 57:649–673 10.1016/j.immuni.2024.03.004 38599164

[CR39] Kayagaki N, Warming S, Lamkanfi M, Vande Walle L, Louie S, Dong J, Newton K, Qu Y, Liu J, Heldens S et al (2011) Non-canonical inflammasome activation targets caspase-11. Nature 479:117–121 10.1038/nature10558 22002608

[CR40] Kayagaki N, Wong MT, Stowe IB, Ramani SR, Gonzalez LC, Akashi-Takamura S, Miyake K, Zhang J, Lee WP, Muszynski A et al (2013) Noncanonical inflammasome activation by intracellular LPS independent of TLR4. Science 341:1246–1249 10.1126/science.1240248 23887873

[CR41] Kotov DI, Lee OV, Fattinger SA, Langner CA, Guillen JV, Peters JM, Moon A, Burd EM, Witt KC, Stetson DB et al (2023) Early cellular mechanisms of type I interferon-driven susceptibility to tuberculosis. Cell 186:5536–5553.e22 10.1016/j.cell.2023.11.002 PMC10757650 38029747

[CR42] Kovarova M, Hesker PR, Jania L, Nguyen M, Snouwaert JN, Xiang Z, Lommatzsch SE, Huang MT, Ting JP, Koller BH (2012) NLRP1-dependent pyroptosis leads to acute lung injury and morbidity in mice. J Immunol 189:2006–2016 10.4049/jimmunol.1201065 PMC3635067 22753929

[CR43] Kuriakose T, Man SM, Malireddi RK, Karki R, Kesavardhana S, Place DE, Neale G, Vogel P, Kanneganti TD (2016) ZBP1/DAI is an innate sensor of influenza virus triggering the NLRP3 inflammasome and programmed cell death pathways. Sci Immunol 1:aag2045 10.1126/sciimmunol.aag2045 PMC5131924 27917412

[CR44] Lee FFY, Alper S (2022) Alternative pre-mRNA splicing as a mechanism for terminating Toll-like Receptor signaling. Front Immunol 13:1023567 10.3389/fimmu.2022.1023567 PMC9755862 36531997

[CR45] Lee S, Karki R, Wang Y, Nguyen LN, Kalathur RC, Kanneganti TD (2021) AIM2 forms a complex with pyrin and ZBP1 to drive PANoptosis and host defence. Nature 597:415–419 10.1038/s41586-021-03875-8 PMC8603942 34471287

[CR46] Levinsohn JL, Newman ZL, Hellmich KA, Fattah R, Getz MA, Liu S, Sastalla I, Leppla SH, Moayeri M (2012) Anthrax lethal factor cleavage of Nlrp1 is required for activation of the inflammasome. PLoS Pathog 8:e1002638 10.1371/journal.ppat.1002638 PMC3315489 22479187

[CR47] Love MI, Huber W, Anders S (2014) Moderated estimation of fold change and dispersion for RNA-seq data with DESeq2. Genome Biol 15:550 10.1186/s13059-014-0550-8 PMC4302049 25516281

[CR48] Man SM, Karki R, Malireddi RK, Neale G, Vogel P, Yamamoto M, Lamkanfi M, Kanneganti TD (2015) The transcription factor IRF1 and guanylate-binding proteins target activation of the AIM2 inflammasome by Francisella infection. Nat Immunol 16:467–475 10.1038/ni.3118 PMC4406811 25774715

[CR49] Man SM, Karki R, Sasai M, Place DE, Kesavardhana S, Temirov J, Frase S, Zhu Q, Malireddi RKS, Kuriakose T et al (2016) IRGB10 liberates bacterial ligands for sensing by the AIM2 and caspase-11-NLRP3 inflammasomes. Cell 167:382–396.e17 10.1016/j.cell.2016.09.012 PMC5074697 27693356

[CR50] Martinon F, Burns K, Tschopp J (2002) The inflammasome: a molecular platform triggering activation of inflammatory caspases and processing of proIL-beta. Mol Cell 10:417–426 10.1016/s1097-2765(02)00599-3 12191486

[CR51] Martinon F, Hofmann K, Tschopp J (2001) The pyrin domain: a possible member of the death domain-fold family implicated in apoptosis and inflammation. Curr Biol 11:R118–R120 10.1016/s0960-9822(01)00056-2 11250163

[CR52] Maver A, Lavtar P, Ristic S, Stopinsek S, Simcic S, Hocevar K, Sepcic J, Drulovic J, Pekmezovic T, Novakovic I et al (2017) Identification of rare genetic variation of NLRP1 gene in familial multiple sclerosis. Sci Rep 7:3715 10.1038/s41598-017-03536-9 PMC5473861 28623311

[CR53] McNab F, Mayer-Barber K, Sher A, Wack A, O’Garra A (2015) Type I interferons in infectious disease. Nat Rev Immunol 15:87–103 10.1038/nri3787 PMC7162685 25614319

[CR54] Mudge JM, Carbonell-Sala S, Diekhans M, Martinez JG, Hunt T, Jungreis I, Loveland JE, Arnan C, Barnes I, Bennett R et al (2025) GENCODE 2025: reference gene annotation for human and mouse. Nucleic Acids Res 53:D966–D975 10.1093/nar/gkae1078 PMC11701607 39565199

[CR55] Muller U, Steinhoff U, Reis LF, Hemmi S, Pavlovic J, Zinkernagel RM, Aguet M (1994) Functional role of type I and type II interferons in antiviral defense. Science 264:1918–1921 10.1126/science.8009221 8009221

[CR56] Novick D, Cohen B, Rubinstein M (1994) The human interferon alpha/beta receptor: characterization and molecular cloning. Cell 77:391–400 10.1016/0092-8674(94)90154-6 8181059

[CR57] Okondo MC, Johnson DC, Sridharan R, Go EB, Chui AJ, Wang MS, Poplawski SE, Wu W, Liu Y, Lai JH et al (2017) DPP8 and DPP9 inhibition induces pro-caspase-1-dependent monocyte and macrophage pyroptosis. Nat Chem Biol 13:46–53 10.1038/nchembio.2229 PMC5477230 27820798

[CR58] Okondo MC, Rao SD, Taabazuing CY, Chui AJ, Poplawski SE, Johnson DC, Bachovchin DA (2018) Inhibition of Dpp8/9 Activates the Nlrp1b Inflammasome. Cell Chem Biol 25:262–267.e265 10.1016/j.chembiol.2017.12.013 PMC5856610 29396289

[CR59] Owens T, Khorooshi R, Wlodarczyk A, Asgari N (2014) Interferons in the central nervous system: a few instruments play many tunes. Glia 62:339–355 10.1002/glia.22608 24588027

[CR60] Platanias LC (2005) Mechanisms of type-I- and type-II-interferon-mediated signalling. Nat Rev Immunol 5:375–386 10.1038/nri1604 15864272

[CR61] Pontillo A, Catamo E, Arosio B, Mari D, Crovella S (2012) NALP1/NLRP1 genetic variants are associated with Alzheimer disease. Alzheimer Dis Assoc Disord 26:277–281 10.1097/WAD.0b013e318231a8ac 21946017

[CR62] Sandstrom A, Mitchell PS, Goers L, Mu EW, Lesser CF, Vance RE (2019) Functional degradation: a mechanism of NLRP1 inflammasome activation by diverse pathogen enzymes. Science 364:eaau1330 10.1126/science.aau1330 PMC6532986 30872533

[CR63] Sato M, Suemori H, Hata N, Asagiri M, Ogasawara K, Nakao K, Nakaya T, Katsuki M, Noguchi S, Tanaka N et al (2000) Distinct and essential roles of transcription factors IRF-3 and IRF-7 in response to viruses for IFN-alpha/beta gene induction. Immunity 13:539–548 10.1016/s1074-7613(00)00053-4 11070172

[CR64] Schafer SL, Lin R, Moore PA, Hiscott J, Pitha PM (1998) Regulation of type I interferon gene expression by interferon regulatory factor-3. J Biol Chem 273:2714–2720 10.1074/jbc.273.5.2714 9446577

[CR65] Sharma BR, Choudhury SM, Abdelaal HM, Wang Y, Kanneganti TD (2025) Innate immune sensor NLRP3 drives PANoptosome formation and PANoptosis. J Immunol 214:1236–1246 10.1093/jimmun/vkaf042 PMC12207079 40249072

[CR66] Sharma BR, Kanneganti TD (2021) NLRP3 inflammasome in cancer and metabolic diseases. Nat Immunol 22:550–559 10.1038/s41590-021-00886-5 PMC8132572 33707781

[CR67] Sharma BR, Karki R, Rajesh Y, Kanneganti TD (2023) Immune regulator IRF1 contributes to ZBP1-, AIM2-, RIPK1-, and NLRP12-PANoptosome activation and inflammatory cell death (PANoptosis). J Biol Chem 299:105141 10.1016/j.jbc.2023.105141 PMC10494469 37557956

[CR68] Sui J, Li H, Fang Y, Liu Y, Li M, Zhong B, Yang F, Zou Q, Wu Y (2012) NLRP1 gene polymorphism influences gene transcription and is a risk factor for rheumatoid arthritis in Han Chinese. Arthritis Rheum 64:647–654 10.1002/art.33370 21976003

[CR69] Sundaram B, Pandian N, Kim HJ, Abdelaal HM, Mall R, Indari O, Sarkar R, Tweedell RE, Alonzo EQ, Klein J et al (2024) NLRC5 senses NAD(+) depletion, forming a PANoptosome and driving PANoptosis and inflammation. Cell 187:4061–4077.e17 10.1016/j.cell.2024.05.034 PMC11283362 38878777

[CR70] Sundaram B, Pandian N, Mall R, Wang Y, Sarkar R, Kim HJ, Malireddi RKS, Karki R, Janke LJ, Vogel P et al (2023) NLRP12-PANoptosome activates PANoptosis and pathology in response to heme and PAMPs. Cell 186:2783–2801.e20 10.1016/j.cell.2023.05.005 PMC10330523 37267949

[CR71] Takeuchi O, Akira S (2010) Pattern recognition receptors and inflammation. Cell 140:805–820 10.1016/j.cell.2010.01.022 20303872

[CR72] Tweedell RE, Hibler T, Kanneganti TD (2024) Defining PANoptosis: biochemical and mechanistic evaluation of innate immune cell death activation. Curr Protoc 4:e1112 10.1002/cpz1.1112 PMC11581195 39073015

[CR73] Ueda MT, Inamo J, Miya F, Shimada M, Yamaguchi K, Kochi Y (2024) Functional and dynamic profiling of transcript isoforms reveals essential roles of alternative splicing in interferon response. Cell Genom 4:100654 10.1016/j.xgen.2024.100654 PMC11602592 39288763

[CR74] Verma D, Bivik C, Farahani E, Synnerstad I, Fredrikson M, Enerback C, Rosdahl I, Soderkvist P (2012) Inflammasome polymorphisms confer susceptibility to sporadic malignant melanoma. Pigment Cell Melanoma Res 25:506–513 10.1111/j.1755-148X.2012.01008.x 22524199

[CR75] Wang L, Zhu Y, Zhang N, Xian Y, Tang Y, Ye J, Reza F, He G, Wen X, Jiang X (2024) The multiple roles of interferon regulatory factor family in health and disease. Signal Transduct Target Ther 9:282 10.1038/s41392-024-01980-4 PMC11486635 39384770

[CR76] Xu H, Shi J, Gao H, Liu Y, Yang Z, Shao F, Dong N (2019) The N-end rule ubiquitin ligase UBR2 mediates NLRP1B inflammasome activation by anthrax lethal toxin. EMBO J 38:e101996 10.15252/embj.2019101996 PMC6600268 31268597

[CR77] Yamamoto M, Sato S, Hemmi H, Hoshino K, Kaisho T, Sanjo H, Takeuchi O, Sugiyama M, Okabe M, Takeda K et al (2003) Role of adaptor TRIF in the MyD88-independent toll-like receptor signaling pathway. Science 301:640–643 10.1126/science.1087262 12855817

[CR78] Yap JKY, Pickard BS, Chan EWL, Gan SY (2019) The role of neuronal NLRP1 inflammasome in Alzheimer’s disease: bringing neurons into the neuroinflammation game. Mol Neurobiol 56:7741–7753 10.1007/s12035-019-1638-7 31111399

[CR79] Zhang Z, Shibata T, Fujimura A, Kitaura J, Miyake K, Ohto U, Shimizu T (2023) Structural basis for thioredoxin-mediated suppression of NLRP1 inflammasome. Nature 622:188–194 10.1038/s41586-023-06532-4 37704723

[CR80] Zhong FL, Mamai O, Sborgi L, Boussofara L, Hopkins R, Robinson K, Szeverenyi I, Takeichi T, Balaji R, Lau A et al (2016) Germline NLRP1 mutations cause skin inflammatory and cancer susceptibility syndromes via inflammasome activation. Cell 167:187–202.e17 10.1016/j.cell.2016.09.001 27662089

[CR81] Zhong FL, Robinson K, Teo DET, Tan KY, Lim C, Harapas CR, Yu CH, Xie WH, Sobota RM, Au VB et al (2018) Human DPP9 represses NLRP1 inflammasome and protects against autoinflammatory diseases via both peptidase activity and FIIND domain binding. J Biol Chem 293:18864–18878 10.1074/jbc.RA118.004350 PMC6295727 30291141

[CR82] Zurawek M, Fichna M, Januszkiewicz-Lewandowska D, Gryczynska M, Fichna P, Nowak J (2010) A coding variant in NLRP1 is associated with autoimmune Addison’s disease. Hum Immunol 71:530–534 10.1016/j.humimm.2010.02.004 20152874

