# Supplementary material for: IFN-α and IFN-β inhibit NLRP1-driven PANoptosis
Source: EMBO Rep. 2026 Jul 29;27(18):5214–32. doi: 10.1038/s44319-026-00888-0 (PMC13601632; doi:10.1038/s44319-026-00888-0)
Supplement: Supplementary file 1 [file 44319_2026_888_MOESM12_ESM.pdf]

Expanded View Figures

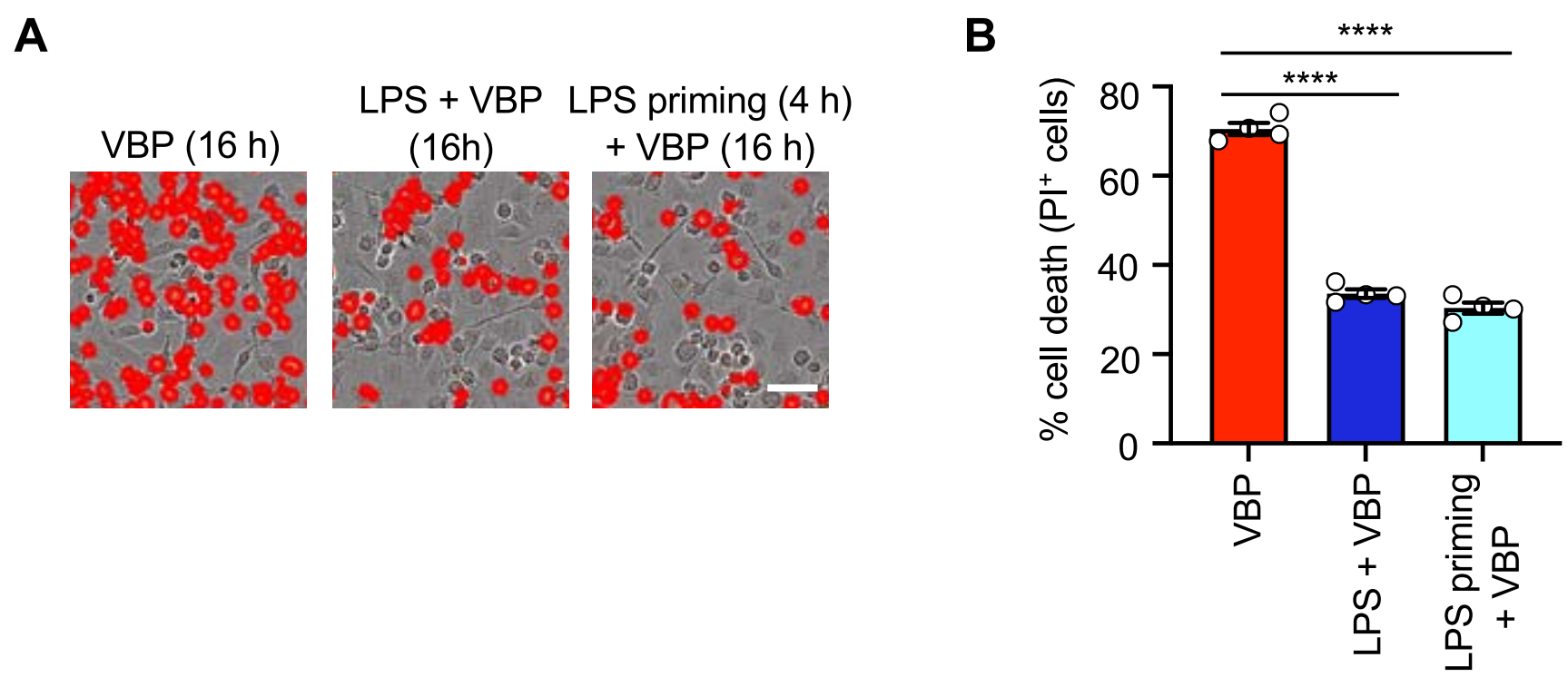

Figure EV1. LPS priming inhibits VBP-induced cell death.

(**A**, **B**) Representative images (**A**) and quantification (**B**) of cell death in wild-type mouse bone marrow-derived macrophages (BMDMs) at 16 h after treatment with Val-boroPro (VBP), simultaneous lipopolysaccharide (LPS) plus VBP, or LPS (4 h priming) followed by VBP. (**A**, **B**) Data were representative of three independent biological replicates. (**B**) $n = 4$ four technical replicates. Scale bar: 50 µm (**A**). ****$P < 0.0001$. One-way ANOVA with Dunnett's multiple comparison test was used. The data were represented as mean ± SEM in (**B**). Source data are available online for this figure.

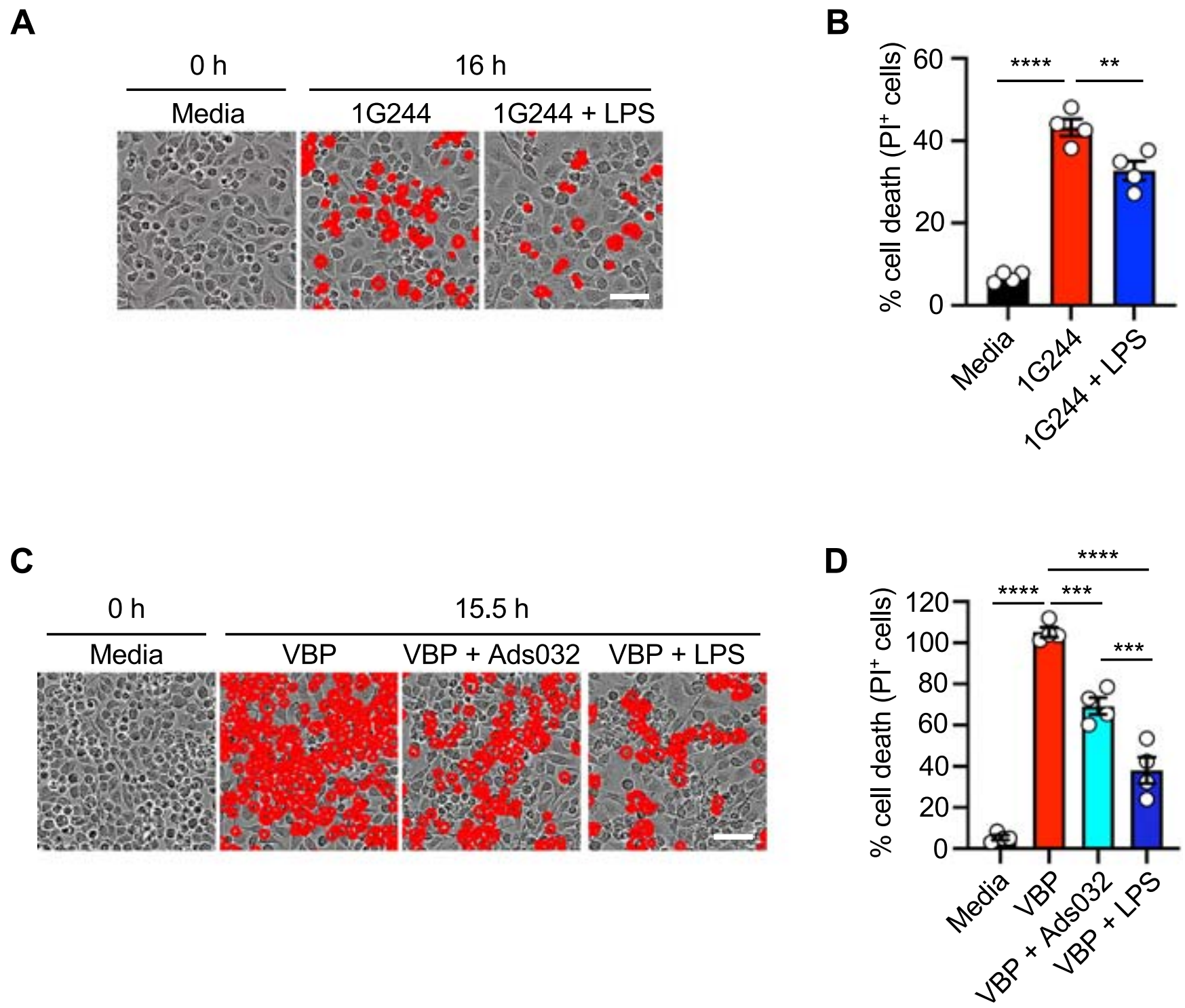

Figure EV2. LPS protects against NLRP1-mediated cell death and is more potent than the NLRP1 inhibitor Ads032.

Representative images (**A**, **C**) and quantification (**B**, **D**) of cell death in wild-type (WT) mouse bone marrow-derived macrophages (BMDMs) in media (0 h) or after treatment with (**A**, **B**) 1G244 or 1G244 plus lipopolysaccharide (LPS) for 16 h, or (**C**, **D**) Val-boroPro (VBP), VBP plus Ads032, or VBP plus LPS for 15.5 h. (**A–D**) Data were representative of three independent biological replicates. (**B**, **D**) $n = 4$ technical replicates. Scale bar: 50 µm (**A**, **C**). (**B**) Media vs 1G244, **** $P < 0.0001$; 1G244 vs 1G244 + LPS, ** $P = 0.0049$. (**D**) Media vs VBP, **** $P < 0.0001$; VBP vs VBP + Ads032, *** $P = 0.0002$; VBP vs VBP + LPS, **** $P < 0.0001$; VBP + Ads032 vs VBP + LPS, *** $P = 0.0006$. One-way ANOVA with (**B**) Dunnett's and (**D**) Tukey's multiple comparison test were used. The data were represented as mean ± SEM in (**B**, **D**). Source data are available online for this figure.

 © The Author(s)

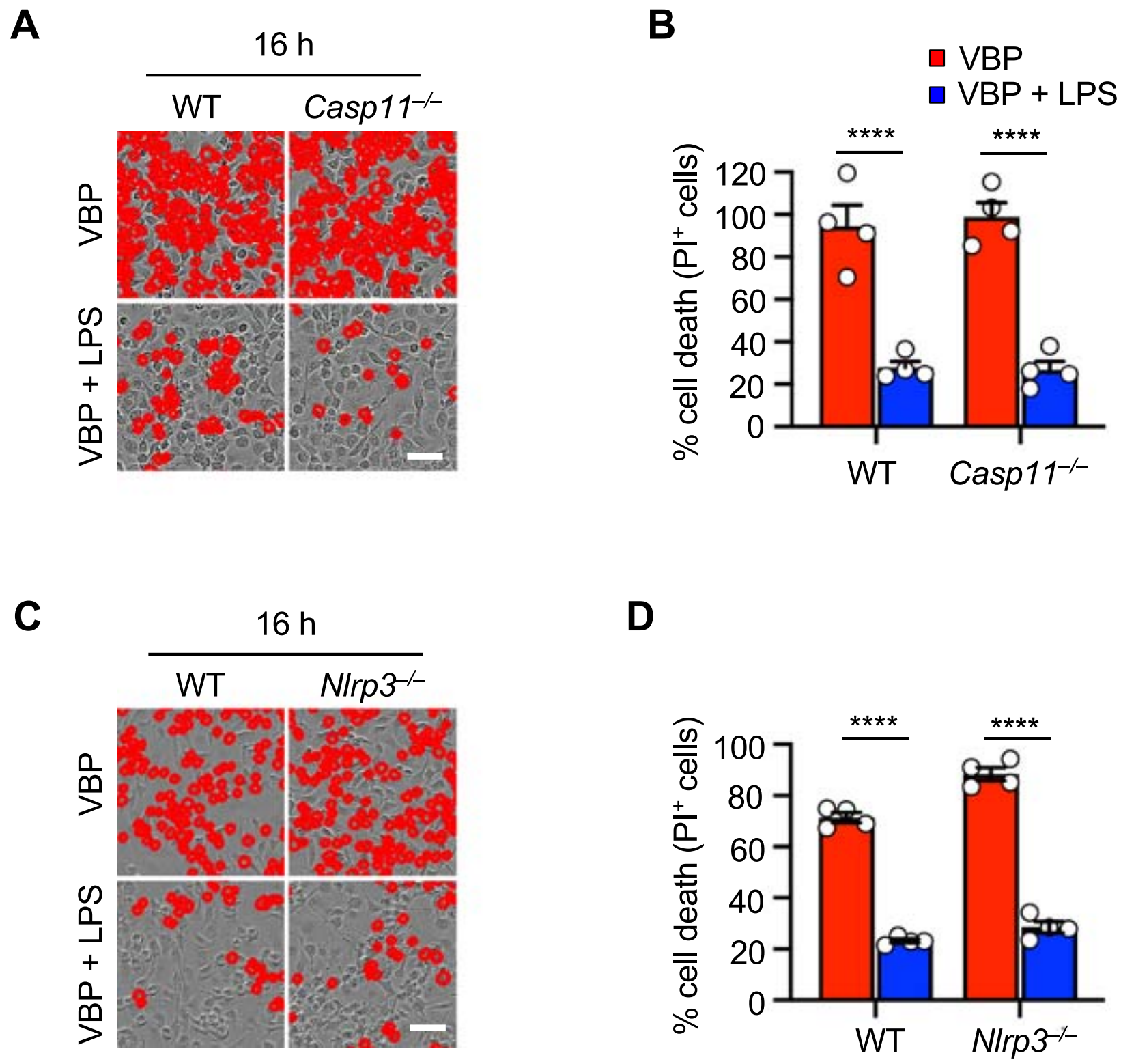

Figure EV3. Caspase-11 and NLRP3 are not required for LPS to block VBP-induced cell death.

(**A**, **B**) Representative images (**A**) and quantification (**B**) of cell death in wild type (WT) and *Casp11*$^{-/-}$ mouse bone marrow-derived macrophages (BMDMs) at 16 h after treatment with Val-boroPro (VBP) or VBP plus lipopolysaccharide (LPS). (**C**, **D**) Representative images (**C**) and quantification (**D**) of cell death in WT and *Nlrp3*$^{-/-}$ BMDMs at 16 h after treatment with VBP or VBP plus LPS. (**A**–**D**) Data were representative of three independent biological replicates. (**B**, **D**) $n = 4$ technical replicates. Scale bar: 50 µm (**A**, **C**). ****$P < 0.0001$ (two-way ANOVA with Sidak's multiple comparison test). The data were represented as mean ± SEM in (**B**, **D**). Source data are available online for this figure.

© The Author(s)

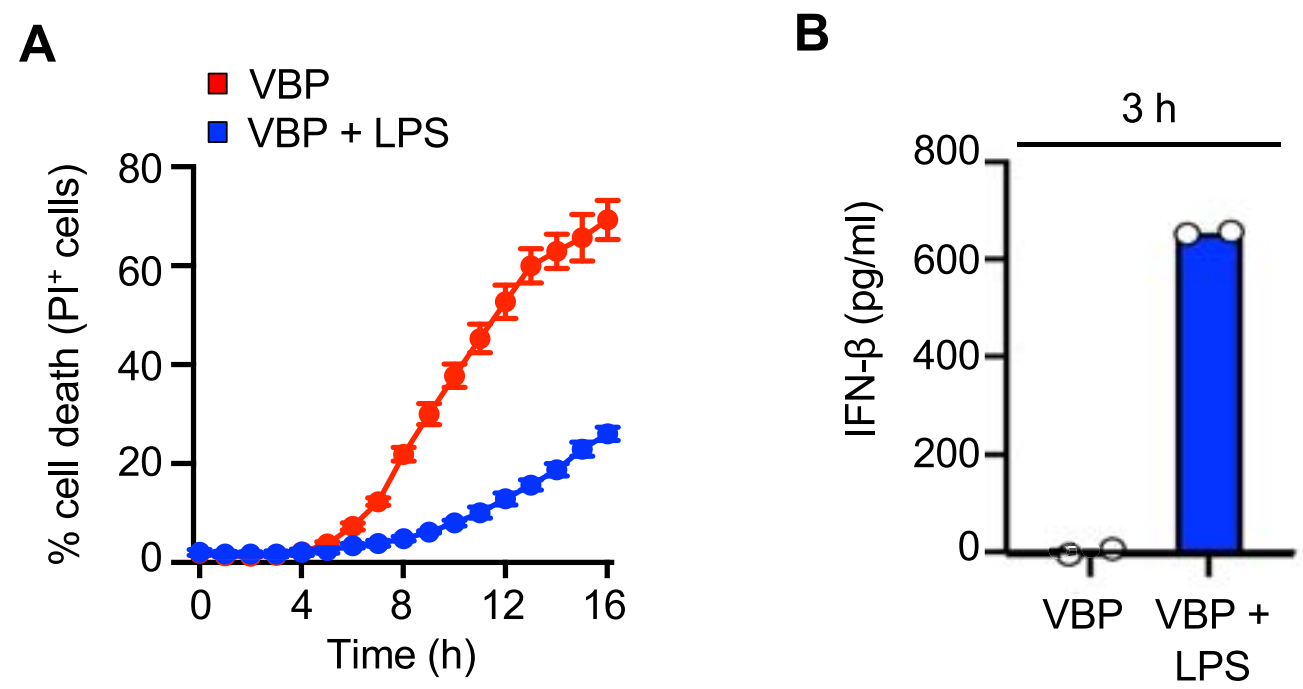

Figure EV4. LPS induces IFN-β production prior to the onset of cell death.

(**A**) Cell death kinetics in wild-type (WT) mouse bone marrow-derived macrophages (BMDMs) following treatment with Val-boroPro (VBP) or VBP plus lipopolysaccharide (LPS). (**B**) Quantification of IFN-β in the supernatant of WT BMDMs at 3 h after treatment with VBP or VBP + LPS. (**A**, **B**) Data were representative of three independent biological replicates. $n = 4$ (**A**) or 2 (**B**) technical replicates. The data were represented as mean ± SEM in (**A**) and mean in (**B**). Source data are available online for this figure.

 © The Author(s)

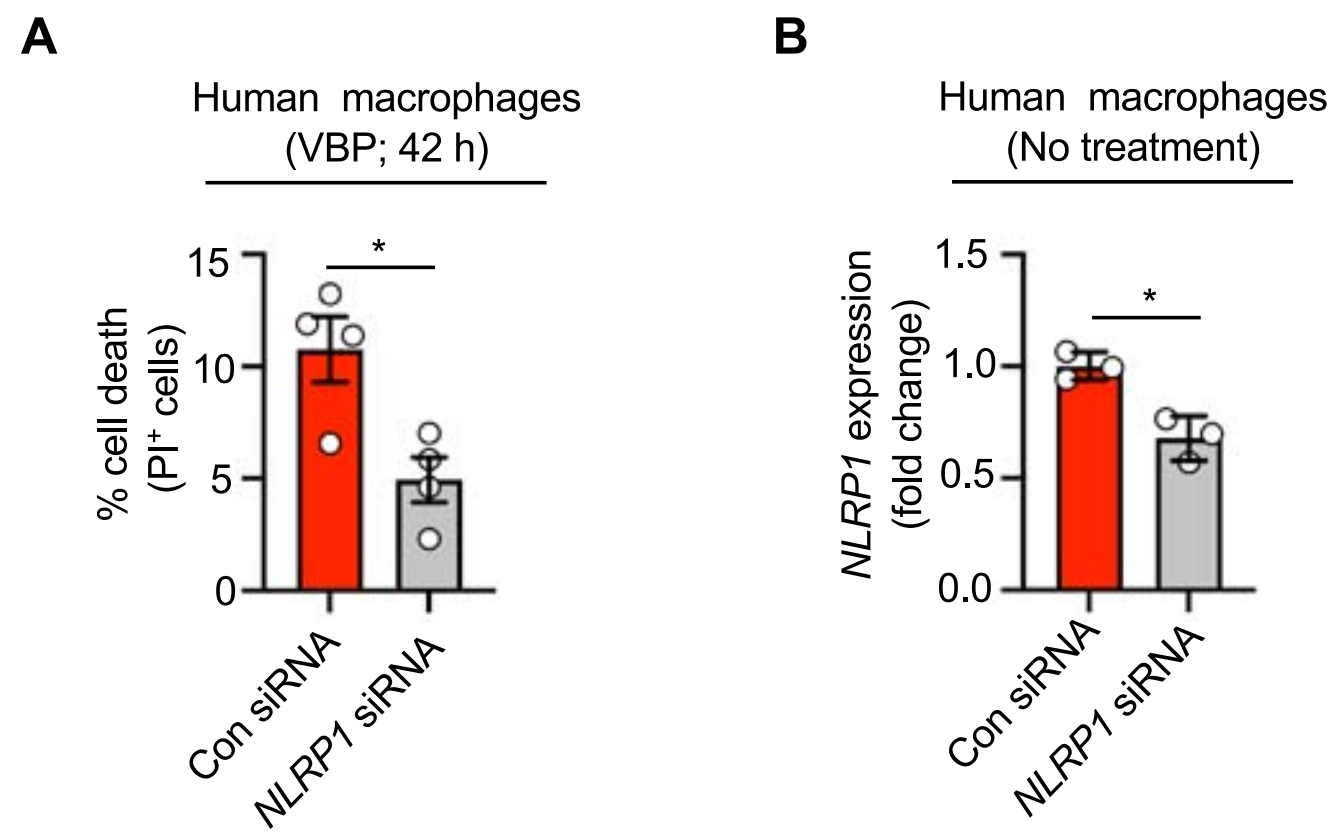

Figure EV5. NLRP1 drives VBP-induced cell death in human macrophages.

(**A**) Quantification of cell death in the indicated human macrophages at 42 h after treatment with Val-boroPro (VBP). (**B**) *NLRP1* gene expression after silencing with control siRNA (Con siRNA) or *NLRP1* siRNA. qRT-PCR data were expressed as fold change relative to the siRNA control, using *18S* as the housekeeping gene. (**A**, **B**) Data were representative of three independent biological replicates. $n = 4$ (left) or 3 (right) technical replicates. (**A**) Con siRNA vs *NLRP1* siRNA, $^{*}P = 0.0197$. (**B**) Con siRNA vs *NLRP1* siRNA, $^{*}P = 0.0136$ (Welch's *t*-test). The data were represented as mean ± SEM. Source data are available online for this figure.
