# Supplementary material for: IFN-α and IFN-β inhibit NLRP1-driven PANoptosis
Source: EMBO Rep. 2026 Jul 29;27(18):5214–32. doi: 10.1038/s44319-026-00888-0 (PMC13601632; doi:10.1038/s44319-026-00888-0)
Supplement: Supplementary file 2 [file 44319_2026_888_MOESM1_ESM.pdf]

EMBO reports

IFN-α and IFN-β inhibit NLRP1-driven PANoptosis

Bhesh Sharma, Harisankeerth Mummareddy, Sangappa Chadchan, Roman Sarkar, Chadi El Farran, and Thirumala-Devi Kanneganti

Corresponding author(s): Thirumala-Devi Kanneganti (Thirumala-Devi.Kanneganti@stjude.org)

Review Timeline:

Transfer Date:	4th May 25
Editorial Decision:	4th Jun 25
Revision Received:	24th Apr 26
Editorial Decision:	16th Jun 26
Revision Received:	10th Jul 26
Accepted:	14th Jul 26

Editor: Achim Breiling

Transaction Report: This manuscript was transferred to EMBO Reports following peer review at The EMBO Journal.

(Note: With the exception of the correction of typographical or spelling errors that could be a source of ambiguity, letters and reports are not edited. Depending on transfer agreements, referee reports obtained elsewhere may or may not be included in this compilation. Referee reports are anonymous unless the Referee chooses to sign their reports.)

1st Editorial Decision 4th Jun 2025

Dear Dr. Kanneganti,

Thank you for transferring your manuscript to EMBO reports. I now went again through the manuscript, the referee reports from The EMBO Journal and the input from the advisor (attached again below) and your revision plan (provisional point-by-point response).

Judging from your revision plan, it seems that you will be able to adequately address the concerns of the advisor and the referees during a major revision. I thus invite you to revise your manuscript accordingly with the understanding that all referee concerns must be addressed in the revised manuscript and/or in a final detailed point-by-point response, as indicated in your revision plan.

Revised manuscripts should be submitted within three months of a request for revision. Please contact me to discuss the revision (also by video chat) if you have further questions or comments regarding the revision, or should you need additional time.

Acceptance of your manuscript will depend on a positive outcome of another round of review at EMBO reports, using the same referees.

When submitting your revised manuscript, please also carefully review the instructions that follow below.

PLEASE NOTE THAT upon resubmission revised manuscripts are subjected to an initial quality control prior to exposition to re-review. Upon failure in the initial quality control, the manuscripts are sent back to the authors, which may lead to delays. Frequent reasons for such a failure are the lack of the data availability section (please see below) and the presence of statistics based on n=2 (the authors are then asked to present scatter plots or provide more data points).

When submitting your revised manuscript, we will require:

1) a .docx formatted version of the final manuscript text (including legends for main figures, EV figures and tables), but without the figures included. Please make sure that changes are highlighted to be clearly visible. Figure legends should be compiled at the end of the manuscript text.

2) individual production quality figure files as .eps, .tif, .jpg (one file per figure), of main figures and EV figures. Please upload these as separate, individual files upon re-submission. Please make sure that all figure panels are called out separately and sequentially in the manuscript text

The Expanded View format, which will be displayed in the main HTML of the paper in a collapsible format, has replaced the Supplementary information. You can submit up to 5 images as Expanded View. Please follow the nomenclature Figure EV1, Figure EV2 etc. The figure legend for these should be included in the main manuscript document file in a section called Expanded View Figure Legends after the main Figure Legends section. Additional Supplementary material should be supplied as a single pdf file labeled Appendix. The Appendix should have page numbers and needs to include a table of content on the first page (with page numbers) and legends for all content. Please follow the nomenclature Appendix Figure Sx, Appendix Table Sx etc. throughout the text, and also label the figures and tables according to this nomenclature.

For more details please refer to our guide to authors:
http://www.embopress.org/page/journal/14693178/authorguide#manuscriptpreparation

See also our guide for figure preparation:
http://wol-prod-cdn.literatumonline.com/pb-assets/embo-site/EMBOPress_Figure_Guidelines_061115-1561436025777.pdf

Moreover, please consult our guidelines for figure legend preparation:
https://www.embopress.org/page/journal/14693178/authorguide#figureformat

3) a .docx formatted letter INCLUDING the reviewers' reports and your detailed point-by-point responses to their comments. As part of the EMBO Press transparent editorial process, the point-by-point response is part of the Review Process File (RPF), which will be published alongside your paper.

4) a complete author checklist, which you can download from our author guidelines (https://www.embopress.org/page/journal/14693178/authorguide). Please insert page numbers in the checklist to indicate where the requested information can be found in the manuscript. The completed author checklist will also be part of the RPF.

Please also follow our guidelines for the use of living organisms, and the respective reporting guidelines:
http://www.embopress.org/page/journal/14693178/authorguide#livingorganisms

5) that primary datasets produced in this study (e.g. RNA-seq, ChIP-seq and array data) are deposited in an appropriate public database. This is now mandatory (like the COI statement). If no primary datasets have been deposited in any database, please state this in this section (e.g. 'No primary datasets have been generated and deposited').

See also: http://embor.embopress.org/authorguide#datadeposition

Please remember to provide a reviewer password if the datasets are not yet public.

The accession numbers and database should be listed in a formal "Data Availability " section (placed after Materials & Methods) that follows the model below. Please note that the Data Availability Section is restricted to new primary data that are part of this study.

Data availability

The datasets produced in this study are available in the following databases:

- RNA-Seq data: Gene Expression Omnibus GSE46843 (https://www.ncbi.nlm.nih.gov/geo/query/acc.cgi?acc=GSE46843)
- [data type]: [name of the resource] [accession number/identifier/doi] ([URL or identifiers.org/DATABASE:ACCESSION])

*** Note - All links should resolve to a page where the data can be accessed. ***

Moreover, I have these editorial requests:

6) We now request the publication of original source data with the aim of making primary data more accessible and transparent to the reader. You will receive a separate email with instructions for providing source data with your revised manuscript, including information how to upload and organize the files.

7) Our journal encourages inclusion of *data citations in the reference list* to directly cite datasets that were re-used and obtained from public databases. Data citations in the article text are distinct from normal bibliographical citations and should directly link to the database records from which the data can be accessed. In the main text, data citations are formatted as follows: "Data ref: Smith et al, 2001" or "Data ref: NCBI Sequence Read Archive PRJNA342805, 2017". In the Reference list, data citations must be labeled with "[DATASET]". A data reference must provide the database name, accession number/identifiers and a resolvable link to the landing page from which the data can be accessed at the end of the reference. Further instructions are available at: http://www.embopress.org/page/journal/14693178/authorguide#referencesformat

8) Regarding data quantification and statistics, please make sure that the number "n" for how many independent experiments were performed, their nature (biological versus technical replicates), the bars and error bars (e.g. SEM, SD) and the test used to calculate p-values is indicated in the respective figure legends (also for EV and Appendix figures). Please also check that all the p-values are explained in the legend, and that these fit to those shown in the figure. Please provide statistical testing where applicable. Please avoid the phrase 'independent experiment', but clearly state if these were biological or technical replicates. Please also indicate (e.g. with n.s.) if testing was performed, but the differences are not significant. In case n=2, please show the data as separate datapoints without error bars and statistics. See also: http://www.embopress.org/page/journal/14693178/authorguide#statisticalanalysis

If n<5, please show single datapoints for diagrams.

9) Please add scale bars of similar style and thickness to microscopic images, using clearly visible black or white bars (depending on the background). Please place these in the lower right corner of the images themselves. Please do not write on or near the bars in the image but define the size in the respective figure legend.

10) Please also note our reference format: http://www.embopress.org/page/journal/14693178/authorguide#referencesformat

11) We updated our journal's competing interests policy in January 2022 and request authors to consider both actual and perceived competing interests. Please review the policy https://www.embopress.org/competing-interests and update your competing interests if necessary. Please name this section 'Disclosure and Competing Interests Statement' and put it after the Acknowledgements section.

12) We now use CRediT to specify the contributions of each author in the journal submission system. CRediT replaces the author contribution section. Please use the free text box to provide more detailed descriptions and do NOT provide your final manuscript text file with an author contributions section. See also our guide to authors: https://www.embopress.org/page/journal/14693178/authorguide#authorshipguidelines

13) All Materials and Methods need to be described in the main text using our 'Structured Methods' format, which is required for all research articles. According to this format, the Methods section should include a Reagents and Tools Table (listing key reagents, experimental models, software, and relevant equipment and including their sources and relevant identifiers), uploaded as separate file, and a Methods section in which we encourage the authors to describe their methods using a step-by-step protocol format with bullet points, to facilitate the adoption of the methodologies across labs. More information on how to adhere to this format as well as downloadable templates (.doc) for the Reagents and Tools Table can be found in our author guidelines (section 'Structured Methods'):

https://www.embopress.org/page/journal/14693178/authorguide#manuscriptpreparation

14) Please restrict the number of keywords to 5 and order the sections like this, using these names:
Title page - Abstract (not more than 175 words) - Keywords - Introduction - Results - Discussion - Methods - Data availability section - Acknowledgements (please include here also the funding information) - Disclosure and Competing Interests Statement - References - Figure legends - Expanded View Figure legends

15) Please make sure that all the funding information is also entered into the online submission system and that it is complete and similar to the one in the acknowledgement section of the manuscript text file.

We would also welcome the submission of cover suggestions, or motifs to be used by our Graphics Illustrator in designing a cover.

As part of the EMBO publication's Transparent Editorial Process, EMBO reports publishes online a Review Process File (RPF) to accompany accepted manuscripts. This File will be published in conjunction with your paper and will include the referee reports, your point-by-point response and all pertinent correspondence relating to the manuscript.

You are able to opt out of this by letting the editorial office know (emboreports@embo.org). If you do opt out, the Review Process File link will point to the following statement: "No Review Process File is available with this article, as the authors have chosen not to make the review process public in this case."

I look forward to seeing a revised form of your manuscript when it is ready.

Please let me know if you have questions or comments regarding the revision.

Best,

Achim

Achim Breiling
Senior Editor
EMBO Reports

Referee #1:

The authors clearly show that type I interferon (IFN I) inhibits NLRP1 activity in mouse and human myeloid cells. They begin by observing that LPS inhibits VBP-NLRP1-stimulated cytokine release and pyroptosis, and then using KO cells logically identify the means whereby LPS does this as via TRIF-IRF3-IFN I. They then show that direct treatment of cells with IFN I inhibits NLRP1, likely due to inhibition of NLRP1 expression.

Overall this is an interesting and robust observation, however the contribution to new knowledge is very incremental. Essentially the paper shows that in myeloid cells IFN I inhibits NLRP1 activity. There is no mechanistic advance, in terms of a significant increase in our understanding of how NLRP1 is regulated, and no physiological context for IFN I regulation of NLRP1.

Referee #2:

Concisely written manuscript reporting that the TRIF-IRIF3 pathway inhibits NLRP1 inhibition in murine and human cells.

Major concerns: I appreciate the concise message of the paper, but I'd like to leave it to the editor to assess the novelty . The author proposes that type I interferon be used as a potential inhibitor for NLRP1-driven inflammation. This is a bit of a stretch,. My opinion is that the antiviral effect of type I interferon (through ISGs etc) likely far outweighs NLRP1 inhibition in a therapeutic

context. I am also not aware of any prior examples of using one immunostimulatory cytokine to treat inflammatory disorders caused by another immunostimulatory cytokine.

The authors propose NLRP1 mRNA downregulation as the mechanism explaining why IRF3/type I interferon can downregulate NLRP1. This part of the findings seems a little underdeveloped. In Fig 4E, some sort of absolute quantitation of the NLRP1 transcript and NLRP1 protein is needed.

Minor concern: I am under the impression that CARD8 is the predominant sensor that drives VBP-induced pyroptosis in human macrophages. This has an impact on the findings in FIgure 4C-D. The authors should address this.

Referee #3:

In their manuscript, B. Sharma and colleagues investigate the molecular mechanisms regulating NLRP1 inflammasome activation, demonstrating that LPS-induced inhibition of NLRP1 is mediated by type I interferon. This work sheds light on the interplay between inflammatory cytokine production and inflammasome regulation in the context of inflammatory responses.

The study presents solid mechanistic insights; however, it offers limited conceptual novelty, which constitutes the primary drawback. For instance, in Figure 4E, the authors show the transcriptional regulation of various NLRs by interferon, yet this finding receives minimal attention in the text. It would be valuable for the authors to comment on the differential regulation of specific NLRs, as this may provide a broader perspective on the biological implications of their data.

Additionally, several images related to viability assays could be consolidated. Streamlining these figures would reduce redundancy and potentially condense the presentation to fewer than four figures without compromising clarity.
Despite these concerns, the mechanistic advances presented in this manuscript are appreciated and contribute meaningfully to the field.

Minor comments:
• Line 57: The structure and function of VBP should be explained for clarity.
• Line 74: The phrase "unexpectedly" is vague and should be revised or better contextualized.
• In the discussion: The inhibitory role of type I interferon is not entirely unexpected, as antagonism between type I interferon and classical NF-κB-driven inflammatory responses has been well documented, particularly in the context of tuberculosis and COVID-19.

Advisor:

1. Mechanistic Depth and Hypothesis Testing
I concur with the reviewers assessment that the study presents a robust inhibitory phenotype but lacks sufficient mechanistic insight into how LPS and IFNs inhibit NLRP1 activation. While the authors propose transcriptional downregulation of NLRP1, this is inferred solely from publicly available datasets without direct experimental validation in their system. Commercially available antibodies for both human and murine NLRP1 make this a feasible and necessary next step. Validation at both the mRNA and protein levels would significantly strengthen the mechanistic claims.

An alternative plausible hypothesis would be: LPS or IFNs might regulate NLRP1 activation at the post-translational level or modulate downstream inflammasome components. This line of inquiry, if explored, could substantially enhance the mechanistic understanding presented in the manuscript.

2. Role of IFN Production and TLR4-TRIF-IRF3 Axis
There is a critical gap regarding the role of TLR4-TRIF-IRF3-mediated IFN production in the observed LPS-dependent inhibition. Specifically, the authors do not directly demonstrate that LPS-stimulated cells produce and release sufficient levels of IFNs within the necessary timeframe to inhibit VbP-induced NLRP1 activation. This is a crucial point, as the IFN response could be a parallel rather than causative mechanism. Testing this connection, potentially using Ifnar-KO BMDMs, would directly address this concern and strengthen the causal link between IFN signaling and NLRP1 inhibition.

3. Experimental Design - Priming Strategy
There is general confusion about the experimental approach, particularly the concurrent addition of Val-boroPro and LPS or IFNs. This design appears to conflict with the hypothesis that transcriptional downregulation mediates the observed inhibition, as pre-stimulation might be more appropriate to allow for transcriptomic changes. The authors should provide a clearer rationale for this choice or consider revising their protocol accordingly.

4. Clinical Relevance and Human Data
Given the known species differences between human and mouse NLRP1, the inclusion of more human data, particularly with clinically relevant NLRP1 variants, would significantly enhance the manuscript's impact. This could involve assessing whether

LPS or Type I IFNs modulate the activation of disease-associated human NLRP1 variants or employing more physiologically relevant triggers, such as ribotoxic stress or pathogen infection.

5. Therapeutic Potential
Finally, while the authors propose potential therapeutic applications, the evidence remains preliminary. More robust human data, as noted, along with a demonstration that this modulation could influence clinically relevant NLRP1-driven pathologies, would be critical for substantiating this claim.

Summary and Recommendation
Overall, the manuscript presents an intriguing phenotype with potential clinical implications but lacks the mechanistic depth required for a definitive contribution to the field. Addressing the points raised regarding mechanistic insight, experimental design, and clinical relevance would substantially strengthen the manuscript.

We greatly appreciate the time and expertise the advisor and reviewers have contributed to the peer review process. We are thankful for the comments from Reviewer # 1 that our study provides "interesting and robust observation[s]". We are also grateful to Reviewer #2 for noting that our study is a "[c]oncisely written manuscript reporting that the TRIF-IRIF3 pathway inhibits NLRP1 inhibition in murine and human cells." Furthermore, we appreciate the comments from Reviewer # 3 that "the mechanistic advances presented in this manuscript are appreciated and contribute meaningfully to the field".

We have carefully considered and addressed all the reviewers' and advisor's comments by providing additional data to further strengthen the conclusions of our manuscript. The specific comments from the reviewers and advisor are addressed in our point-by-point response below:

Response to the advisor's comments

1. Mechanistic Depth and Hypothesis Testing
I concur with the reviewer's assessment that the study presents a robust inhibitory phenotype but lacks sufficient mechanistic insight into how LPS and IFNs inhibit NLRP1 activation. While the authors propose transcriptional downregulation of NLRP1, this is inferred solely from publicly available datasets without direct experimental validation in their system. Commercially available antibodies for both human and murine NLRP1 make this a feasible and necessary next step. Validation at both the mRNA and protein levels would significantly strengthen the mechanistic claims. An alternative plausible hypothesis would be: LPS or IFNs might regulate NLRP1 activation at the post-translational level or modulate downstream inflammasome components. This line of inquiry, if explored, could substantially enhance the mechanistic understanding presented in the manuscript.

Here, the advisor is requesting additional mechanistic insight into how LPS and type I IFNs inhibit NLRP1 activation. To address this, we first determined whether type I IFN treatment affected NLRP1 expression. Using publicly available datasets for human and murine cells, we found that *NLRP1* mRNA levels were downregulated in response to IFN-β treatment (shown below). We have included these results as Figure 5A–B in our revised manuscript.

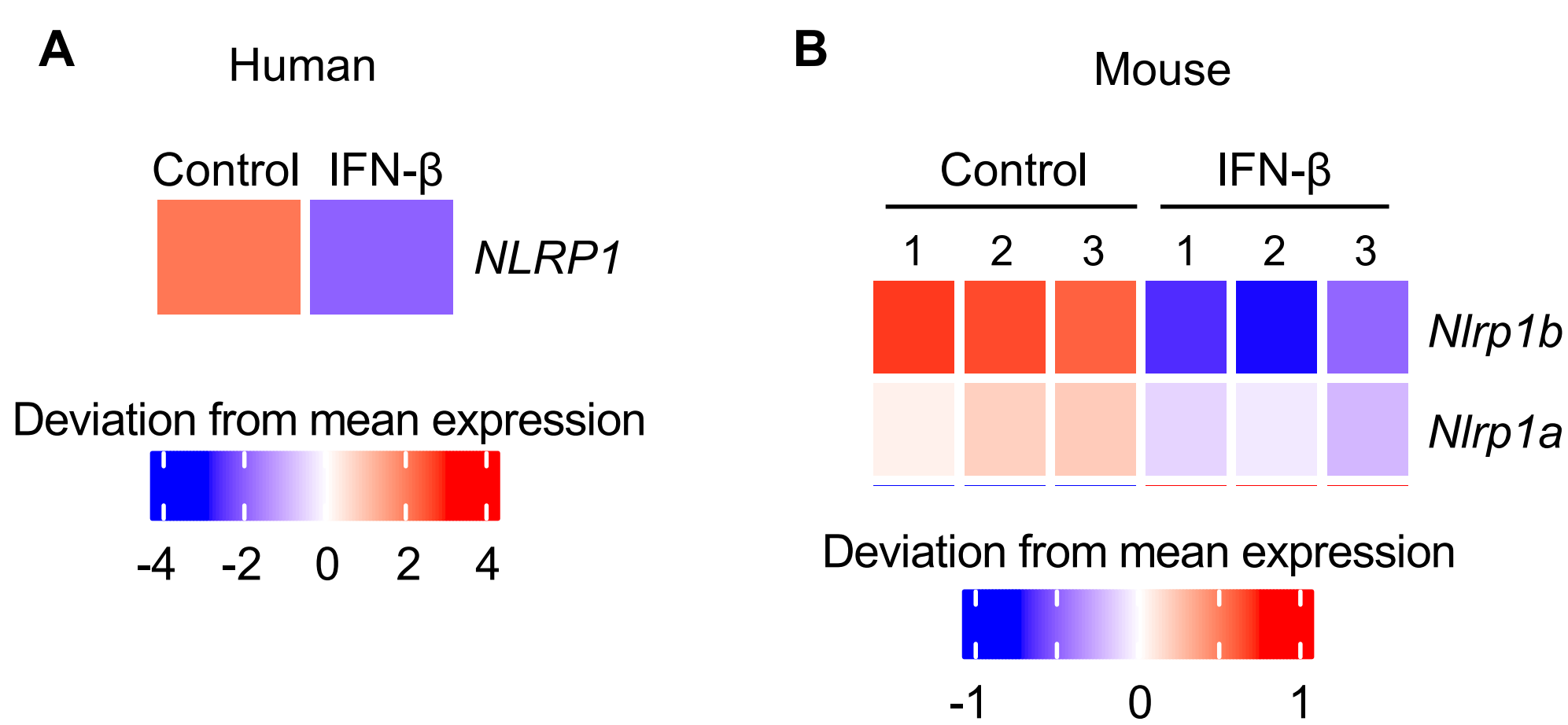

To validate these expression data, we then measured *NLRP1* mRNA levels in murine BMDMs and human macrophages and monocytes, and we found that treatment with LPS or type I IFN significantly downregulated *NLRP1* mRNA expression. Specifically, we found a significant decrease in *NLRP1* mRNA expression in both mouse and human macrophages and monocytes in response to LPS or IFN treatment when the cells were treated with VBP (shown below). These results suggest that NLRP1 is regulated at the transcriptional level. We have included these results as Figure 5C–E in our revised manuscript.

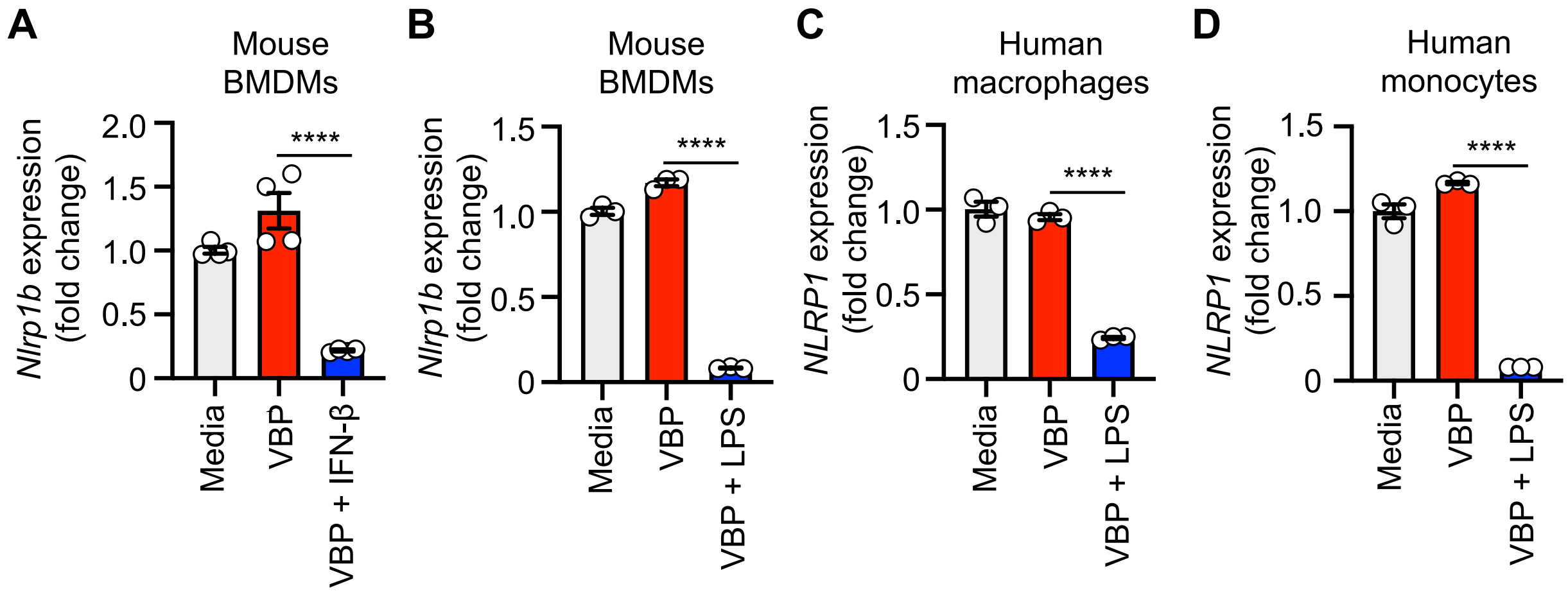

As the advisor noted, NLRP1 antibodies are listed as being commercially available, and we purchased these antibodies from multiple vendors. However, with all the antibodies we tested, we were unable to detect NLRP1 protein expression in primary macrophages. Previous studies have primarily used these antibodies to detect NLRP1 in non-immune cells such as keratinocytes or epithelial cells, rather than in primary macrophages. It is likely that the expression in macrophages falls below the limit of detection of these antibodies. Therefore, we have used the combination of RNAseq data and our independent transcriptional analysis to determine that LPS-mediated production of type I IFNs modulates NLRP1-mediated cell death by regulating the expression of NLRP1.

2. Role of IFN Production and TLR4-TRIF-IRF3 Axis
There is a critical gap regarding the role of TLR4-TRIF-IRF3-mediated IFN production in the observed LPS-dependent inhibition. Specifically, the authors do not directly demonstrate that LPS-stimulated cells produce and release sufficient levels of IFNs within the necessary timeframe to inhibit VbP-induced NLRP1 activation. This is a crucial point, as the IFN response could be a parallel rather than causative mechanism. Testing this connection, potentially using Ifnar-KO BMDMs, would directly address this concern and strengthen the causal link between IFN signaling and NLRP1 inhibition.

Here, the advisor is requesting confirmation that IFN production is the causative mechanism, rather than a parallel phenomenon. To address this, we have measured the production of IFN-β in response to VBP and VBP plus LPS. While cell death in response to VBP does not begin until ~6–8 h post-VBP treatment (shown below, panel A), a LPS induced high levels of IFN-β release by 3 h post-treatment (shown below, panels B). confirming that the IFN-β production occurs before the cell death would be initiated,

providing time for IFN to inhibit cell death. We have included these results as Supplemental Figure 5A–B in our revised manuscript.

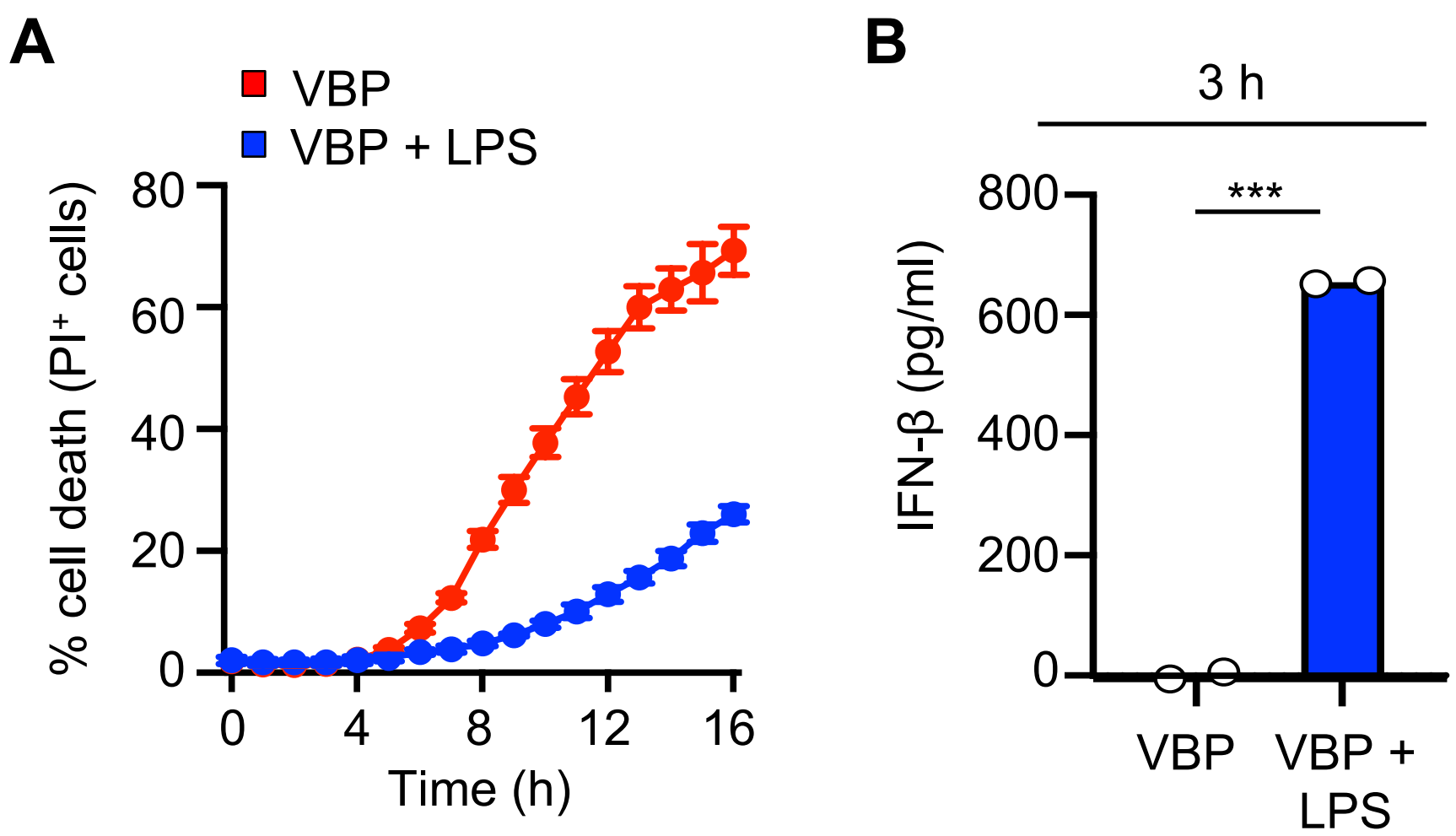

To further confirm that type I IFN production is not a parallel mechanism, we focused on the TLR4-TRIF-IRF3 axis that we identified as being critical for LPS-mediated inhibition of cell death. We found that IFN-β expression was blocked in cells that are deficient in IRF3, one of the critical components of this signaling axis (shown below). This suggest that IFN-β is produced through the TLR4-TRIF-IRF3 axis downstream of LPS to provide protection from cell death. We have included these results as Figure 3C in our revised manuscript.

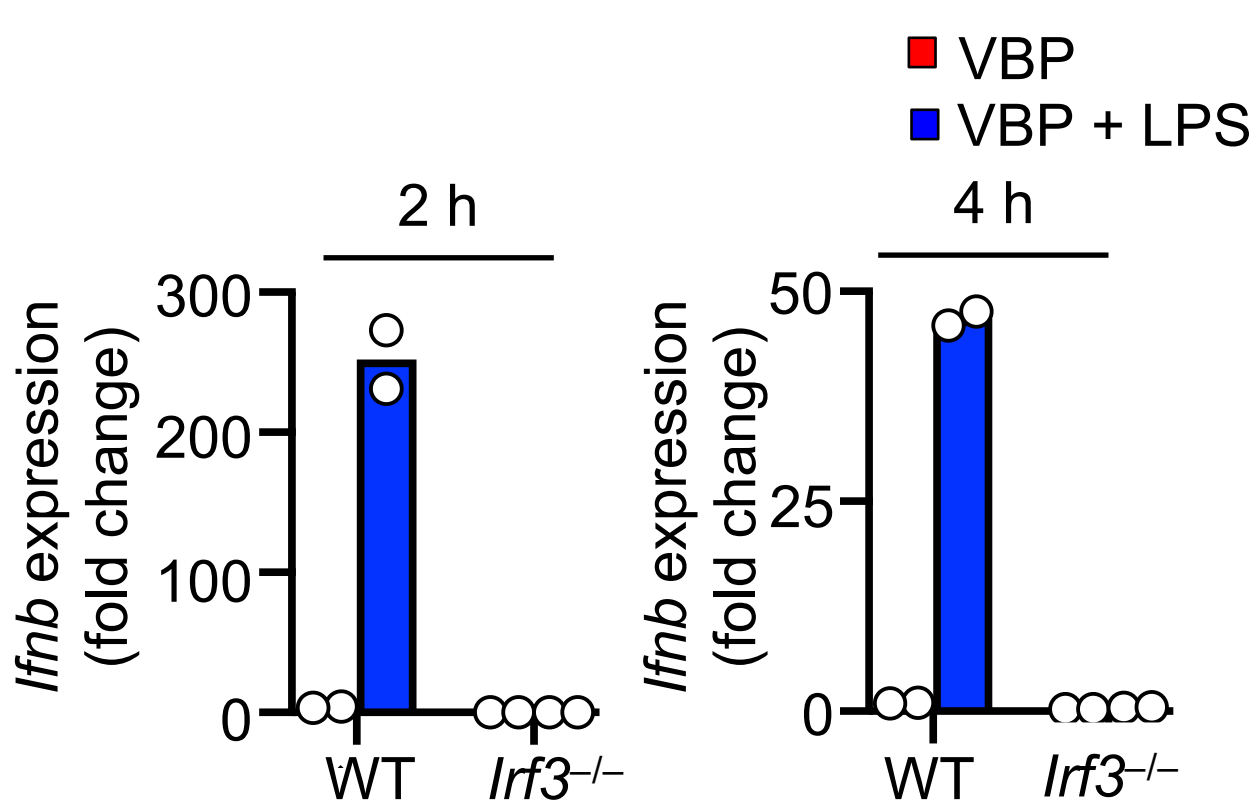

Moreover, we also assessed whether the addition of type I IFN would inhibit cell death in TLR4- or IFNAR1-deficient BMDMs. We found that IFN-α and IFN-β both inhibit cell death in TLR4-deficient BMDMs, but not in IFNAR1-deficient BMDMs (shown below). These results are shown in Figure 4A–B in our revised manuscript.

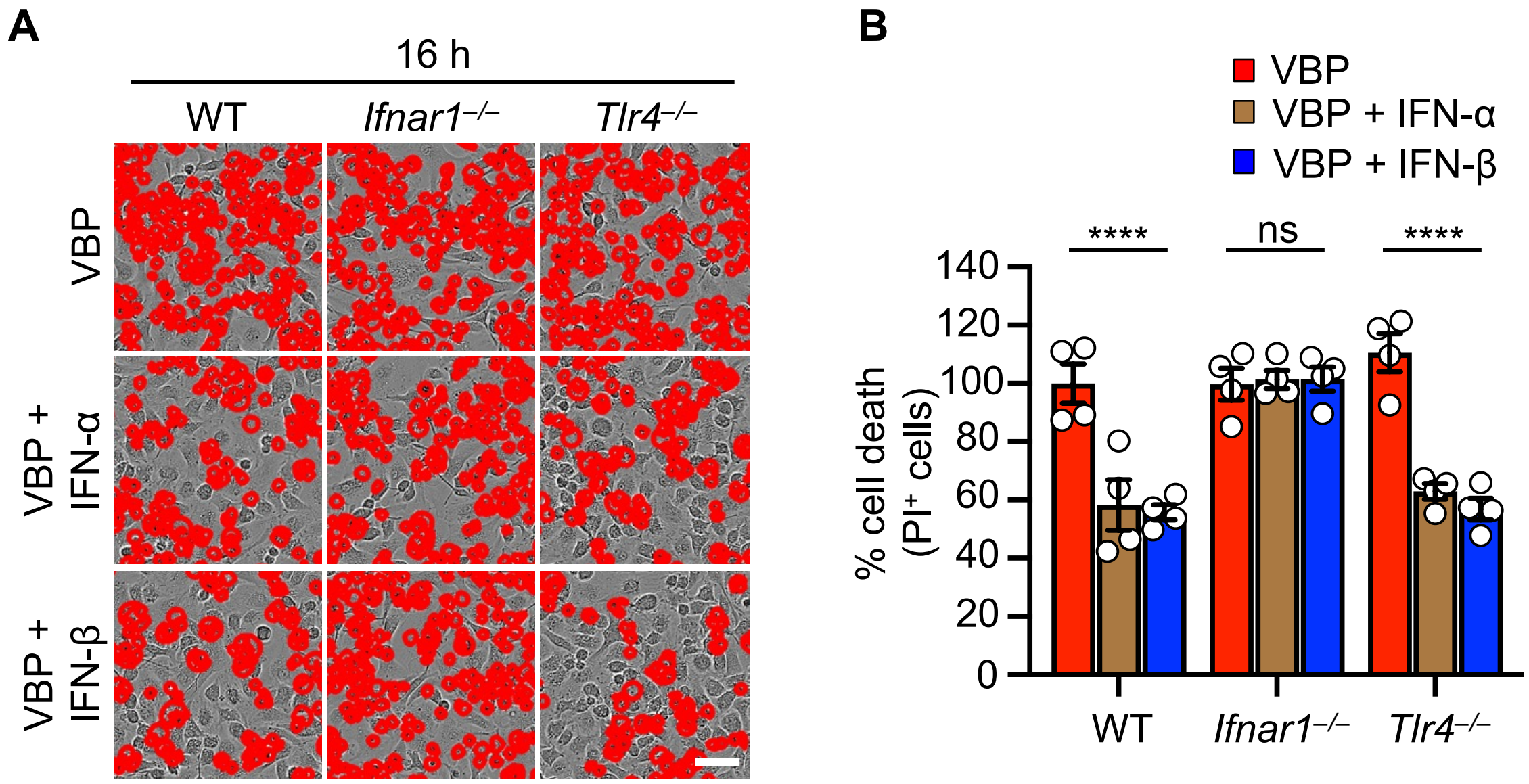

Together, these data suggest that LPS induces TLR4-TRIF-IRF3–mediated type I IFN production, and the subsequent IFN signaling regulates *NLRP1* transcription to inhibit NLRP1-mediated cell death.

3. Experimental Design - Priming Strategy
There is general confusion about the experimental approach, particularly the concurrent addition of Val-boroPro and LPS or IFNs. This design appears to conflict with the hypothesis that transcriptional downregulation mediates the observed inhibition, as pre-stimulation might be more appropriate to allow for transcriptomic changes. The authors should provide a clearer rationale for this choice or consider revising their protocol accordingly.

Here, the advisor is requesting clarification regarding our treatment protocol. In the current study, we have used concurrent treatment with VBP and LPS or IFNs, as we felt this would be the most therapeutically relevant dosing strategy. Using this treatment strategy, we found that concurrent addition of LPS or IFNs with VBP could inhibit VBP-mediated cell death. We would like to note that similar results can also be observed when cells are pre-treated (aka, primed) with LPS (shown below). We have included these results as Supplemental Figure 1A–B in our revised manuscript.

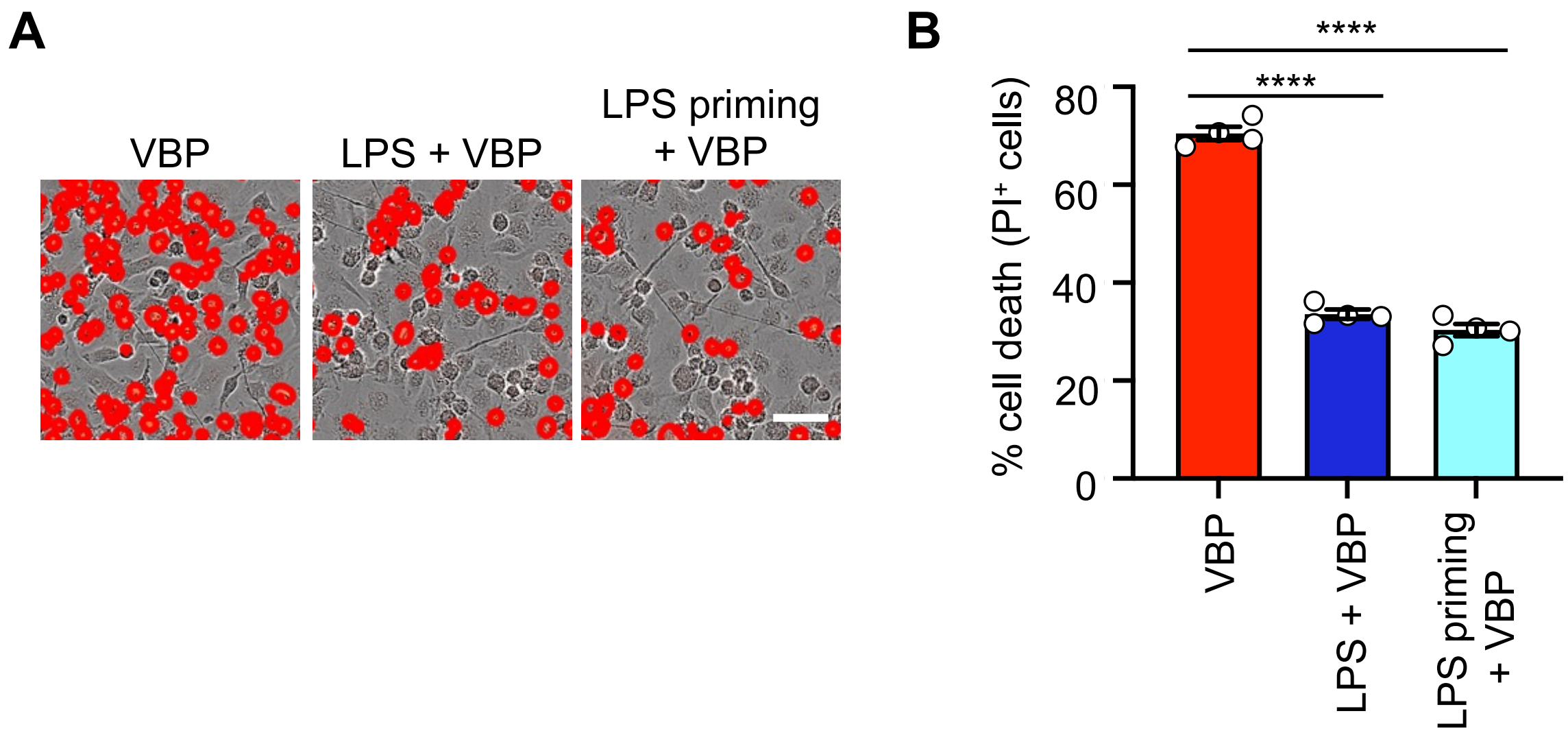

Moreover, we have measured the production of IFN-β in response to LPS. While cell death does not begin until ~6–8 h post-VBP treatment (shown below, panel A), VBP plus LPS-stimulated BMDMs produce and release high levels of IFN-β by 3 h post-treatment (shown below, panel B). We have included these results as Supplemental Figure 5A–B in our revised manuscript.

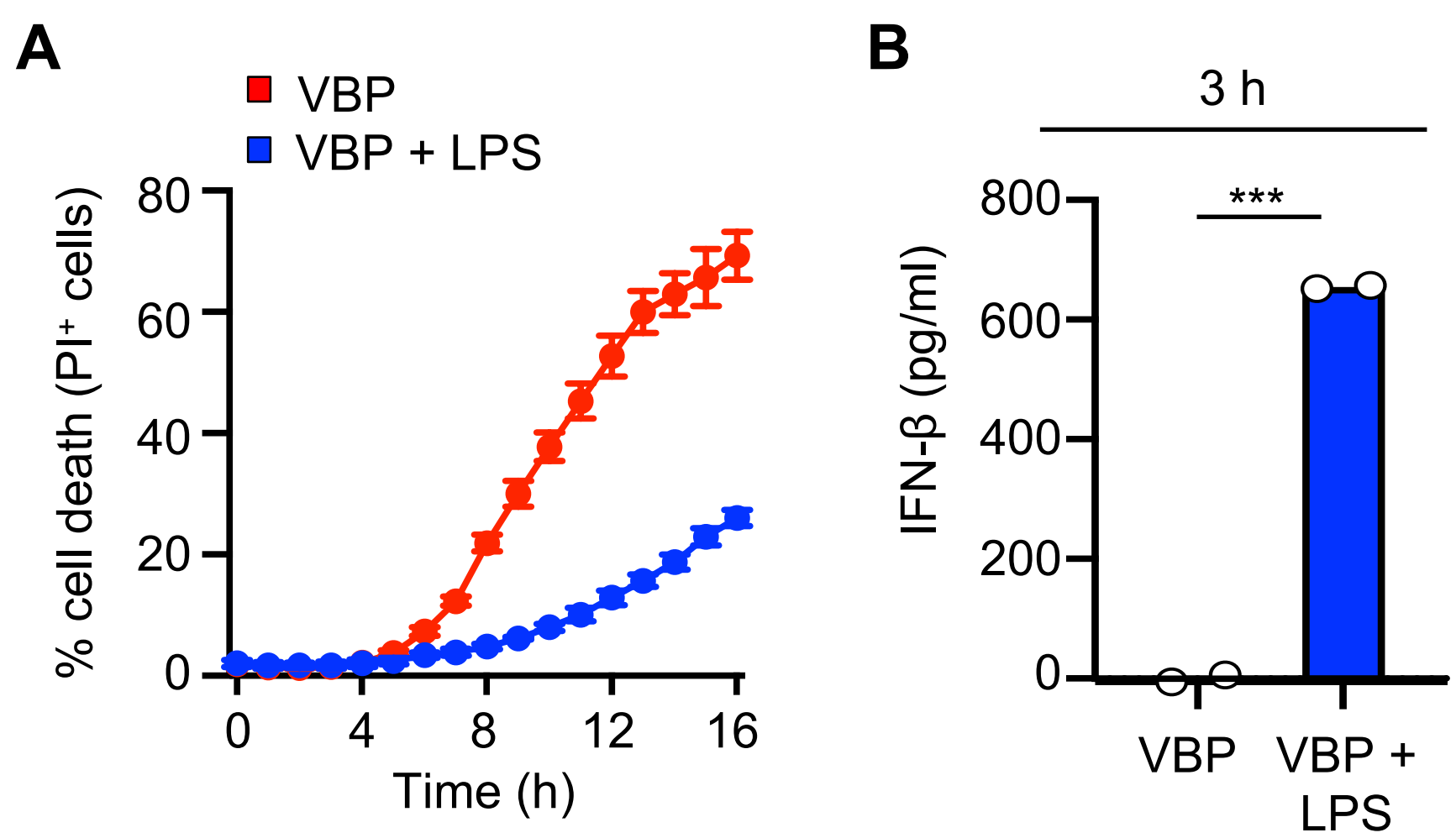

These kinetics provide enough time for concurrent treatment with LPS to induce IFN production and modulate NLRP1 expression levels, leading to the inhibition of cell death. Therefore, our experimental strategy is not in conflict with our hypothesis of transcriptional regulation.

4. Clinical Relevance and Human Data
Given the known species differences between human and mouse NLRP1, the inclusion of more human data, particularly with clinically relevant NLRP1 variants, would significantly enhance the manuscript's impact. This could involve assessing whether LPS or Type I IFNs modulate the

activation of disease-associated human NLRP1 variants or employing more physiologically relevant triggers, such as ribotoxic stress or pathogen infection.

Here, the advisor is requesting additional analyses in human cells, due to the differences between human and mouse NLRP1. To address this, we have now obtained multiple lines of evidence to confirm that a similar mechanism exists in human cells.

1. **First, we confirmed that NLRP1-dependent cell death occurs in human cells in response to VBP. We silenced *NLRP1* in human macrophages and found that VBP induced NLRP1-dependent cell death in these cells (shown below). We have included these results as Supplemental Figure 6 in our revised manuscript.**

Figure for referee with unpublished information has been removed upon request by the authors.

2. **Next, we confirmed that type I IFNs could block NLRP1-mediated cell death in human cells, similar to what we had observed in murine cells. We found that both human monocytes and macrophages undergo death in response to VBP that can be blocked with the addition of IFN-β (shown below). We have included these results as Figure 4E–F in our revised manuscript.**

Figure for referee with unpublished information has been removed upon request by the authors.

3. To provide further confirmation that type I IFNs can inhibit NLRP1-mediated cell death in human cells, we also tested whether IFN-α could block VBP-induced cell death. Indeed, we found that IFN-α significantly inhibited VBP-induced cell death in both human macrophages and monocytes (shown below), similar to what we observed in murine cells. We have included these results as Figure 4C–D in our revised manuscript.

Figure for referee with unpublished information has been removed upon request by the authors.

4. To confirm that the underlying regulatory mechanism was shared between murine and human cells, we further assessed the transcriptional regulation of NLRP1 in human cells. We found that the addition of LPS to induce type I IFN production reduced the ***NLRP1*** mRNA expression in human monocytes and macrophages (shown below). We have included these results as Figure 5E–F in our revised manuscript.

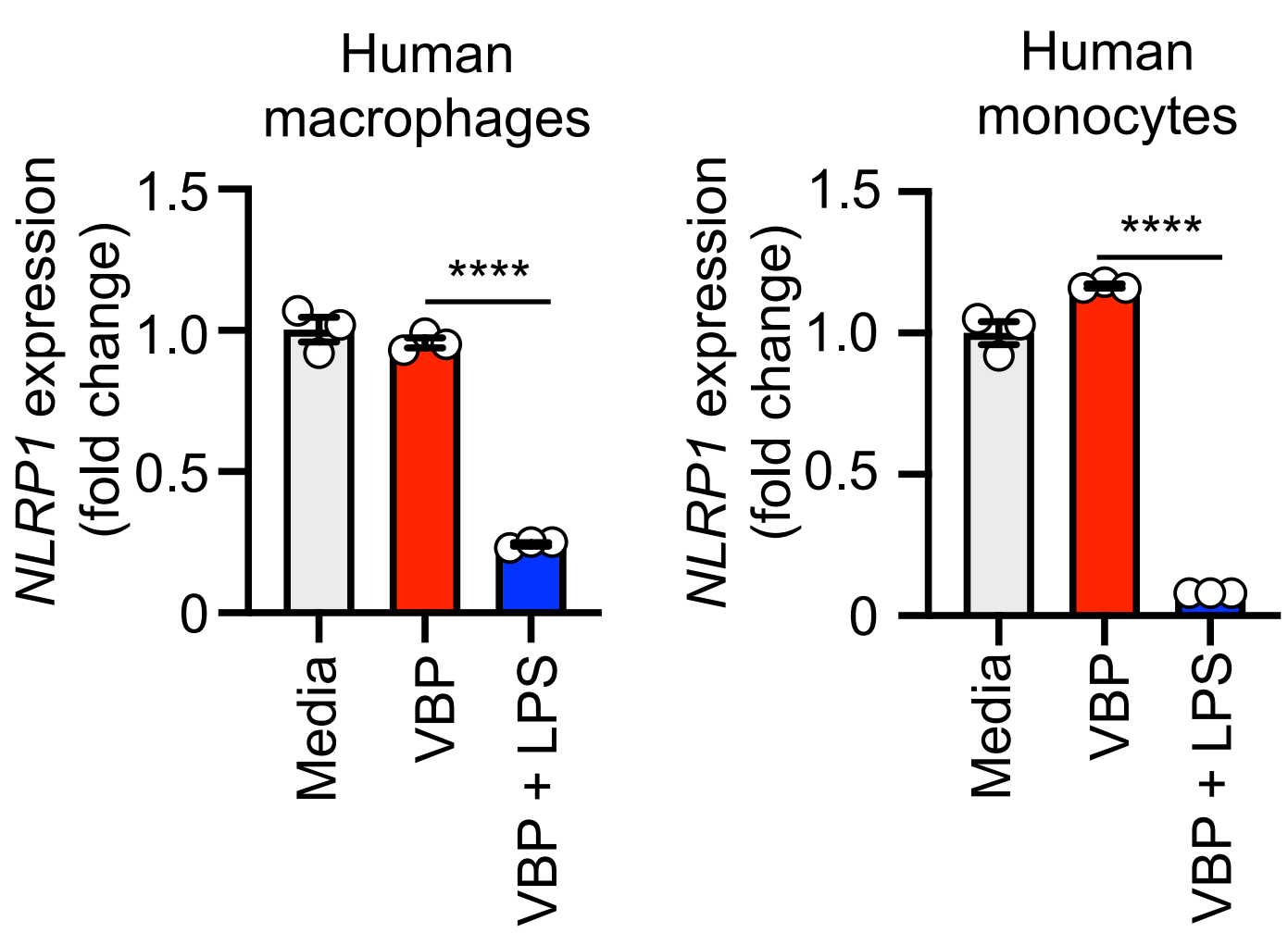

5. We also found that IFN-β treatment reduced the expression of ***NLRP1*** mRNA (shown below). We have included these results as Figure 5A in our revised manuscript.

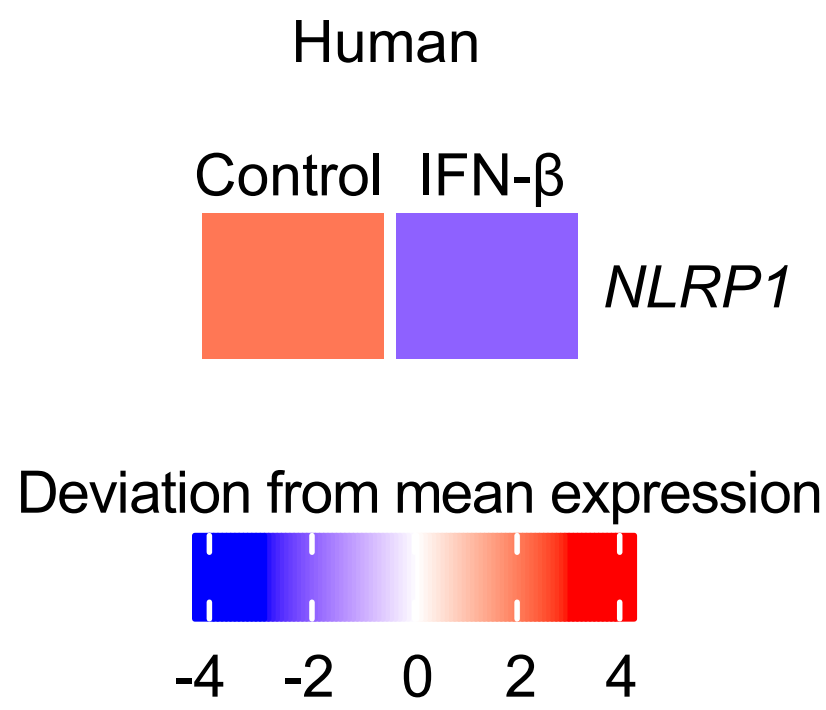

Together, these data confirm that treatment with LPS to induce type I IFN production, or treatment with type I IFNs directly, leads to a downregulation of *NLRP1* to prevent cell death in both murine and human cells.

While we appreciate the suggestion to use additional NLRP1 triggers, such as ribotoxic stress or UVB, these triggers induce the activation of NLRP1 in non-myeloid cells, rather than myeloid cells. Given our focus on the regulation of NLRP1-mediated cell death in myeloid cells, based on their importance in innate immunity and inflammation, we feel the use of these triggers is out of scope.

Regarding the relevance of our findings to clinically relevant NLRP1 variants, IFN-β is a treatment for multiple sclerosis (PMID: 32863784), a disease with a potentially causative NLRP1 variant (amino acid substitution from glycine to serine) (PMID: 28623311). Combined with our mechanistic evaluation showing that type I IFN treatment reduces *NLRP1* expression and inhibits NLRP1-mediated cell death, this suggests that type I IFN therapy may improve outcomes for patients with MS through its ability to inhibit NLRP1, as we discuss in the revised manuscript. Unfortunately, we do not have access to clinical samples from patients with these variants, and these are not readily available in the field. Therefore, we cannot do additional studies in this direction. Nevertheless, these clinical findings underscore the potential translational significance of our study and its relevance to human disease.

5. Therapeutic Potential

Finally, while the authors propose potential therapeutic applications, the evidence remains preliminary. More robust human data, as noted, along with a demonstration that this modulation could influence clinically relevant NLRP1-driven pathologies, would be critical for substantiating this claim.

Here, the advisor is requesting additional analyses in human cells to confirm the therapeutic potential of our study. To address this, we have now obtained multiple lines of evidence to confirm the relevance of type I IFN treatment to inhibit NLRP1 activation in human cells.

1. **First, we confirmed that NLRP1-dependent cell death occurs in human cells in response to VBP. We silenced *NLRP1* in human macrophages and found that VBP induced NLRP1-dependent cell death in these cells (shown below). We have included these results as Supplemental Figure 6 in our revised manuscript.**

Figure for referee with unpublished information has been removed upon request by the authors.

2. **Next, we confirmed that type I IFNs could block NLRP1-mediated cell death in human cells, similar to what we had observed in murine cells. We found that both human monocytes and macrophages undergo death in response to VBP that can be blocked with the addition of IFN-β (shown below). We have included these results as Figure 4E–F in our revised manuscript.**

Figure for referee with unpublished information has been removed upon request by the authors.

3. **To provide further confirmation that type I IFNs can inhibit NLRP1-mediated cell death in human cells, we also tested whether IFN-α could block VBP-induced cell death. Indeed, we found that IFN-α significantly inhibited VBP-induced cell death in both human macrophages and monocytes (shown below), similar to what we observed in murine cells. We have included these results as Figure 4C–D in our revised manuscript.**

Figure for referee with unpublished information has been removed upon request by the authors.

4. **To confirm that the underlying regulatory mechanism was shared between murine and human cells, we further assessed the transcriptional regulation of NLRP1 in human cells. We found that the addition of LPS to induce type I IFN production reduced the *NLRP1* mRNA expression in human monocytes and macrophages (shown below). We have included these results as Figure 5E–F in our revised manuscript.**

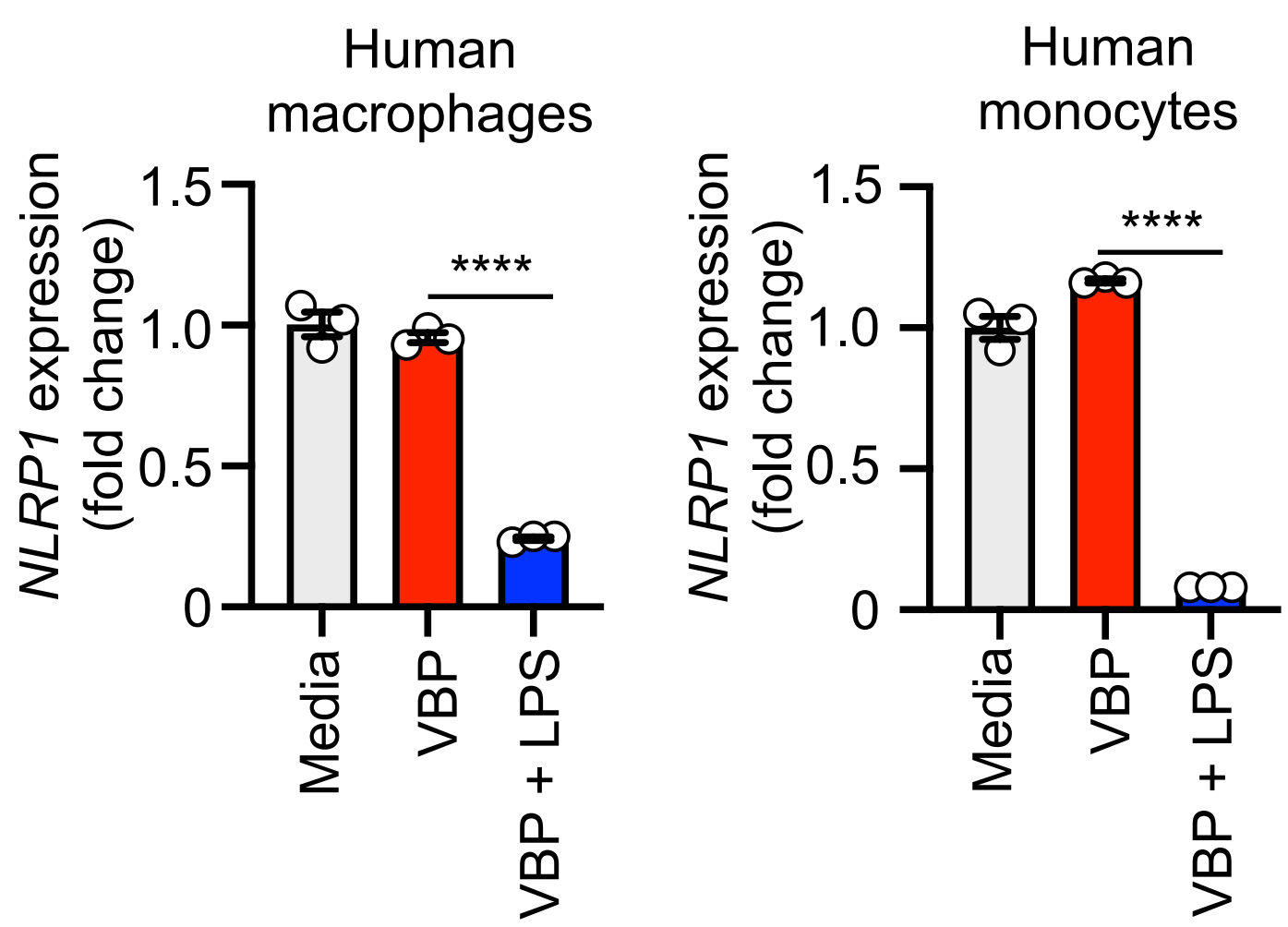

5. **We also found that IFN-β treatment reduced the expression of *NLRP1* mRNA (shown below). We have included these results as Figure 5A in our revised manuscript.**

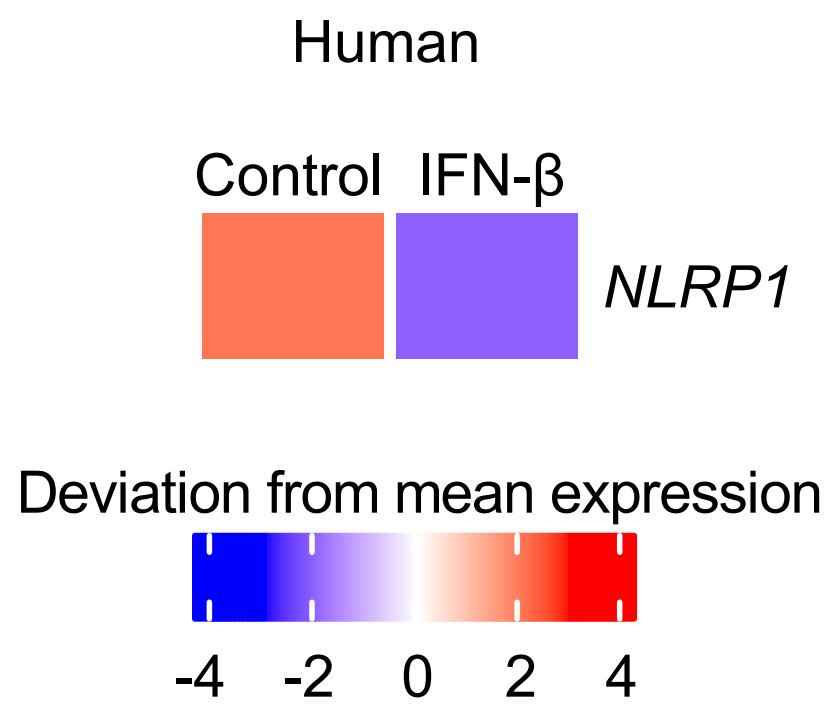

Together, these data confirm that treatment with LPS to induce type I IFN production, or treatment with type I IFNs directly, leads to a downregulation of *NLRP1* to prevent cell death in human cells.

Regarding the potential therapeutic relevance of our findings, there are currently no well-characterized in vivo models of disease exclusively driven by NLRP1, limiting our ability to perform pre-clinical evaluations of NLRP1-blocking treatments in disease models. However, as noted above, IFN-β is an established treatment for multiple sclerosis (PMID 32863784), a disease with a potentially causative NLRP1 variant (PMID: 28623311). Combined with our mechanistic evaluation showing that type I IFN treatment reduces *NLRP1* expression and inhibits NLRP1-mediated cell death, this suggests that type I IFN therapy may improve outcomes for patients with MS through its ability to inhibit NLRP1, as we discuss in our revised manuscript. The combination of our in vitro data in human cells and these clinical studies strongly supports the relevance of our findings to human disease and therapeutic applications.

Referee #1:

The authors clearly show that type I interferon (IFN I) inhibits NLRP1 activity in mouse and human myeloid cells. They begin by observing that LPS inhibits VBP-NLRP1-stimulated cytokine release and pyroptosis, and then using KO cells logically identify the means whereby LPS does this as via TRIF-IRF3-IFN I. They then show that direct treatment of cells with IFN I inhibits NLRP1, likely due to inhibition of NLRP1 expression.

Overall this is an interesting and robust observation, however the contribution to new knowledge is very incremental. Essentially the paper shows that in myeloid cells IFN I inhibits NLRP1 activity. There is no mechanistic advance, in terms of a significant increase in our understanding of how NLRP1 is regulated, and no physiological context for IFN I regulation of NLRP1.

We thank the reviewer for highlighting that our study contains "<u>interesting and robust observation</u>".

We would like to re-iterate the key advances made by our study, including those added during the revision stage. Our study shows (1) the surprising observation that LPS, the canonical activator of innate immunity, inhibits NLRP1-dependent inflammatory cell death, and that this cell death is PANoptosis; (2) the finding that IFN-β is responsible for this effect via signaling through TLR4, TRIF, and IRF3; and (3) the overall human relevance, suggesting a potential mechanistic basis to explain and expand IFN-β treatment for NLRP1-mediated inflammatory disease.

In addition to the novelty, the reviewer is also asking for two key additions to the study: 1) additional mechanistic insight into how NLRP1-mediated cell death is regulated, and 2) additional physiological context for type I IFN regulating NLRP1. We have addressed these point-by-point below.

Mechanistic insight:

1) **To provide additional mechanistic insight into how NLRP1-mediated cell death is regulated, we first determined whether type I IFN treatment affected NLRP1 expression. Using publicly available datasets for human and murine cells, we found that *NLRP1* mRNA levels were downregulated in response to IFN-β treatment (shown below). We have included these results as Figure 5A–B in our revised manuscript.**

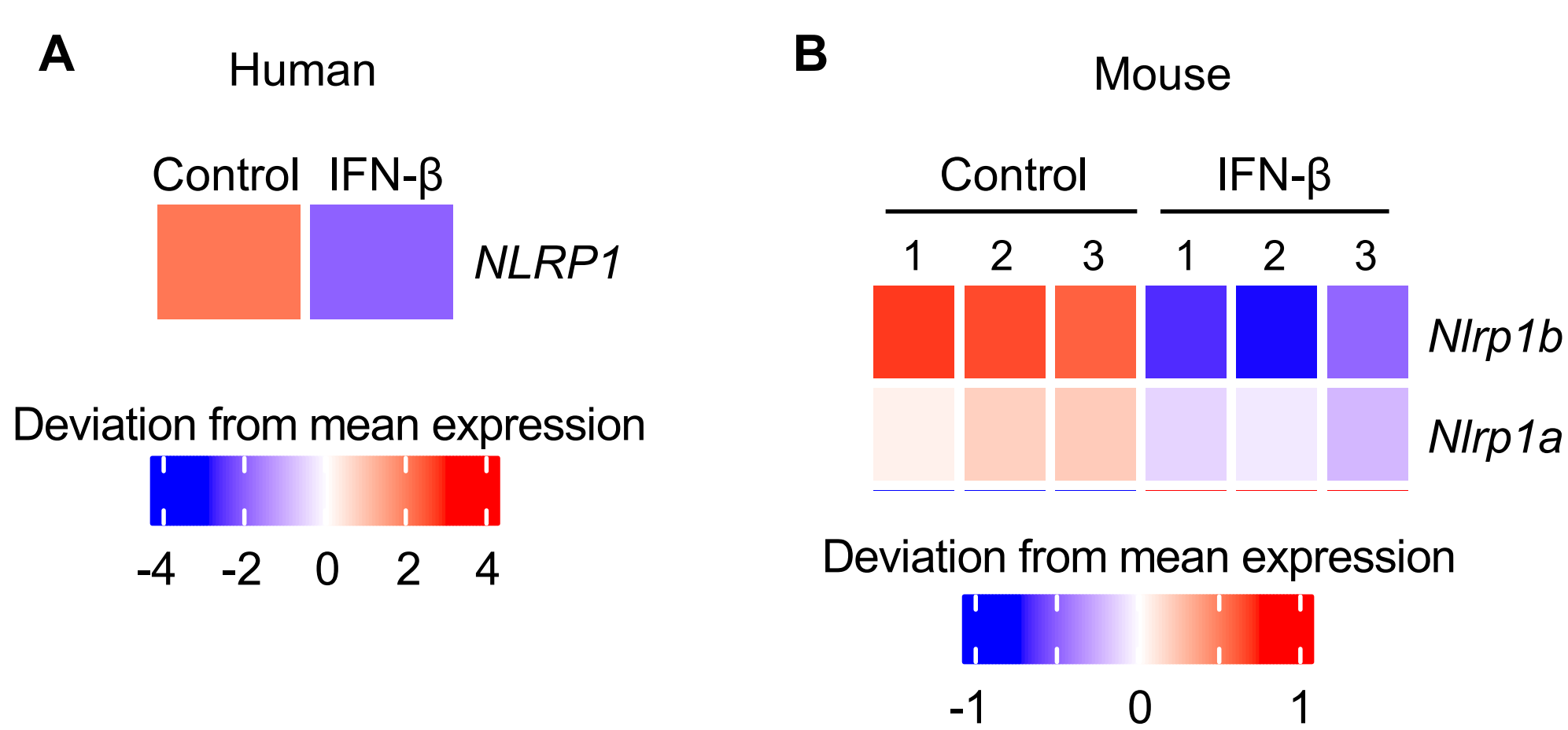

2) **To validate these expression data, we then measured *NLRP1* mRNA levels in murine BMDMs and human macrophages and monocytes, and we found that treatment with LPS or type I IFN significantly downregulated *NLRP1* mRNA expression. Specifically, we found a significant decrease in *NLRP1* mRNA expression in both mouse and human macrophages and monocytes in response to LPS or IFN treatment when the cells were treated with VBP (shown below). These results suggest that NLRP1 is regulated at the transcriptional level. We have included these results as Figure 5C–E in our revised manuscript.**

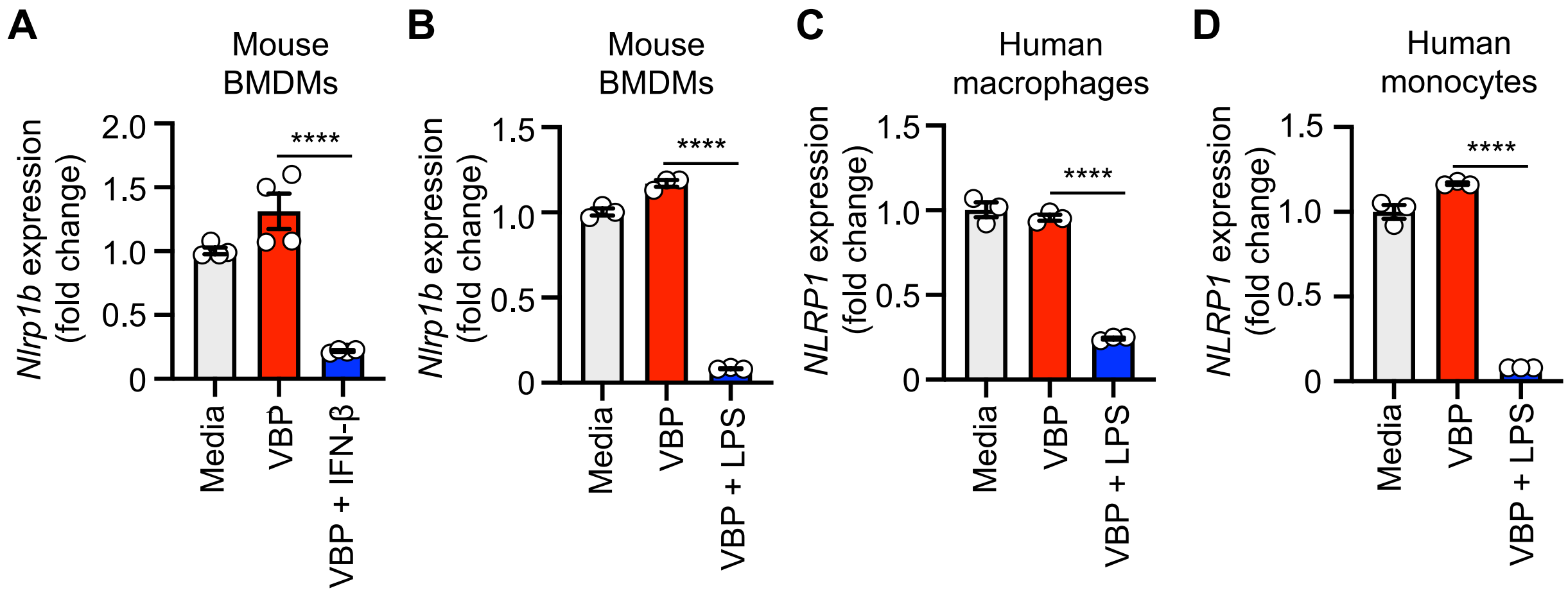

3) **We also confirmed that type I IFN production occurs via the TLR4-TRIF-IRF3 axis that we identified as being critical for LPS-mediated inhibition of cell death. We found that IFN-β expression was blocked in cells that are deficient in IRF3, one of the critical components of this signaling axis (shown below). This suggest that IFN-β is produced through the TLR4-TRIF-IRF3 axis downstream of LPS to provide protection from cell death. We have included these results as Figure 3C in our revised manuscript.**

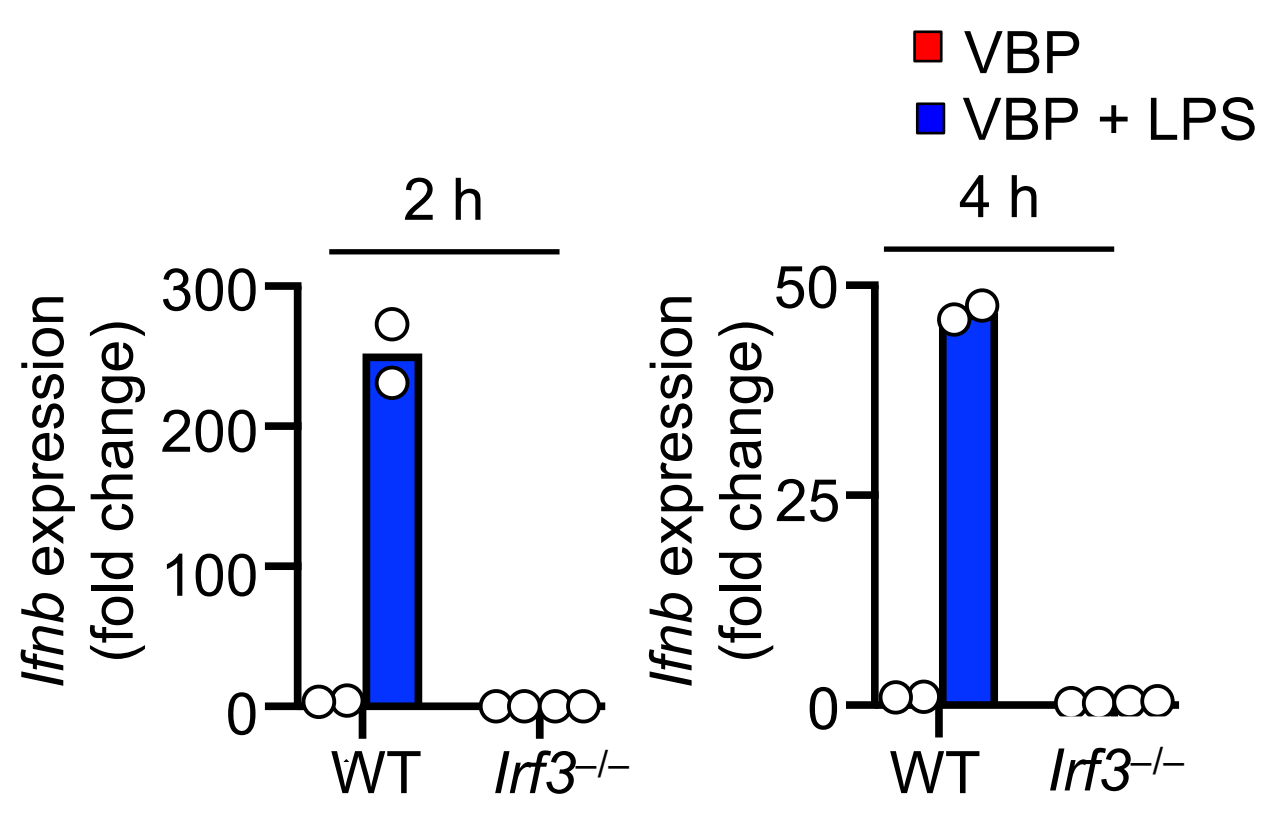

4) **We also assessed whether the addition of type I IFN would inhibit cell death in TLR4- or IFNAR1-deficient BMDMs. We found that IFN-α and IFN-β both inhibit cell death in TLR4-deficient BMDMs, but not in IFNAR1-deficient BMDMs (shown below). We have included these results as Figure 4A–B in our revised manuscript.**

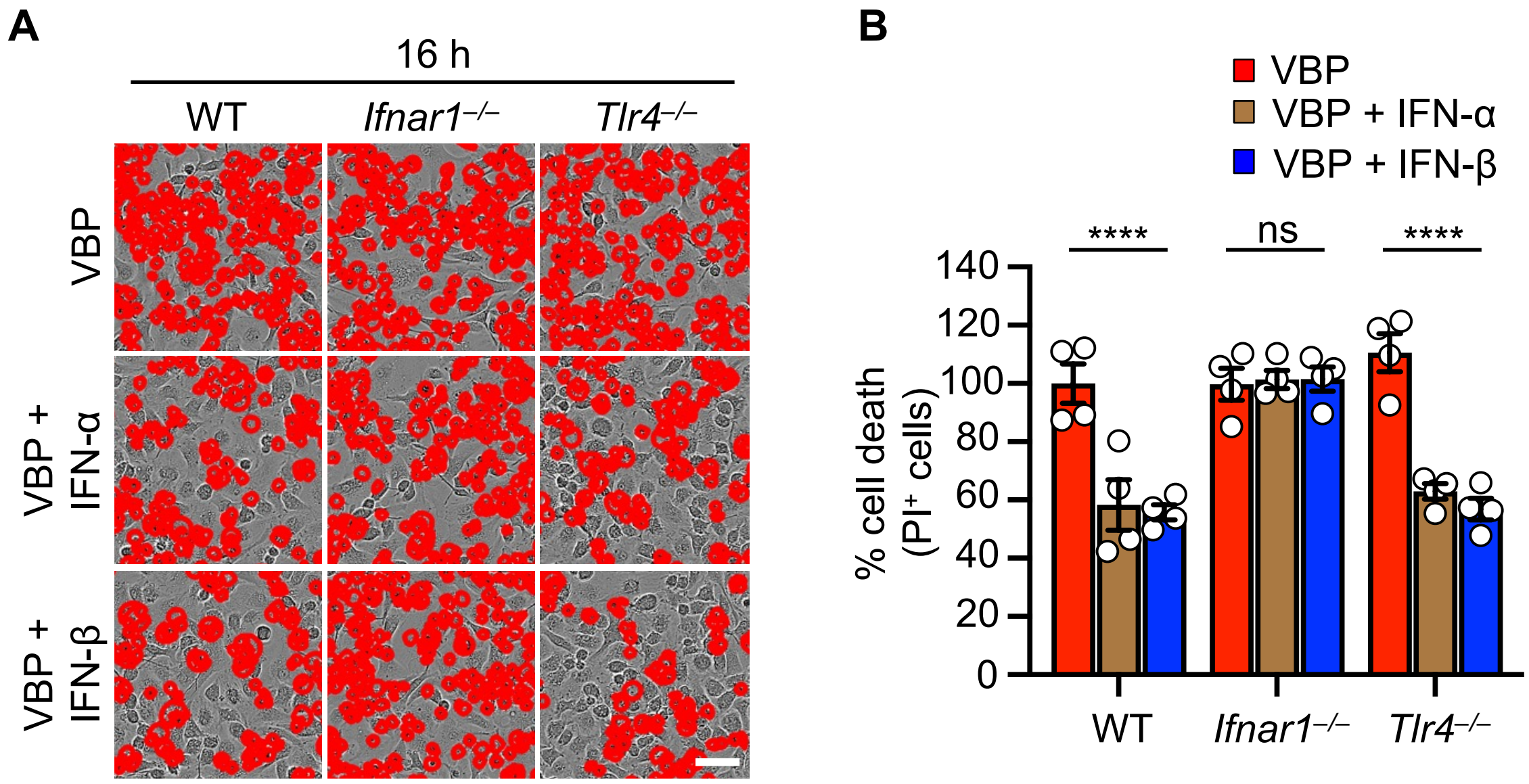

Together, these data suggest that LPS induces TLR4-TRIF-IRF3–mediated type I IFN production, and the subsequent IFN signaling regulates *NLRP1* transcription to inhibit NLRP1-mediated cell death.

Physiological context:
To provide additional physiological context for type I IFN regulating NLRP1, we have now obtained multiple lines of evidence to confirm that the mechanism we identified in murine cells also occurs in human cells, and that there is physiological relevance for this pathway in disease.

1) **We used *NLRP1* silencing to confirm that NLRP1-dependent cell death occurs in human cells in response to VBP (shown below). We have included these results as Supplemental Figure 6 in our revised manuscript.**

Figure for referee with unpublished information has been removed upon request by the authors.

2) Additionally, we confirmed that type I IFNs could block NLRP1-mediated cell death in human cells, similar to what we had observed in murine cells. We found that both human monocytes and macrophages undergo death in response to VBP that can be blocked with the addition of IFN-β (shown below). We have included these results as Figure 4E–F in our revised manuscript.

Figure for referee with unpublished information has been removed upon request by the authors.

3) We also found that IFN-α could block VBP-induced cell death in both human macrophages and monocytes (shown below), similar to our observations in murine cells. We have included these results as Figure 4C–D in our revised manuscript.

Figure for referee with unpublished information has been removed upon request by the authors.

4) To confirm that the underlying regulatory mechanism was conserved between mouse and human, we further assessed the transcriptional regulation of NLRP1 in human cells. We found that the addition of LPS to induce type I IFN production reduced the *NLRP1* mRNA expression in human monocytes and macrophages (shown below). We have included these results as Figure 5E–F in our revised manuscript.

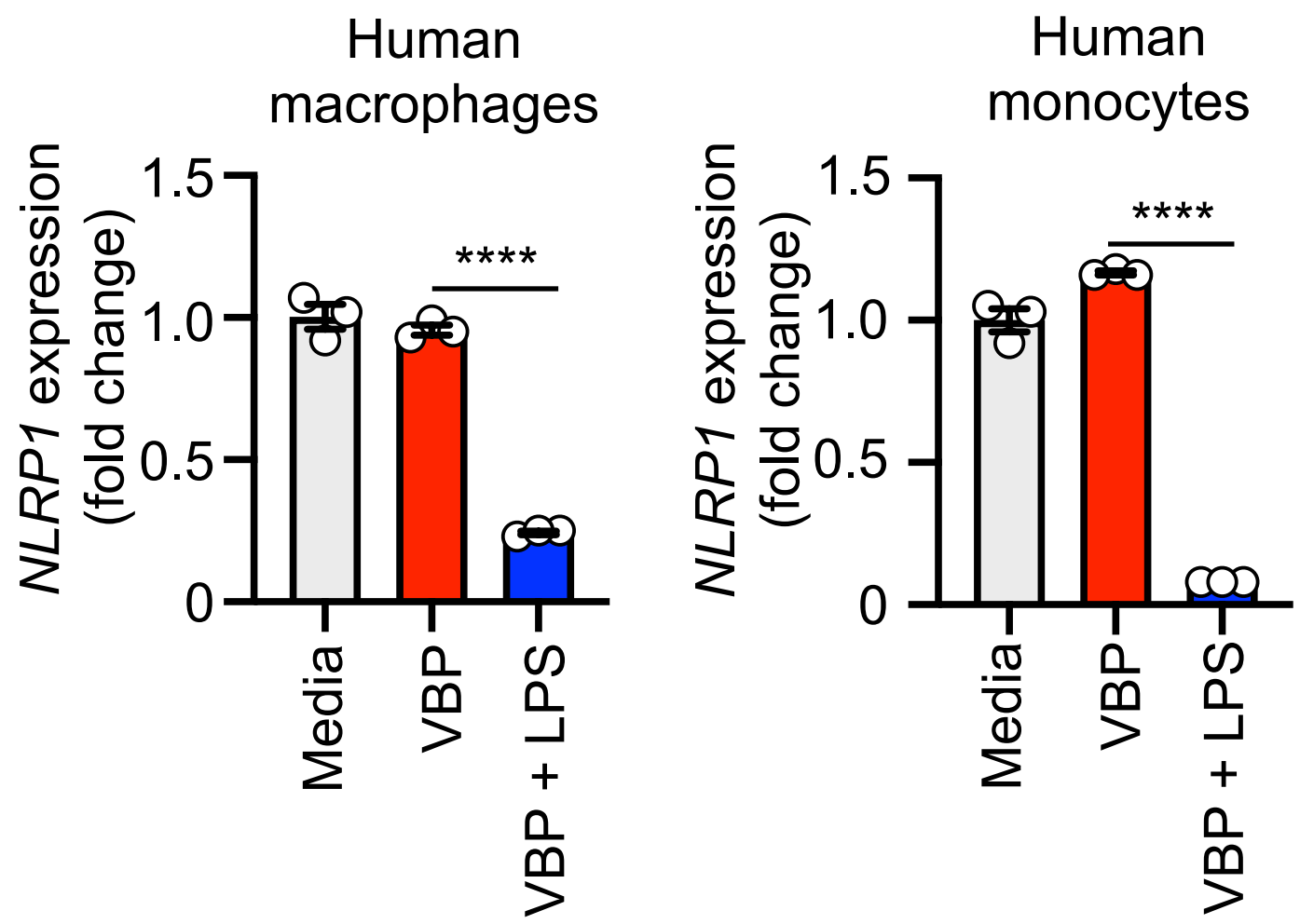

5) **We also found that IFN-β treatment reduced the expression of *NLRP1* mRNA (shown below). We have included these results as Figure 5A in our revised manuscript. Together, these data confirm that treatment with LPS to induce type I IFN production leads to a downregulation of *NLRP1* to prevent cell death in both murine and human cells.**

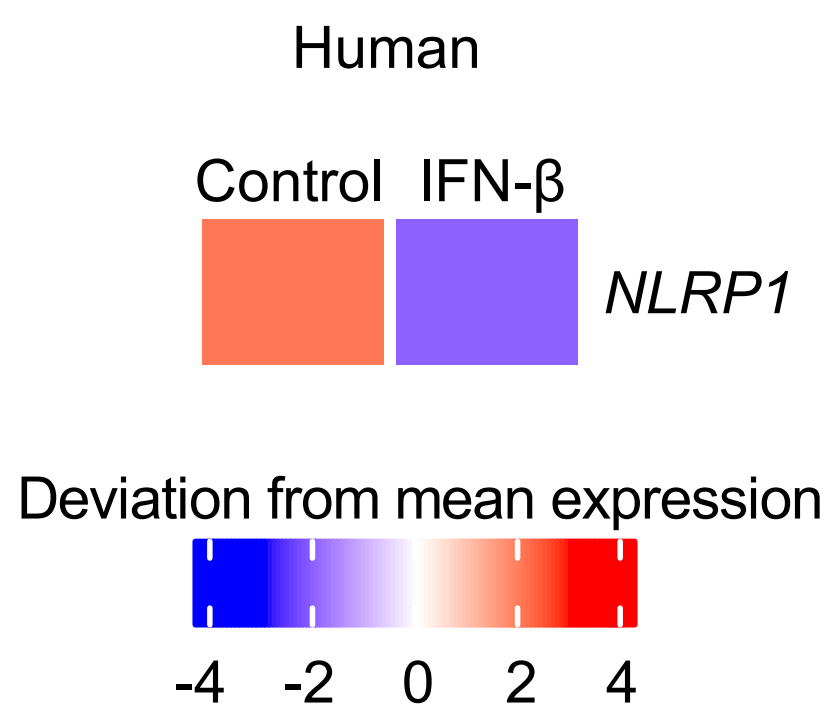

6) **Physiologically, in human patients, IFN-β is an established treatment for multiple sclerosis (PMID: 32863784), a disease with a potentially causative NLRP1 variant (amino acid substitution from glycine to serine) (PMID: 28623311). Combined with our mechanistic evaluation showing that type I IFN treatment reduces *NLRP1* expression and inhibits NLRP1-mediated cell death, this suggests that type I IFN therapy may improve outcomes for patients with MS through its ability to inhibit NLRP1, as we discuss in the revised manuscript. These clinical findings underscore the potential translational significance of our study and its relevance to human disease.**

Referee #2:

Concisely written manuscript reporting that the TRIF-IRIF3 pathway inhibits NLRP1 inhibition in murine and human cells.

Major concerns: I appreciate the concise message of the paper, but I'd like to leave it to the editor to assess the novelty . The author proposes that type I interferon be used as a potential inhibitor for NLRP1-driven inflammation. This is a bit of a stretch,. My opinion is that the antiviral effect of type I interferon (through ISGs etc) likely far outweighs NLRP1 inhibition in a therapeutic context. I am also not aware of any prior examples of using one immunostimulatory cytokine to treat inflammatory disorders caused by another immunostimulatory cytokine.

We thank the reviewer for noting that our study is "concise" and "reporting that the TRIF-IRF3 pathway inhibits NLRP1 in both murine and human cells".

Here, the reviewer is requesting additional insight into the therapeutic potential of using type I IFNs to treat inflammatory disorders. In addition to the well-established, potent antiviral effects of type I IFNs, there are also many studies reporting their anti-inflammatory properties. From a therapeutic perspective, type I IFNs are already in clinical use for treating inflammatory diseases. As a key example, IFN-β is an established treatment for multiple sclerosis (PMID: 32863784), a disease with a potentially causative NLRP1 variant (amino acid substitution from glycine to serine) (PMID: 28623311). Combined with our mechanistic evaluation showing that type I IFN treatment reduces *NLRP1* expression and inhibits NLRP1-mediated cell death, this suggests that type I IFN therapy may improve outcomes for patients with MS through its ability to inhibit NLRP1, as we discuss in the revised manuscript. These clinical findings underscore the potential translational significance of our study and its relevance to human disease.

The authors propose NLRP1 mRNA downregulation as the mechanism explaining why IRF3/type I interferon can downregulate NLRP1. This part of the findings seems a little underdeveloped. In Fig 4E, some sort of absolute quantitation of the NLRP1 transcript and NLRP1 protein is needed.

Here, the reviewer is requesting additional mechanistic insight into how LPS and type I IFNs downregulate NLRP1. To address this, we first determined whether type I IFN treatment affected NLRP1 expression. Using publicly available datasets for human and murine cells, we found that *NLRP1* mRNA levels were downregulated in response to IFN-β treatment (shown below). We have included these results as Figure 5A–B in our revised manuscript.

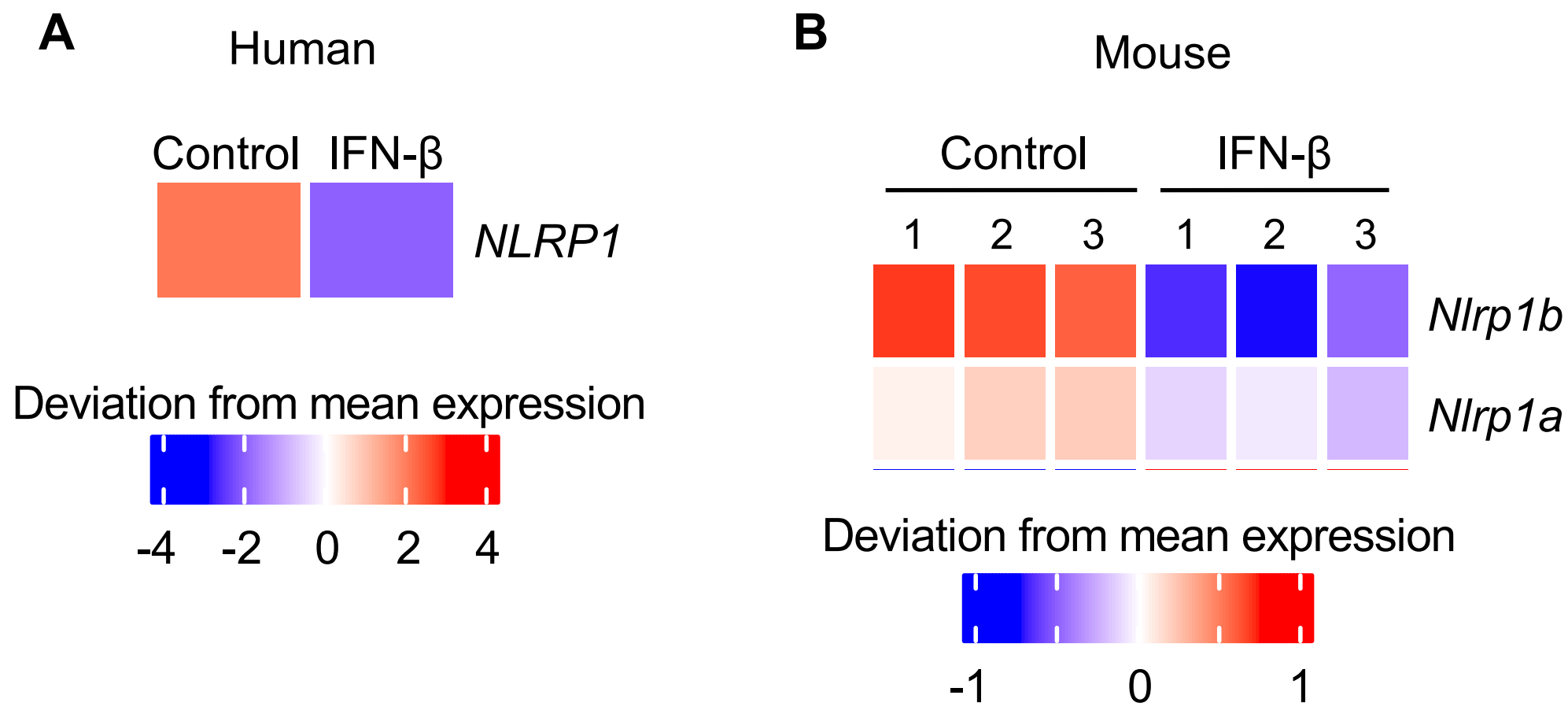

To validate these expression data, we then measured *NLRP1* mRNA levels in murine BMDMs and human macrophages and monocytes, and we found that treatment with LPS or type I IFN significantly downregulated *NLRP1* mRNA expression. Specifically, we found a significant decrease in *NLRP1* mRNA expression in both mouse and human macrophages and monocytes in response to LPS or IFN treatment when the cells were treated with VBP (shown below). These results suggest that NLRP1 is regulated at the transcriptional level. We have included these results as Figure 5C–E in our revised manuscript.

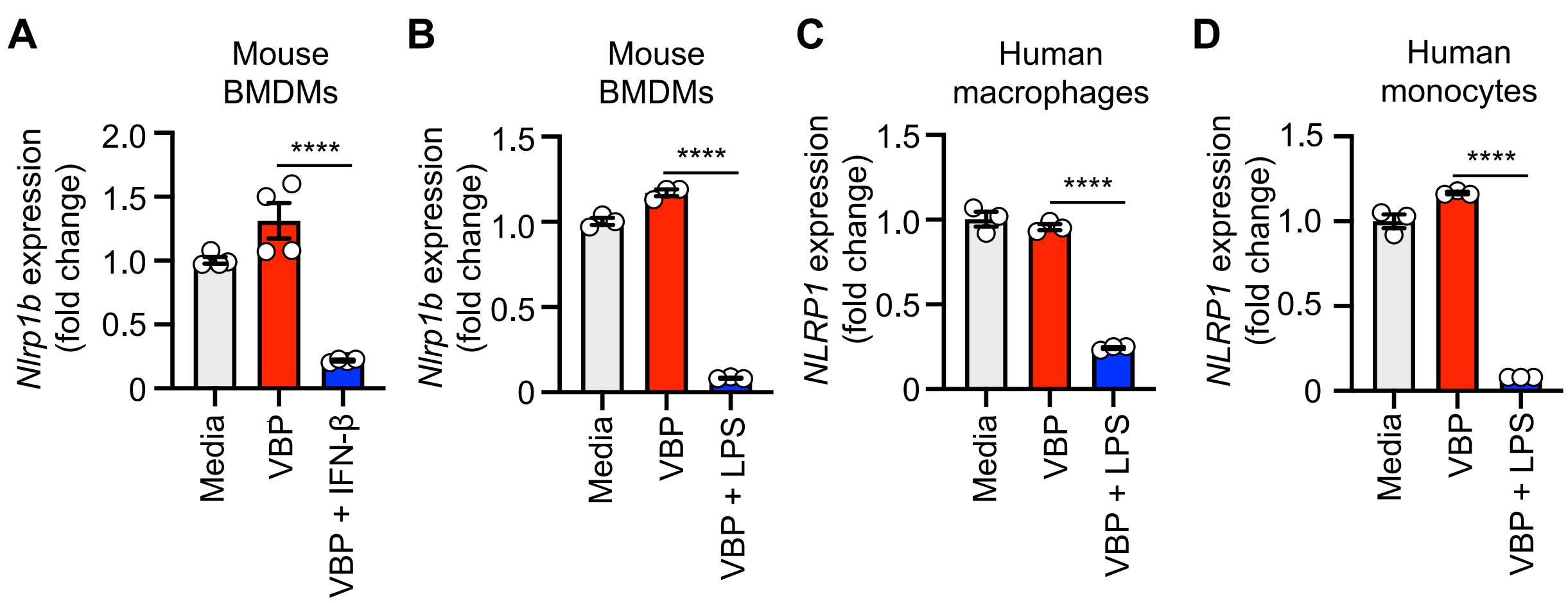

Together, the RNAseq data and our independent transcriptional analysis shows that LPS-mediated production of type I IFNs, or direct treatment with IFN-β, regulates the expression of *NLRP1* to control NLRP1-mediated cell death.

Minor concern: I am under the impression that CARD8 is the predominant sensor that drives VBP-induced pyroptosis in human macrophages. This has an impact on the findings in FIgure 4C-D. The authors should address this.

To confirm that NLRP1 is the predominant sensor involved in VBP-induced cell death in human macrophages, we used siRNA to silence *NLRP1* in these cells. Silencing *NLRP1* significantly reduced the of cell death, confirming that VBP induces NLRP1-dependent cell death in human cells (shown below). We have included these results as Supplemental Figure 6 in our revised manuscript. While CARD8 may also be involved, our results show that using VBP as a trigger to assess NLRP1-mediated cell death in human cells is appropriate.

Figure for referee with unpublished information has been removed upon request by the authors.

Referee #3:

In their manuscript, B. Sharma and colleagues investigate the molecular mechanisms regulating NLRP1 inflammasome activation, demonstrating that LPS-induced inhibition of NLRP1 is mediated by type I interferon. This work sheds light on the interplay between inflammatory cytokine production and inflammasome regulation in the context of inflammatory responses.

The study presents solid mechanistic insights; however, it offers limited conceptual novelty, which constitutes the primary drawback. For instance, in Figure 4E, the authors show the transcriptional regulation of various NLRs by interferon, yet this finding receives minimal attention in the text. It would be valuable for the authors to comment on the differential regulation of specific NLRs, as this may provide a broader perspective on the biological implications of their data.

We thank the reviewer for highlighting that our study "sheds light on the interplay between inflammatory cytokine production and inflammasome regulation in the context of inflammatory responses", and for highlighting that "the mechanistic advances presented in this manuscript are appreciated and contribute meaningfully to the field".

We would like to re-iterate the key novel aspects of our study, including those added during the revision stage. Our study shows (1) the surprising observation that LPS, the canonical activator of innate immunity, inhibits NLRP1-dependent inflammatory cell death, and that this cell death is PANoptosis; (2) the finding that IFN-β is responsible for this effect via signaling through TLR4, TRIF, and IRF3; and (3) the overall human relevance, suggesting a potential mechanistic basis to explain and expand IFN-β treatment for NLRP1-mediated inflammatory disease.

In addition to the novelty, the reviewer is also requesting additional follow-up on the observed IFN-mediated regulation of NLRs in our study. Given our focus on NLRP1 here, we validated our findings by assessing the effect of type I IFNs on NLRP1 expression. We found that LPS-mediated type I IFN production, or direct treatment with IFN-β, reduced *NLRP1* expression in murine and human cells (data shown below). We have included these results as Figure 5C–E in our revised manuscript.

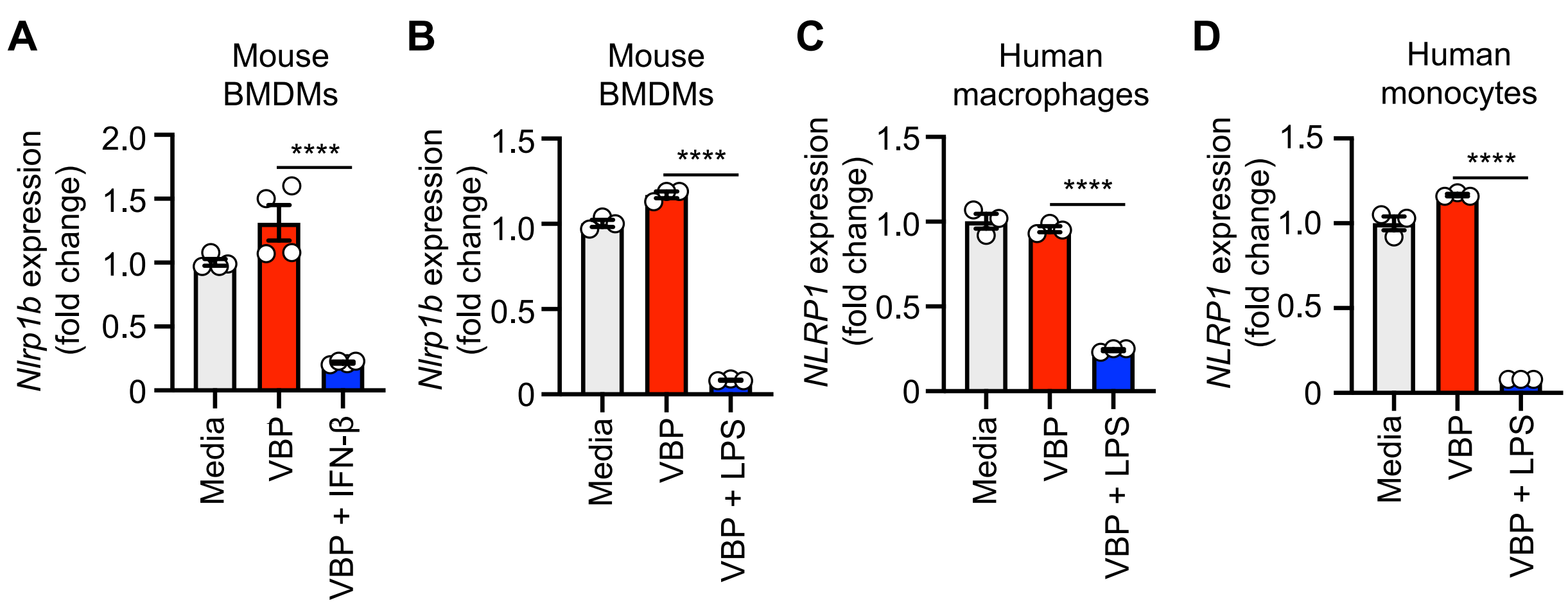

To more broadly assess the differential regulation of NLRs by IFN-β, we also used publicly available gene expression datasets that include IFN-β–treated human and murine

macrophages. We found that *NLRP1* mRNA levels are downregulated in response to IFN-β treatment, while most other NLR family genes were upregulated (shown below). This offers clues into the unexpected effect of LPS in NLRP1-mediated cell death, and has potential implications for treatment of NLRP1-mediated disease. We have included these results as Figure 5B of our revised manuscript.

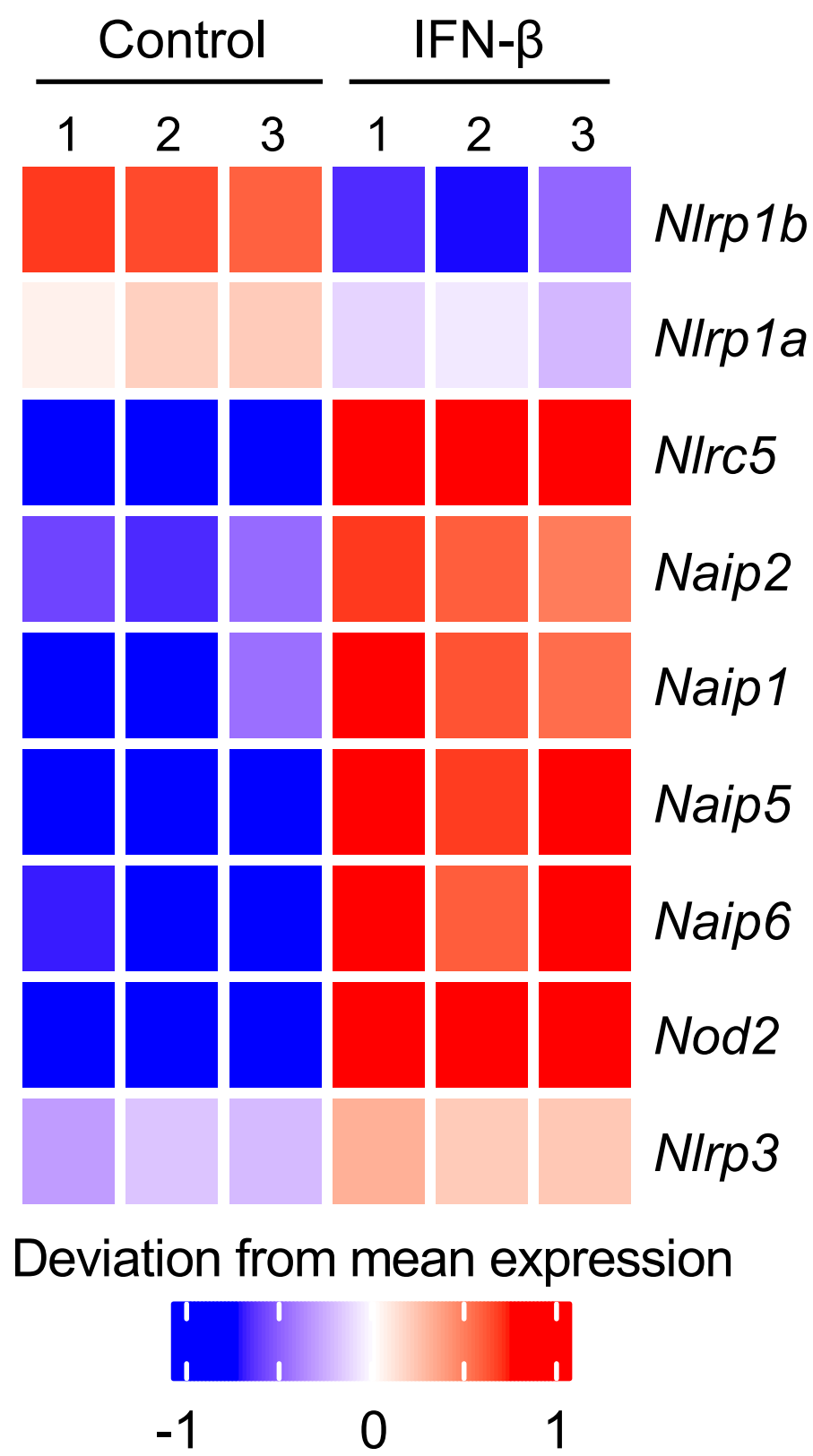

Additionally, several images related to viability assays could be consolidated. Streamlining these figures would reduce redundancy and potentially condense the presentation to fewer than four figures without compromising clarity.

Here, the reviewer is recommending rearranging figure panels to condense the presentation. In the current manuscript, we provide a step-by-step assessment of each molecule in the signaling pathway from LPS to the TLR4-TRIF-IRF3–mediated type I IFN production to the downregulation of NLRP1. We have separated each figure according to specific portion of the pathway being investigated, and each figure conveys one message that follows logically to the next figure. We feel keeping the figures separated as they are will aid reader comprehension.

Despite these concerns, the mechanistic advances presented in this manuscript are appreciated and contribute meaningfully to the field.

We thank the reviewer for noting that "the mechanistic advances presented in this manuscript are appreciated and contribute meaningfully to the field."

Minor comments:
• Line 57: The structure and function of VBP should be explained for clarity.

As requested by the reviewer, we have discussed these points in our revised manuscript.

• Line 74: The phrase "unexpectedly" is vague and should be revised or better contextualized.

As requested by the reviewer, we have clarified why we initially expected LPS to stimulate NLRP1, not inhibit it.

• In the discussion: The inhibitory role of type I interferon is not entirely unexpected, as antagonism between type I interferon and classical NF-κB-driven inflammatory responses has been well documented, particularly in the context of tuberculosis and COVID-19.

As requested by the reviewer, we have discussed this concept in our revised manuscript.

1st Revision - Editorial Decision 16th Jun 2026

Dear Dr. Kanneganti,

Thank you for the submission of your revised manuscript to our editorial offices. I have now received the reports from two of the three referees that were asked to re-evaluate the study, you will find below. Original referee #3 was completely unresponsive to my invitations to re-assess the study. However, going through your p-b-p-response, I consider the points by referee #3 and the advisor as adequately addressed. As you will see, the remaining two referees now support the publication of the study in EMBO reports.

Before we can proceed with formal acceptance, I have the editorial requests below I ask you to address in a final revised manuscript. Please also provide a final p-b-p-response to the editorial requests.

Editorial requests:

- We received bounced email alerts for the following co-authors:

Harisankeerth Mummareddy - sankee.mummareddy@stjude.org
Roman Sarkar - roman.sarkar@stjude.org

They need to be remove from the author list and added back using their new email addresses during re-submission. Otherwise, please provide their new email addresses upon re-submission and we will update their author accounts accordingly.

- Please provide the abstract written in present tense throughout.

- We will publish your manuscript in the report format. For a Scientific Report we require that results and discussion sections are combined in a single chapter called "Results & Discussion". Please do this for your manuscript. For more details, please refer to our guide to authors:

https://link.springer.com/journal/44319/submission-guidelines#cms-Article-types

- Please name the supplemental figures 'Figure EV1', 'Figure EV2' etc., and combine some of the present supplemental figures to have not more than 4-5 final Expanded View Figures. Please update all callouts and also the source data folders accordingly.

- Please order the manuscript sections like this, using only these names:
Title page - Abstract - Keywords - Introduction - Results & Discussion - Methods - Data availability section - Acknowledgements - Disclosure and Competing Interests Statement - References - Figure legends - Expanded View Figure legends

Please move the funding information to the acknowledgements.

- We now use CRediT to specify the contributions of each author in the journal submission system. CRediT replaces the author contribution section. Please use the free text box to provide more detailed descriptions and do NOT provide your final manuscript text file with an author contributions section. See also our guide to authors (section 'Author contributions'): https://link.springer.com/journal/44319/submission-guidelines#cms-Revised-submissions

- The 'Data Availability' section is restricted to information on large externally deposited datasets. If no such primary datasets have been deposited, please state this in this dedicated (e.g. 'No primary datasets have been generated and deposited'). Please remove any other text from this section.

- Please check again that the number "n" for how many independent experiments were performed, their nature (biological versus technical replicates), the bars and error bars (e.g. SEM, SD) and the test used to calculate p-values is indicated in the respective figure legends (main and EV figures). Please also check that all the p-values are explained in the legend, and that these fit to those shown in the figure. Please provide statistical testing where applicable. Please avoid the phrase 'independent experiment' but clearly state if these were biological or technical replicates. Please also indicate (e.g. with n.s.) if testing was performed, but the differences are not significant. In case n=2, please show the data as separate datapoints without error bars and statistics. See also:

https://link.springer.com/journal/44319/submission-guidelines#cms-Figure-and-data-presentation

If n<5, please show single datapoints for diagrams. Moreover:
- Please note that the exact p values are not provided in the legends of figures 1B, E, G; 2B, D; 3B, E; 4B, C, D, E, F; 5C, D, E, F; S1B, S2B, S3B, S4B, D; S6A, B.

- All Materials and Methods need to be described in the main text using our 'Structured Methods' format, which is required for all

research articles. According to this format, the Methods section should include a Reagents and Tools Table (listing key reagents, experimental models, software, and relevant equipment and including their sources and relevant identifiers), uploaded as separate file, and a Methods section in which we encourage the authors to describe their methods using a step-by-step protocol format with bullet points, to facilitate the adoption of the methodologies across labs. More information on how to adhere to this format as well as downloadable templates (.doc) for the Reagents and Tools Table can be found in our author guidelines (section 'Structured Methods'):

https://link.springer.com/journal/44319/submission-guidelines#cms-Manuscript-organisation-and-formatting

Please move the primer information (Supplemental Table 1) to the reagents and tools table and remove this table from the manuscript files. Please update the callouts and also add callouts to the Reagents & Tools Table where appropriate.

- Please confirm that for all Western blot panels shown the loading control was run on the same gel as the other proteins detected. Please note that we discourage comparisons between samples on different gels/blots, even if the samples derive from one experiment, as confounding factors reduce comparability. If unavoidable, the figure legend must state that the samples derive from the same experiment and that gels/blots were processed in parallel. If a 'representative' loading control is shown for multiple gels/blots, the intra-gel controls should be shown in the source data files, and the figure legends should describe the data displayed accurately. See our author guidelines:

https://link.springer.com/journal/44319/submission-guidelines#cms-Figure-and-data-presentation (section 'Electrophoretic gels and blots').

In addition, I would need from you uploaded separately:
- a short, two-sentence summary of the manuscript (not more than 35 words).
- two to four short (!) bullet points highlighting the key findings of your study (two lines each).
- a schematic summary figure as separate file that provides a sketch of the major findings (not a data image) in jpeg or tiff format (with the exact width of 550 pixels and the exact height of 300 pixels) that can be used as a visual synopsis on our website. Please upload this separately and remove the graphical abstract from the manuscript text file.

I look forward to seeing the further revised version of your manuscript when it is ready. Please let me know if you have questions regarding the revision.

Best,

Achim Breiling
Senior Editor
EMBO Reports

Referee #1:

In the revised manuscript, the authors improved the presentation of the findings and included additional additional on the transcriptional regulation of Nlrp1a in different cell types by type I IFNs. The scope of the findings remains largely unaltered but the new data provide additional support for the original claims. Certainly a lot more could be done to decipher the molecular details on how IFN signaling changes the Nlrp1 chromatin locus (or mRNA), but taken for what it is, I think the manuscript has met the bar for publication and will be of interest to those who specialize in innate immunity. Again I defer to the editor's opinion on the level of conceptual advance and novelty.

The authors performed an siRNA experiment to confirm the role of Nlrp1 in human macrophages/monocytes and observed a mild difference. Some sort of positive control gene eg CARD8 or a rescue would make it more rigorous but I guess the data are sufficient to support the limited claim in the paper.

Referee #2:

In my view the authors have addressed enough concerns for this to be now published in EMBO Reports. The paper clarifies the role of TLR4 and IFN in regulation of NLRP1, and present robust data in both mouse and human cells.

The authors have addressed all minor editorial requests.

2nd Revision - Editorial Decision **14th Jul 2026**

Dr. Thirumala-Devi Kanneganti
St Jude Children's Research Hospital
Department of Immunology
262 Danny Thomas Place
Mail Stop # 351
Memphis, TN 38105
United States

Dear Dr. Kanneganti,

I am very pleased to accept your manuscript for publication in the next available issue of EMBO reports. Thank you for your contribution to our journal.

Your manuscript will be processed for publication by EMBO Press. It will be copy edited and you will receive page proofs prior to publication. Please note that you will be contacted by Springer Nature Author Services to complete licensing and payment information.

You may qualify for financial assistance for your publication charges - either via a Springer Nature fully open access agreement or an EMBO initiative. Check your eligibility: https://link.springer.com/journal/44319/how-to-publish-with-us

Should you be planning a Press Release on your article, please notify the production team via this form in order to coordinate publication and release dates: https://forms.cloud.microsoft/e/eH4A8JVP6g

If you have any questions, please do not hesitate to contact the Editorial Office. Thank you for your contribution to EMBO Reports.

Yours sincerely,

Achim Breiling
Senior Editor
EMBO Reports

>>> Please note that it is EMBO Reports policy for the transcript of the editorial process (containing referee reports and your response letter) to be published as an online supplement to each paper. If you do NOT want this, you will need to inform the Editorial Office via email immediately. More information is available here: https://link.springer.com/partners/embo-press/editorial-policies#Peer%20review

EMBO Press Author Checklist

Corresponding Author Name: Thirumala-Devi Kanneganti	
Journal Submitted to: EMBO Reports	
Manuscript Number: EMBOR-2025-61867-T	

USEFUL LINKS FOR COMPLETING THIS FORM
The EMBO Journal - Author Guidelines
EMBO Reports - Author Guidelines
Molecular Systems Biology - Author Guidelines
EMBO Molecular Medicine - Author Guidelines

Reporting Checklist for Life Science Articles (updated January
This checklist is adapted from Materials Design Analysis Reporting (MDAR) Checklist for Authors. MDAR establishes a minimum set of requirements in transparent reporting in the life sciences (see Statement of Task: 10.31222/osf.io/9sm4x). Please follow the journal's guidelines in preparing your
Please note that a copy of this checklist will be published alongside your article.

Abridged guidelines for figures
1. Data
The data shown in figures should satisfy the following conditions:
- the data were obtained and processed according to the field's best practice and are presented to reflect the results of the experiments in an accurate and unbiased manner.
- ideally, figure panels should include only measurements that are directly comparable to each other and obtained with the same assay.
- plots include clearly labeled error bars for independent experiments and sample sizes. Unless justified, error bars should not be shown for technical
- if n<5, the individual data points from each experiment should be plotted. Any statistical test employed should be justified.
- Source Data should be included to report the data underlying figures according to the guidelines set out in the authorship guidelines on Data

2. Captions
Each figure caption should contain the following information, for each panel where they are relevant:
- a specification of the experimental system investigated (eg cell line, species name).
- the assay(s) and method(s) used to carry out the reported observations and measurements.
- an explicit mention of the biological and chemical entity(ies) that are being measured.
- an explicit mention of the biological and chemical entity(ies) that are altered/varied/perturbed in a controlled manner.
- the exact sample size (n) for each experimental group/condition, given as a number, not a range;
- a description of the sample collection allowing the reader to understand whether the samples represent technical or biological replicates (including how many animals, litters, cultures, etc.).
- a statement of how many times the experiment shown was independently replicated in the laboratory.
- definitions of statistical methods and measures:
  - common tests, such as t-test (please specify whether paired vs. unpaired), simple χ2 tests, Wilcoxon and Mann-Whitney tests, can be unambiguously identified by name only, but more complex techniques should be described in the methods section;
  - are tests one-sided or two-sided?
  - are there adjustments for multiple comparisons?
  - exact statistical test results, e.g., P values = x but not P values < x;
  - definition of 'center values' as median or average;
  - definition of error bars as s.d. or s.e.m.

Please complete ALL of the questions below.
Select "Not Applicable" only when the requested information is not relevant for your study.

Materials

Newly Created Materials	Information included in the manuscript?	In which section is the information available? (Reagents and Tools Table, Materials and Methods, Figures, Data Availability Section)
New materials and reagents need to be available; do any restrictions apply?	Not Applicable	

Antibodies	Information included in the manuscript?	In which section is the information available? (Reagents and Tools Table, Materials and Methods, Figures, Data Availability Section)
For **antibodies** provide the following information:<br>- Commercial antibodies: RRID (if possible) or supplier name, catalogue number and or/clone number<br>- Non-commercial: RRID or citation	Yes	Materials and Methods, Immunoblot Analysis subsection/Methods table

DNA and RNA sequences	Information included in the manuscript?	In which section is the information available? (Reagents and Tools Table, Materials and Methods, Figures, Data Availability Section)
**Short novel DNA or RNA including primers, probes**: provide the sequences.	Yes	Materials and Methods, qPCR subsection/Methods table

Cell materials	Information included in the manuscript?	In which section is the information available? (Reagents and Tools Table, Materials and Methods, Figures, Data Availability Section)
**Cell lines:** Provide species information, strain. Provide accession number in repository **OR** supplier name, catalog number, clone number, and/**OR** RRID.	Not Applicable	
**Primary cultures:** Provide species, strain, sex of origin, genetic modification status.	Yes	Materials and Methods/Methods table
Report if the cell lines were recently **authenticated** (e.g., by STR profiling) and tested for mycoplasma contamination.	Not Applicable	

Experimental animals	Information included in the manuscript?	In which section is the information available? (Reagents and Tools Table, Materials and Methods, Figures, Data Availability Section)
**Laboratory animals or Model organisms:** Provide species, strain, sex, age, genetic modification status. Provide accession number in repository **OR** supplier name, catalog number, clone number, **OR** RRID.	Yes	Materials and Methods, Mice subsection/Methods table
**Animal observed in or captured from the field:** Provide species, sex, and age where possible.	Not Applicable	
Please detail **housing and husbandry conditions**.	Yes	Materials and Methods, Mice subsection/Methods table

Plants and microbes	Information included in the manuscript?	In which section is the information available? (Reagents and Tools Table, Materials and Methods, Figures, Data Availability Section)
**Plants:** provide species and strain, ecotype and cultivar where relevant, unique accession number if available, and source (including location for collected wild specimens).	Not Applicable	
**Microbes:** provide species and strain, unique accession number if available, and source.	Not Applicable	

Human research participants	Information included in the manuscript?	In which section is the information available? (Reagents and Tools Table, Materials and Methods, Figures, Data Availability Section)
If collected and within the bounds of privacy constraints report on age, sex and gender or ethnicity for all study participants.	Not Applicable	

Core facilities	Information included in the manuscript?	In which section is the information available? (Reagents and Tools Table, Materials and Methods, Figures, Data Availability Section)
If your work benefited from core facilities, was their service mentioned in the acknowledgments section?	Not Applicable	

Design

Study protocol	Information included in the manuscript?	In which section is the information available? (Reagents and Tools Table, Materials and Methods, Figures, Data Availability Section)
If study protocol has been **pre-registered, provide DOI in the manuscript**. For clinical trials, provide the trial registration number **OR** cite DOI.	Not Applicable	
Report the **clinical trial registration number** (at ClinicalTrials.gov or equivalent), where applicable.	Not Applicable	

Laboratory protocol	Information included in the manuscript?	In which section is the information available? (Reagents and Tools Table, Materials and Methods, Figures, Data Availability Section)
Provide DOI OR other citation details if **external detailed step-by-step protocols** are available.	Not Applicable	

Experimental study design and statistics	Information included in the manuscript?	In which section is the information available? (Reagents and Tools Table, Materials and Methods, Figures, Data Availability Section)
Include a statement about **sample size** estimate even if no statistical methods were used.	Yes	Figure legends
Were any steps taken to minimize the effects of subjective bias when allocating animals/samples to treatment (e.g. **randomization procedure**)? If yes, have they been described?	Yes	Methods section, Cell Stimulation subsection
Include a statement about **blinding** even if no blinding was done.	Yes	Methods section, Cell Stimulation subsection
Describe **inclusion/exclusion criteria** if samples or animals were excluded from the analysis. Were the criteria pre-established?<br><br>If sample or data points were omitted from analysis, report if this was due to attrition or intentional exclusion and provide justification.	Not Applicable	
For every figure, are **statistical tests** justified as appropriate? Do the data meet the assumptions of the tests (e.g., normal distribution)? Describe any methods used to assess it. Is there an estimate of variation within each group of data? Is the variance similar between the groups that are being statistically compared?	Yes	Materials and Methods, Statistical Analysis subsection/Figure legends

Sample definition and in-laboratory replication	Information included in the manuscript?	In which section is the information available? (Reagents and Tools Table, Materials and Methods, Figures, Data Availability Section)
In the figure legends: state number of times the experiment was **replicated** in laboratory.	Yes	Figure legends
In the figure legends: define whether data describe **technical or biological replicates**.	Yes	Figure legends

Ethics

Ethics	Information included in the manuscript?	In which section is the information available? (Reagents and Tools Table, Materials and Methods, Figures, Data Availability Section)
Studies involving **human participants**: State details of **authority granting ethics approval** (IRB or equivalent committee(s), provide reference number for approval.	Yes	Materials and Methods section/Ethical Statement subsection
Studies involving **human participants**: Include a statement confirming that **informed consent** was obtained from all subjects and that the experiments conformed to the principles set out in the WMA Declaration of Helsinki and the Department of Health and Human Services Belmont Report.	Yes	Materials and Methods section/Ethical Statement subsection
Studies involving **human participants:** For publication of **patient photos**, include a statement confirming that consent to publish was obtained.	Not Applicable	
Studies involving experimental **animals**: State details of **authority granting ethics approval** (IRB or equivalent committee(s), provide reference number for approval. Include a statement of compliance with ethical regulations.	Yes	Materials and Methods section/Ethical Statement subsection
Studies involving **specimen and field samples:** State if relevant **permits** obtained, provide details of authority approving study; if none were required, explain why.	Not Applicable	

Dual Use Research of Concern (DURC)	Information included in the manuscript?	In which section is the information available? (Reagents and Tools Table, Materials and Methods, Figures, Data Availability Section)
Could your study fall under dual use research restrictions? Please check biosecurity documents and list of **select agents and toxins** (CDC): https://www.selectagents.gov/sat/list.htm	Not Applicable	
If you used a select agent, is the security level of the lab appropriate and reported in the manuscript?	Not Applicable	
If a study is subject to dual use research of concern regulations, is the name of the **authority granting approval and reference number** for the regulatory approval provided in the manuscript?	Not Applicable	

Reporting

The MDAR framework recommends adoption of discipline-specific guidelines, established and endorsed through community initiatives. Journals have their own policy about requiring specific guidelines and recommendations to complement MDAR.

Adherence to community standards	Information included in the manuscript?	In which section is the information available? (Reagents and Tools Table, Materials and Methods, Figures, Data Availability Section)
State if relevant guidelines or checklists (e.g., **ICMJE, MIBBI, ARRIVE, PRISMA**) have been followed or provided.	Not Applicable	
For **tumor marker prognostic studies,** we recommend that you follow the **REMARK** reporting guidelines (see link list at top right). See author guidelines, under 'Reporting Guidelines'. Please confirm you have followed these guidelines.	Not Applicable	
For **phase II and III randomized controlled trials**, please refer to the **CONSORT** flow diagram (see link list at top right) and submit the CONSORT checklist (see link list at top right) with your submission. See author guidelines, under 'Reporting Guidelines'. Please confirm you have submitted this list.	Not Applicable	

Data Availability

Data availability	Information included in the manuscript?	In which section is the information available? (Reagents and Tools Table, Materials and Methods, Figures, Data Availability Section)
Have **primary datasets** been deposited according to the journal's guidelines (see 'Data Deposition' section) and the respective accession numbers provided in the Data Availability Section?	Not Applicable	
Were **human clinical and genomic datasets** deposited in a public access-controlled repository in accordance to ethical obligations to the patients and to the applicable consent agreement?	Not Applicable	
Are **computational models** that are central and integral to a study available without restrictions in a machine-readable form? Were the relevant accession numbers or links provided?	Not Applicable	
If publicly available data were reused, provide the respective **data citations in the reference list**.	Yes	References
